# The UBX domain in UBXD1 organizes ubiquitin binding at the C-terminus of the VCP/p97 AAA-ATPase

**DOI:** 10.1038/s41467-023-38604-4

**Published:** 2023-06-05

**Authors:** Mike Blueggel, Alexander Kroening, Matthias Kracht, Johannes van den Boom, Matthias Dabisch, Anna Goehring, Farnusch Kaschani, Markus Kaiser, Peter Bayer, Hemmo Meyer, Christine Beuck

**Affiliations:** 1 Structural and Medicinal Biochemistry, Faculty of Biology, University of Duisburg-Essen, Essen, Germany; 2 Molecular Biology, Faculty of Biology, University of Duisburg-Essen, Essen, Germany; 3 Chemical Biology and ACE Analytical Core Facility Essen, Faculty of Biology, University of Duisburg-Essen, Essen, Germany

**Keywords:** Enzymes, Proteasome, Solution-state NMR, Mass spectrometry

## Abstract

The AAA+ ATPase p97/VCP together with different sets of substrate-delivery adapters and accessory cofactor proteins unfolds ubiquitinated substrates to facilitate degradation by the proteasome. The UBXD1 cofactor is connected to p97-associated multisystem proteinopathy but its biochemical function and structural organization on p97 has remained largely elusive. Using a combination of crosslinking mass spectrometry and biochemical assays, we identify an extended UBX (eUBX) module in UBXD1 related to a lariat in another cofactor, ASPL. Of note, the UBXD1-eUBX intramolecularly associates with the PUB domain in UBXD1 close to the substrate exit pore of p97. The UBXD1 PUB domain can also bind the proteasomal shuttling factor HR23b via its UBL domain. We further show that the eUBX domain has ubiquitin binding activity and that UBXD1 associates with an active p97-adapter complex during substrate unfolding. Our findings suggest that the UBXD1-eUBX module receives unfolded ubiquitinated substrates after they exit the p97 channel and before hand-over to the proteasome. The interplay of full-length UBXD1 and HR23b and their function in the context of an active p97:UBXD1 unfolding complex remains to be studied in future work.

## Abstract (web summary)

The function of VCP/p97 AAA-ATPase cofactor UBXD1 and its UBX domain has been elusive. Here the authors show that the extended UBXD1 UBX domain is located at the p97 pore exit where it binds ubiquitin, suggesting that UBXD1 receives unfolded substrates and hands them off for down-stream processing.

## Introduction

The conserved AAA+ ATPase p97 (also called VCP for Valosin-containing protein, or Cdc48 in yeast) is an abundant protein unfolding enzyme that is essential for a multitude of cellular protein homeostasis and signaling pathways. Missense mutations in VCP/p97 cause a multisystem proteinopathy (MSP-1) that features inclusion body myopathy, Paget’s disease of bone, fronto-temporal dementia and amyotrophic lateral sclerosis^1,2^. p97 contains two AAA+ ATPase domains, D1 and D2, that are arranged into two stacked hexameric rings with a central channel^3,4^. The regulatory N-domains are positioned at the periphery of the p97 hexamer and move up and down upon ATP hydrolysis in D1^5^. To achieve substrate unfolding, proteins are fed into the central pore at the D1 side, threaded through the central channel driven by ATP hydrolysis in D2, and released in an unfolded state from the D2 pore^6–8^.

In line with the central function of p97 in the ubiquitin-proteasome system, the majority of substrate proteins are targeted to p97 by poly-ubiquitination. Recent evidence suggests that ubiquitin chains serve as an unfolding tag and stay conjugated to the substrate proteins during unfolding^6,9,10^. This mechanism raises the question on how these ubiquitin moieties are handled after substrate exit from the p97 channel in order to mediate substrate degradation by the proteasome.

The function of p97 is modulated by a multitude of cofactor proteins, many of which contain ubiquitin-binding domains that are believed to help recruit substrate proteins^11^. Interaction of these cofactors with p97 relies on diverse sequence motifs or protein domains that mostly bind to either the N-domain or the C-terminal tail of p97^12^. The most prominent example is the UBX domain present in 13 of the human p97 cofactors^11^. It adopts a ubiquitin fold and generally binds to the p97 N-domain via a conserved FPR motif in the loop between strands β3 and β4. An exception is the UBX domain of UBXD1 (also called UBXN6) as degeneration of the FPR motif impairs p97 binding activity^13^. UBXD1 is also unique among the UBX-domain containing cofactors because of its simultaneous binding to the N- and C-terminal domains of p97. Binding to the p97 N-domain is achieved by a VCP/p97-interacting motif (VIM) in the N-terminus of UBXD1, thereby modulating p97 N-domain movements^14–16^. The PUB domain, positioned in the middle of the UBXD1 protein, binds to the p97 C-terminus and thus near the substrate exit pore^13,17^. The functional relevance of PUB domain binding to p97, however, is still unclear. Moreover, the function and structural arrangement of the UBX domain in the C-terminal region of UBXD1 has also remained elusive until now.

This lack of structural and mechanistic understanding contrasts with UBXD1’s physiological relevance. UBXD1 is directly linked to p97-associated multisystem proteinopathy because disease mutations at the p97 N-D1 interface affect the interaction with UBXD1 and thus UBXD1’s regulation of p97 N-domain movements and cellular functions^14,15^. In addition, human UBXD1 has critical roles in protein trafficking along the endosomal pathway and in the endo-lysosomal damage response (ELDR), facilitates mitophagy and regulates JAK-STAT1/2 signaling^14,16,18–20^. Moreover, the UBXD1 orthologue in flies, armless, functions as a regulator of wingless/WNT signaling^21^.

In this study, we applied cross-link mass spectrometry (XL-MS), biochemical and NMR interaction studies as well as structure prediction algorithms to study the structural arrangement of the UBX domain in UBXD1 and upon p97 binding. We identified a previously unrecognized extended UBX (eUBX) domain related to the eUBX of ASPL (also called UBXD9, ASPSCR1, or TUG) that associates with the UBXD1-PUB domain and conjointly binds the D2 side of the p97 hexamer. This differs from the only other well-studied eUBX of ASPL that binds to the p97 N-domain and regulates oligomerization^22^. We also found that UBXD1 binds ubiquitin via its UBX domain and can cooperate with a model substrate delivery complex suggesting a role in receiving an unfolded ubiquitinated substrate that exits the p97 channel at the D2 pore.

## Results

### UBXD1 binds ubiquitin through a region comprising the UBX domain

In contrast to homologous UBX domains, the UBX domain of UBXD1 does not serve as a p97 binding module due to a mutation in its p97 binding motif^13^. We hypothesized that the UBXD1-UBX domain may instead adopt a role as a ubiquitin binding module. To test this, we performed pulldown experiments to probe for ubiquitin binding both in cell lysates and with purified proteins.

**Fig. 1:**
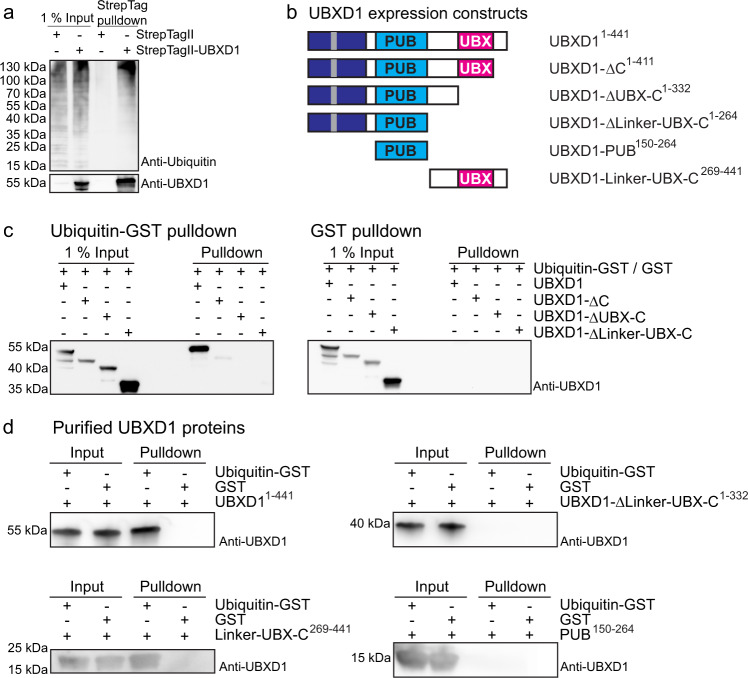
UBXD1 binds ubiquitin via its UBX domain. **a** Western blot analysis of cellular ubiquitin conjugates co-isolating in pulldowns of StrepTagII-UBXD1 or only the StrepTagII peptide expressed in HEK293 cells as indicated. In presence of UBXD1, ubiquitin conjugates were detected. **b** UBXD1 constructs overexpressed in HEK293 and *E. coli* cells to narrow down the ubiquitin binding region. Domains color-coded as follows: N-terminus (1–133, dark blue) with VIM helix (50–65, gray), PUB domain (150–264, light blue) and UBX domain (333–411, pink). **c** Deletion of the UBX domain (333–411) abolishes ubiquitin binding by UBXD1. UBXD1 or indicated truncation variants were expressed in HEK293 cells and lysates used for ubiquitin-GST (Ub-GST) pulldowns. Co-isolated UBXD1 was detected with UBXD1-specific antibodies. **d** Ub-GST pulldowns with purified UBXD1, UBXD1-ΔLinker-UBX-C, UBXD1-Linker-UBX-C or UBXD1-PUB. All blots (**a**, **c**, **d**) were performed as *n* = 1 independent experiments. For all GST control blots see Supplementary Fig. 1. Source data are provided as a source data file.

In a first approach, we isolated overexpressed StrepTagII-UBXD1 from HEK293 cells. Western blotting with a ubiquitin antibody revealed the typical pattern for a mixture of ubiquitin conjugates bound to UBXD1 (Fig. 1a, lane 4). To map the ubiquitin binding site in UBXD1, we expressed truncated variants of UBXD1 (Fig. 1b) and performed GST-pulldowns from cell lysates using a purified ubiquitin-GST fusion protein (Ub-GST). Full length UBXD1 (1–441, UBXD1-fl) and UBXD1-ΔC (1–411) lacking the C-terminus bound to Ub-GST (Fig. 1c), albeit UBXD1-ΔC was expressed and pulled down less efficiently. In contrast, UBXD1-ΔUBX-C (1–332, without UBX and C-terminus) or UBXD1-ΔLinker-UBX-C (269-332, without UBX, the preceding linker region and C-terminus) lost their ability to bind Ub-GST.

To confirm this more directly, we performed Ub-GST pulldowns with heterologously expressed and purified UBXD1 or isolated domains (Fig. 1d). Consistent with the previous experiments, Ub-GST only bound full length UBXD1 or a UBXD1-Linker-UBX-C construct, but not a UBX deletion mutant or an isolated PUB domain. Thus, UBXD1 possesses a previously unknown function as a ubiquitin binder via its C-terminal fragment containing the non-canonical UBX domain.

### Intramolecular interaction between UBXD1 domains

**Fig. 2:**
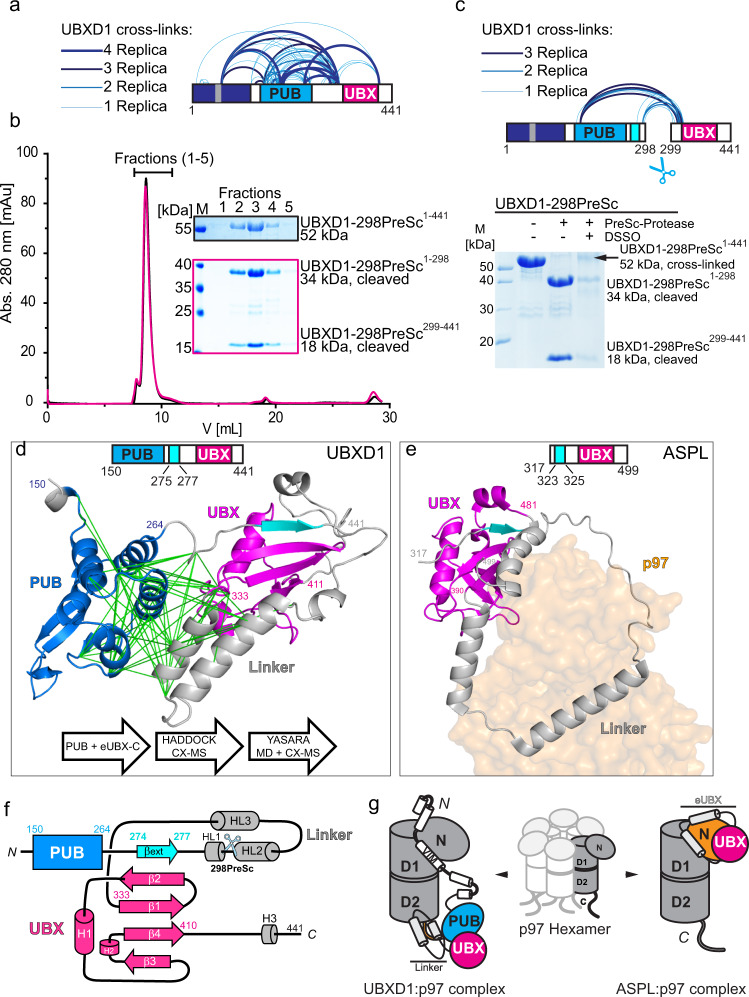
Intramolecular domain interactions within UBXD1 in the absence of p97. **a** Domain architecture of UBXD1 indicating all detected cross-links in *n* = 4 biologically independent samples (blue semi-circles; cross-linked amino acids, see Supplementary Data 2, FDR = 0.01). **b** Analytical SEC with UBXD1-298PreSc (black) and cleaved UBXD1-298PreSc fragments (magenta), *n* = 1 independent experiments. Upon cleavage of the PreSc protease recognition sequence (scissor symbol in 2c), two UBXD1-298PreSc fragments (1–298 and 299–441) are generated, which are still interacting on the analytical SEC. The fractions (1–5) were analyzed by SDS-PAGE. **c** Chemical cross-linking of the cleaved UBXD1-298PreSc fragments re-establishes a full length UBXD1-298PreSc-CX. All detected cross-links between these fragments are shown by blue semi-circles (top; *n* = 3 technical replicates; cross-linked amino acids see Supplementary Data 3, FDR = 0.01). **d** UBXD1-Linker-UBX-C resembles the ASPL extended UBX (eUBX) domain. Structural model of the PUB-Linker-UBX-C module (coordinates provided as Supplementary Data 1) based on the UBXD1-PUB structure (150–264, blue, pdb: 6SAP^17^) and the Linker-UBX-C (eUBX-C) iTASSER model (265–441, gray with canonical UBX, 333–411 in magenta). The beginning of the linker forms an additional β-stand (cyan) extending the β-sheet of the UBX domain. Both domains were docked using experimental cross-linking restraints (green lines) followed by MD simulation. **e** Crystal structure of ASPL bound to p97-ND1 (pdb: 5ifw^22^), eUBX (317–499, gray) with the canonical UBX domain (386–464) in magenta and β-sheet extension in cyan, p97-N domain in orange. **f** Secondary & tertiary structure scheme of UBXD1-PUB-Linker-UBX-C (PUB blue; linker gray with ß-sheet extension in cyan; core UBX magenta) **g** Schematic model of UBXD1 (1–441; left) and ASPL (313–553; right) binding to a p97 protomer. While the ASPL-eUBX domain binds to p97-N, the eUBX domain of UBXD1 is associated with the PUB domain which binds the p97-C-terminus. Source data are provided as a source data file.

The function of UBXD1-Linker-UBX-C as a ubiquitin binding module raises the question on how this domain is arranged within the structure of UBXD1. To probe for potential inter-domain interactions, UBXD1 was chemically cross-linked with disuccinimidyl sulfoxide (DSSO) followed by mass spectrometry analysis (XL-MS; Fig. 2a, Supplementary Fig. 2a and Supplementary Data 2). K166, K172, K193, and K257, located within the PUB domain, cross-linked either to K333 within the UBX domain or to K320 and K325 within the PUB-UBX linker region. In addition, cross-links were found within the PUB-UBX-linker region between K269, K301, K320, and K325. These results suggest that the PUB and UBX domains are in close proximity within UBXD1, pointing towards an intramolecular interaction. To verify this notion, we inserted a PreScission protease cleavage site at residues 298/299 within the PUB-UBX linker of UBXD1. The resulting protein UBXD1-298PreSc was efficiently cleaved with PreScission protease (Fig. 2c, SDS gels). Intriguingly, in gel filtrations the two parts (1–298 and 299–441) co-eluted with the same apparent size as full-length UBXD1 (Fig. 2b and Supplementary Fig. 2b), demonstrating that both fragments form intramolecular interactions within UBXD1. Consistent with this, chemical cross-linking of these fragments (Fig. 2c, Supplementary Data 3) generated a cross-link pattern similar to that of intact UBXD1 (Fig. 2a), mostly between K333 and Y334 with K166 and K269.

### UBXD1 contains an extended UBX (eUBX) domain

To inquire whether a similar interaction involving a homologous UBX domain is known, we performed a homology search of the UBXD1-Linker-UBX-C (251–441) fragment against the pdb data base using iTASSER^23–25^ and analyzed the UBXD1-fl AlphaFold^26, 27^ model (# Q9BZV1, Supplementary Fig. 2e). Both algorithms modeled the Linker-UBX-C fragment using the crystal structure of the highly homologous C-terminal part of ASPL bound to p97^22^ as template (pdb: 5ifs & 5ifw^22^, Fig. 2d–f and Supplementary Fig. 2d–f). The classic UBX fold consists of a 4-stranded β-sheet flanked by two small helices (Fig. 2d–f and Supplementary Fig. 2e, magenta). UBXD1 residues 274–277 of the linker between PUB and UBX form an additional β-strand that docks to β-strand 2 of UBX (Fig. 2d–f and Supplementary Fig. 2e, cyan), turning the remaining linker into a lariat. This Linker-UBX module was named extended UBX (eUBX) domain^22^. Therefore, we will refer to UBXD1 Linker-UBX (265–411) as eUBX and the Linker (265–332) alone as UBX extension.

We then docked the PUB domain (150–264; pdb: 6sap^17^) to our model using the 22 crosslinks between PUB and the eUBX-C iTASSER model as restraints, satisfying 95.5% of the crosslinks. After connecting PUB and the linker, the resulting model was subjected to a MD simulation which closed the loop and brought its two α-helices close together (Fig. 2d and Supplementary Data 1). In the ASPL:p97 structure^22^, the lariat loops around the p97-N domain and thus presents an open conformation which is reflected in our initial iTASSER and the AlphaFold model (Supplementary Fig. 2 f).

The crosslinks place the PUB domain close to the linker, which is not observed in the AlphaFold model. We, therefore, tested in a fluorescence anisotropy titration whether a direct interaction between eUBX-C (269–441) and PUB (150–264) exists, but no binding was detected (Supplementary Fig. 2c). In the PreScission experiment (Fig. 2b), the β-strand that extends the UBX β-sheet remains with the PUB domain and maintains binding UBX, which explains why PUB and UBX co-elute. Thus, the intramolecular interaction is solely due to the linker residues (265–298) without additional contributions by PUB.

Thus, these data provide evidence that the core UBX domain (333–411) and the preceding linker region (265–332) of UBXD1 (Fig. 2d, f) resemble the structure of the eUBX domain (317-389) of ASPL^22^ (Fig. 2e). The eUBX domain of ASPL interacts with the N-domain of p97 (Fig. 2e), which then induces disassembly of the p97 hexamer into its protomers. Instead, the eUBX of UBXD1 does not bind p97-N due to the substitution of the canonical F/P-P-R/K motif with GGQ^13,22^, likely explaining why UBXD1, in contrast to ASPL, does not disassemble the p97 hexamer^28^.

### Biochemical analysis of the interaction between UBXD1 and ubiquitin

Next, we biochemically characterized the interaction between UBDX1 and ubiquitin in detail using purified proteins. Isothermal titration calorimetry (ITC), fluorescence anisotropy, XL-MS, NMR spectroscopy and mutational analysis were employed to obtain binding constants and identify the binding epitopes on both binding partners.

**Fig. 3:**
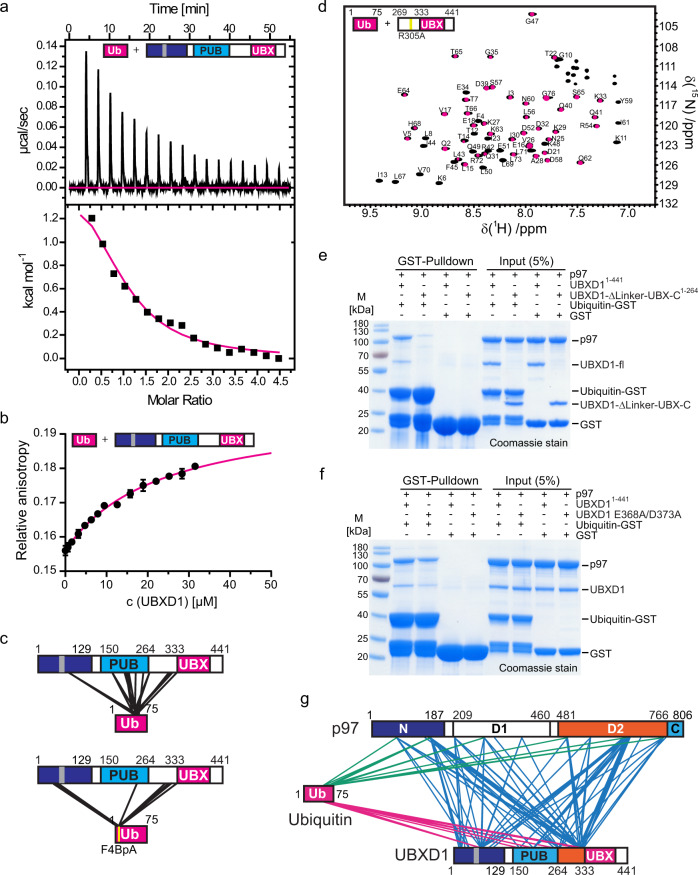
Characterization of the UBXD1:ubiquitin and p97:UBXD1:ubiquitin interaction. **a** ITC titration of UBXD1-wt with ubiquitin-wt shows a K_D_ of 18 ± 2 µM and a binding stoichiometry of *N* = 0.99 ± 0.03. **b** Fluorescence anisotropy titration of UBXD1 with Rhodamine-ubiquitin shows a dissociation constant of K_D_ = 23 ± 3 µM (*n* = 3 independent experiments. Data are represent as mean ± SD). **c** Chemical cross-linking of UBXD1 and ubiquitin with DSSO (left, only inter-molecular crosslinks are shown) and photo-reactive cross-linking with p-Benzoyl-L-phenylalanine (right). Reactive BpA is marked by a yellow line. (Cross-linked amino acids, see Supplementary Data 4, 5, FDR = 0.01). **d** ^1^H-^15^N-HSQC titration with ^15^N-labeled ubiquitin and UBXD1 (top; 0 µM black, 20 µM magenta). A decrease in signal intensity results from the increase in size upon UBXD1 binding. **e** Ubiquitin-GST pulls down a ternary complex of p97:UBXD1:Ub-GST with UBXD1-fl but not UBXD1-ΔLinker-UBX-C (*n* = 1 independent experiments). **f** UBXD1 E368A/D373A shows diminished binding in a pulldown with Ub-GST and p97 (*n* = 1 independent experiments. For screening of all tested mutants see Supplementary Fig. 4i). **g** Chemical cross-linking of ternary Ub-GST:UBXD1:p97 complex with DSSO (only inter-molecular crosslinks are shown; *n* = 1 technical replicates; cross-linked amino acids see Supplementary Data 8). Source data are provided as a source data file.

ITC verified endothermic binding of UBXD1 to ubiquitin with a K_D_ of 18 ± 2 µM (Fig. 3a), ΔH = 1664 ± 78 cal/mol and ΔS = 27.7 cal/mol/deg with a 1:1 stoichiometry (*N* = 0.99 ± 0.03). The K_D_ was confirmed by fluorescence anisotropy titrations with Rhodamine-ubiquitin (K_D_ = 23 ± 3 µM, Fig. 3b) and Atto594-ubiquitin (K_D_ = 16.9 ± 0.7 µM, Supplementary Fig. 3 f) revealing K_D_ values in the same micromolar range (Table 1). In contrast to wild type ubiquitin, ubiquitin-I44A, which abolishes binding to all known binding partners, and the ubiquitin-like protein SUMO1 did not bind to UBXD1 (Supplementary Fig. 3d, g), confirming the specific binding between UBXD1 and ubiquitin.

**Table 1:** Dissociation constants for binding of UBXD1 to different ubiquitin variants or SUMO1 as well as ubiquitin binding to UBXD1 fragments

Ubiquitin variants and chains with UBXD1	K_D_ [µM]
Ubiquitin	18 ± 2 µM*^a^
Ubiquitin-I44A	n.b.*^a^
Rhodamine-ubiquitin	23 µM ± 3 µM**^b^
Atto594-ubiquitin	16.9 ± 0.7 µM**^b^
Atto594-ubiquitin-M1C	25 µM ± 11 µM**^b^
Atto594-di-ubiquitin-M1C	21 µM ± 13 µM**^b^
Atto594-tri-ubiquitin-M1C	39 µM ± 24 µM**^b^
Atto594-tetra-ubiquitin-M1C	n.b.**^b^
Atto594-SUMO1	n.b.**^b^
**Ubiquitin with UBXD1 fragments**	
UBXD1-PUB (150–264)	n.b.*^c^
UBXD1-eUBX (263–330)	n.b.*^c^

*n.b.* no binding was detected.

independent experiments: *n = 1, **n = 3.

^a^measured by ITC titration.

^b^measured by fluorescence anisotropy titration.

^c^measured by ^1^H-^15^N-HSQC NMR titration.

To confirm the ubiquitin binding site within UBXD1, we cross-linked the UBXD1:ubiquitin complex with DSSO (Fig. 3c,top). The cross-linked residues of ubiquitin (K48, S57, Y59, K63, and T66) were found close to both the core UBX domain and the UBX extension of UBXD1 with cross-links to K269, K320, K333, and the PUB domain with K166, Y194, K198, K202, and K257, which form an intra-domain complex as described above. Due to its linker length, DSSO is expected to cross-link residues only at a distance of about 12 Å (like ubiquitin to PUB, adjacent to UBX). However, it cannot link residues in direct contact at a tight interface, which could explain why only few cross-links to the UBX domain were detected. Therefore, we also performed photo-cross-linking with a ubiquitin derivative in which residue F4 was replaced with a photo-reactive p-benzoyl-L-phenylalanine (BpA) moiety. The photo-cross-linked ubiquitin-F4BpA:UBXD1 complex formed multiple cross-links to UBXD1 residues P48, T49, A52, Q53, and M54 within the N-terminus, F223 within PUB, A321, E326, G330 in the UBX extension and Y334 within UBX (Fig. 3c, bottom). Intramolecular cross-links within the PUB-linker-UBX module are still present, showing that this structural unit remains intact upon ubiquitin binding.

To identify the residues involved in the ubiquitin:UBXD1 interaction, we also performed a ^1^H-^15^N-HSQC NMR titration of ^15^N-ubiquitin with full length UBXD1 and UBXD1-eUBX R305A (chosen because of its better expression an solubility compared to wt) (Fig. 3d and Supplementary Fig. 4). The size increase upon UBXD1 binding causes a global reduction of all signal intensities that is reflected by an average relative intensity <1^29^. An additional drop in intensity for some signals below the average and a chemical shift perturbation Δδ above the average indicate specific binding. Both chemical shift perturbations and signal intensities for UBXD1-fl and UBXD1-eUBX R305A highlight the interacting ubiquitin residues 4–8, 14–15, 29–32, 41–51, and 69–72 (Supplementary Fig. 4b, c, e & f). This identified patch contains the known protein interacting residues of ubiquitin^30^, including K6, L8, R42, I44, A46, K48, Q49, E51, and V70 (Fig. 3d and Supplementary Fig. 4g). To test if UBXD1-PUB (150–264) or the UBX extension (264–332) contribute to ubiquitin binding, we performed NMR titrations with the isolated PUB domain and the UBX extension. None of these fragments bound to ubiquitin (Table 1, Supplementary Fig. 5) supporting the notion that the UBX domain is involved in ubiquitin binding. UBXD1-eUBX R305A (K_D_ = 155 ± 14 µM) both bind ubiquitin with a significantly higher K_D_ compared to UBXD1-fl. The isolated eUBX domain shows a high propensity to precipitate. This can be caused by an improperly folded sub-population or the presence of invisible soluble aggregates and thus an over-estimated active concentration in the binding assay.

We next assessed ubiquitin binding by UBXD1 in the context of the ternary complex including p97. We first confirmed that Ub-GST pulls down a ternary Ub-GST:UBXD1:p97 complex with full length UBXD1 and that this was dependent on the UBXD1-Linker-UBX-C (Fig. 3e). Thus, UBXD1 can bind ubiquitin thought its Linker-UBX-C domain concomitantly with p97 and thus physically link p97 to a ubiquitylated substrate. Chemical crosslinking of the ternary complex isolated via size exclusion chromatography (Fig. 3g) shows crosslinks between UBXD1 and p97 that are consistent with the ones observed for the binary p97:UBXD1 complex. Moreover, intramolecular UBXD1 crosslinks show that the compact structure of the C-terminal PUB-eUBX module remains intact in the p97:UBXD1:Ub complex. Importantly, ubiquitin crosslinked mostly the PUB-eUBX region of UBXD1 as observed for the binary UBXD1:Ub complex.

Based on the interaction between the Linker-UBX-C module confirmed by the crosslinks in the ternary complex, we probed several patches of surface-exposed residues in this region by mutational analysis in the context of recombinant UBXD1-fl (E368A/D373A, R274A, Q278A/F286A/L288A, R305A, R307A, R305A/R307A/V316A). Mutants and wild type UBXD1 were screened in a pulldown assay for their ability to bind Ub-GST in the presence of p97 (Supplementary Fig. 4i). Importantly, only UBXD1 E368A/D373A specifically showed reduced ability to pull down with Ub-GST and recruit p97 (Fig. 3f and Supplementary Fig. 4i). This effect is consistent with the AlphaFold model of ubiquitin bound to the α-helical face of the eUBX domain (Supplementary Fig. 4j). Thus, our experiments establish the eUBX (Linker-UBX-C) domain of UBXD1 as ubiquitin binding module that links p97 to a ubiquitylated substrate in the context of the Ub-GST:UBXD1:p97 complex.

### UBXD1 binds short ubiquitin chains

The fact that UBXD1 contains a ubiquitin binding site within the eUBX domain raises the question what role this binding may play in the context of p97-mediated unfolding of ubiquitylated substrate proteins. UBXD1 has been linked to various cellular processes that mostly involve processing of substrates with K48-linked ubiquitin chains^19,31,32^.

Therefore, we investigated the interaction of UBXD1 with K48-linked poly-ubiquitin chains with a defined length. To obtain these derivatives, a chain synthesis of partially Atto594-labeled K48-linked ubiquitin was performed and the different Ub-chain lengths were separated by SEC (Supplementary Fig. 6). The isolated Ub-chains were then used in fluorescence anisotropy titrations with UBXD1, revealing that the dissociation constant increased with chain length from 21–25 µM for mono- and di-ubiquitin to 39 µM for tri-ubiquitin, while K48-tetra-ubiquitin barely bound at all (Supplementary Fig. 6, Table 1). This indicates that UBXD1 prefers short ubiquitin chains (Ub_1-3_), which is consistent with the 1:1 stoichiometry obtained from the ITC titration with mono-ubiquitin. Of note, while p97 targets proteins with long ubiquitin chains, these chains are cut down by associated deubiquitinating enzymes during p97-mediated processing^6^.

### Orientation of the UBXD1 C-terminal region (265–441) relative to p97

UBXD1 binds p97-N (1–199) with its N-terminus (1–133), while the UBX domain is located in close proximity to the PUB domain which binds p97-C (766–806). Therefore, the UBX domain is expected to be placed close to the p97-D2 pore. We tested this notion with two approaches: First, the domain arrangement of UBXD1 on p97 was probed by XL-MS. Second, a detailed biochemical analysis measuring binding constants with various truncation constructs was performed.

**Fig. 4:**
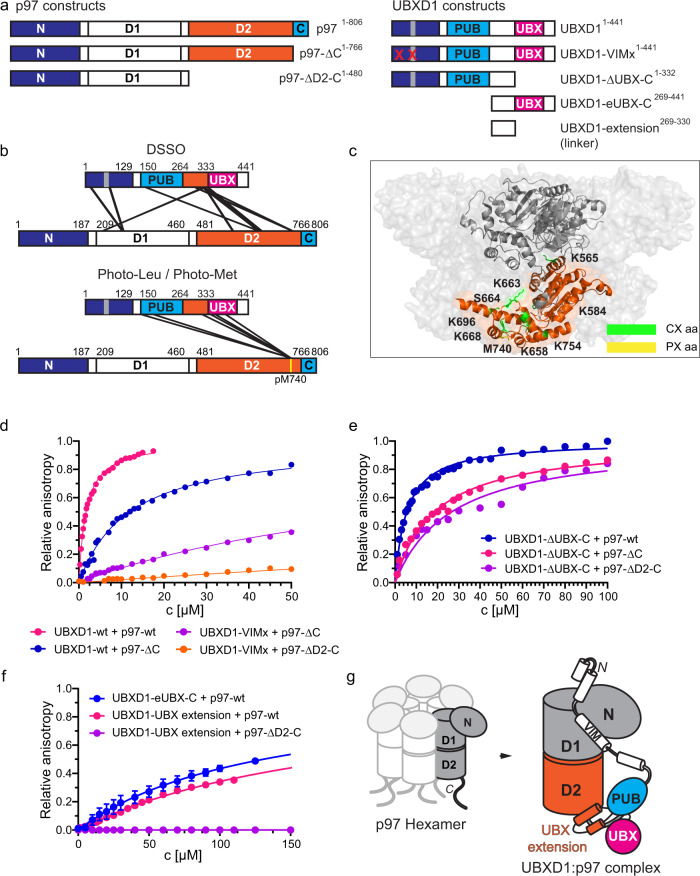
Biochemical mapping of the UBXD1:p97 domain interactions. **a** Domain architecture schemes of p97 and UBXD1 constructs used in fluorescence anisotropy titrations. **b** Scheme of the domain architectures of UBXD1 and p97 highlighting sites of chemical cross-linking by DSSO (CX; *n* = 3 technical replicates; cross-linked amino acids see Supplementary Data 6, FDR = 0.01) and photo-cross-linking by photo-Leu/photo-Met (PX; *n* = 3 technical replicates; cross-linked amino acids see Supplementary Data 7, FDR = 0.01). Cross-links between UBXD1 and p97 are indicated by straight lines. Reactive photo-Met 740 (pM740) is marked by a yellow line. c: Cross-linked amino acids from CX (green) and PX (yellow) highlighted in the p97-D2 domain (481–763, orange) in one protomer (pdb: 3cf3^45^). **d** Binding isotherms from fluorescence anisotropy titrations of UBXD1-wt (1–441) and -VIMx (1–441, DI11/12AA, RL62/63AA) with p97-wt (1–806), -ΔC (1–766) and -ΔD2-C (1–480) (UBXD1-wt + p97-wt: *n* = 3 independent experiments; all other data sets: *n* = 1 independent experiments. Data are represented as mean ± SD). **e** Binding isotherms of the UBX deletion mutant UBXD1-ΔUBX-C (1–332) with p97-wt (1–806), −ΔC (1–766) and −ΔD2-C (1–480) (UBXD1-ΔUBX-C + p97-wt *n* = 1 independent experiments; UBXD1-ΔUBX-C + p97-ΔC and UBXD1-ΔUBX-C + p97-ΔD2C *n* = 3 independent experiments. Data are represented as mean ± SD). **f** Binding isotherms of the isolated eUBX and the UBX extension (linker) with p97 (UBXD1-eUBX-C + p97, *n* = 3; UBXD1-UBX extension + p97-wt: *n* = 3 independent experiments; UBXD1-UBX extension + p97-ΔD2-C, *n* = 1 independent experiments. Data are represented as mean ± SD). **g** Suggested orientation of the C-terminal domains of UBXD1 (PUB, UBX extension and UBX domain) relative to p97. Source data are provided as a source data file.

Interactions between the p97 domains and UBXD1 were mapped by cross-linking the p97:UBXD1 complex with DSSO and identifying their contacts by XL-MS (Fig. 4b top and 4c; Supplementary Data 6). Cross-links were found between the N-terminus of UBXD1 (K66) and the N-/D1-domain of p97 (K211, K386; Fig. 4b top) supporting the published interaction of these regions^15,33^. p97-C does not contain DSSO-reactive residues, therefore no cross-links to UBXD1-PUB could be observed. The UBX extension of UBXD1 cross-linked to the p97 D2 domain (481–766) (Fig. 4b top and Supplementary Data 6). Intramolecular cross-links within the Linker (K134 to K269, and K333) and between PUB and the Linker (T163 to K325) showed that their intramolecular association also persists in the p97 bound state.

In addition, we performed photo-cross-linking with p97 enriched with photoreactive leucine and methionine (Supplementary Data 7). Most importantly, the photo-cross-linked UBXD1:pL/pM-p97 complex showed multiple cross-links between the photoreactive pM740 of p97, located at the bottom of p97-D2, with residues of UBXD1-PUB (V175), the UBX extension (L306, R332) and the UBX domain (Y336, L359) (Fig. 4b bottom and 4c).

Taken together, our cross-linking experiments confirmed that the PUB-eUBX module maintains its compact arrangement when bound to p97. With UBXD1-PUB binding p97-C and additional contacts between the UBX extension of UBXD1 and p97-D2, this supports the notion that the UBX domain is placed near the exit of the p97 hexamer pore.

Since this arrangement of two multi-domain proteins is highly complex, we validated the contacts observed by XL-MS by using a comprehensive biochemical mapping of the interactions with truncation constructs and mutations in different regions of both proteins. A potential interaction of the UBXD1-eUBX-C domain (265–441) with the D2 domain of p97 can be probed by sequentially eliminating the known interactions between UBXD1 and p97, and K_D_s were determined by fluorescence anisotropy titrations (Supplementary Table 1, Fig. 4d and Supplementary Fig. 3). To prevent binding of UBXD1 to the p97 C-terminus and/or D2 domain, p97 deletion mutants were used (p97-ΔC, 1–766 and p97-ΔD2-C, 1–480). The interaction between UBXD1-N and p97-N can be abolished with a set of mutations, called VIMx (DI11/12AA, RL62/63AA)^33^.

Elimination of the p97-C:UBXD1-PUB interaction (K_D_ = 11.9 ± 0.2 µM; Supplementary Table 1) resulted in a slightly higher affinity compared to the affinity for the p97-N:UBXD1-N isolated domain interactions (K_D_ 22 ± 1 µM; Supplementary Table 1, Supplementary Fig. 3a; K_D_ = 21 µM in Trusch et al. ^33^). With additional VIMx mutations that also prevent the p97-N:UBXD1-N interaction, binding was still observed with a K_D_ = 87 ± 1 µM. With both known interactions eliminated, this affinity must result from an additional, previously unknown contact between UBXD1 and p97. Further deletion of the p97 D2 domain drastically reduced binding (K_D_ = 466 ± 9 µM) which further confirmed that the new interaction site on p97 is located in the D2 domain.

To further define this new interaction, we investigated an UBX deletion mutant of UBXD1 (UBXD1-ΔUBX-C, 1–332) binding to truncations of p97 (Supplementary Table 1, Fig. 4e). While the affinity to p97-ΔD2-C (1–480) corresponds to the one observed for the isolated N-domain interaction (K_D_ = 26 ± 2 µM), the affinity to p97-ΔC containing the D2 domain was improved (K_D_ = 18.6 ± 0.7 µM), demonstrating that p97-D2 confers an additional contact with UBXD1.

The analysis of p97-wt binding to different UBXD1 deletion mutants suggests that the UBX extension can bind to the D2 domain of p97. Indeed, we found that both the eUBX-C domain (269–441, K_D_ = 131 ± 14 µM; Supplementary Table 1) and isolated UBX extension (269–330, K_D_ of 191 ± 2 µM; Supplementary Table 1) bound to p97. In contrast, upon deletion of the D2 domain (p97-ΔD2-C, 1–480) the UBX extension no longer bound to p97 (Fig. 4f). These data confirm that the UBX extension in UBXD1 is conferring additional contacts to p97-D2.

Thus, cross-linking and biochemical analysis demonstrated that the UBXD1-UBX domain, as part of the PUB-eUBX module, is located at the bottom of p97-D2 close to the p97 pore exit (Fig. 4g).

### Influence of UBXD1 on p97 activity

We previously found that UBXD1 inhibits the p97 ATPase activity by binding of the UBXD1-N domain to the p97 N-domain^15,33^ which appears inconsistent with a positive role of UBXD1 in substrate processing by p97. However, ATPase inhibition was measured in the absence of substrate and adapters. Substrate adapters like Ufd1:Npl4 bind to p97-N and some additionally to the D1 domain where they recruit substrates for p97 unfolding via binding of their ubiquitin chains^34–36^ and are expected to compete with UBXD1-N binding. One possibility is therefore that UBXD1-N may detach from p97 once a substrate adapter with a substate is bound to p97-N, leaving UBXD1 bound only to the p97-C-terminus though the PUB-eUBX unit and lifting ATPase inhibition. Currently, it is unknown how substrates that are processed by UBXD1 are targeted to p97. We, therefore, studied the effect of UBXD1 on a p97 unfolding complex, choosing the substrate adapter complex Ufd1:Npl4 as a model system because it is well established in vitro.

**Fig. 5:**
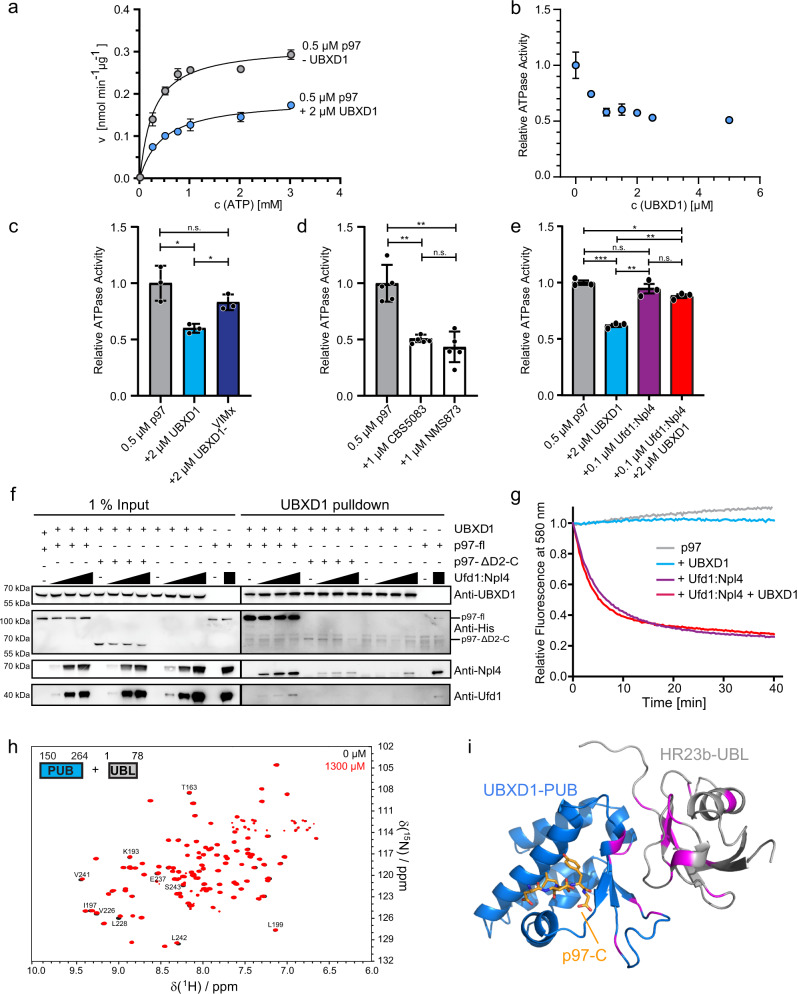
UBXD1 forms a ternary complex with p97 and a model substrate adapter Ufd1:Npl4. **a** Michaelis–Menten kinetics of p97 ATP hydrolysis without (gray) and with (blue) UBXD1 (2 µM) (*n* = 3 independent experiments. Data are represented as mean ± SD), showing that v_max_ is lower in combination with UBXD1. **b** Relative ATPase activity of p97 in presence of increasing UBXD1 concentrations (*n* = 3 independent experiments. Data are represented as mean ± SD). **c** Effect of the UBXD1-VIMx mutation on inhibition by UBXD1 (*n* = 3 independent experiments. Data are represented as mean ± SD. p97 ↔ p97 + UBXD1-wt, *p* = 0.040; p97 ↔ p97 + UBXD1-VIMx, *p* = 0.189; p97 + UBXD1 wt ↔ p97 + UBXD1-VIMx, *p* = 0.014). Note that the inhibition of p97 ATPase activity by UBXD1-wt is relieved by VIMx mutation. **d** Control measurements with chemical inhibitors of p97: CB5083 and NMS853 (*n* = 3 independent experiments. Data are represented as mean ± SD. p97 ↔ p97 + CB5083, *p* = 0.002; p97 ↔ p97 + NMS853, *p* = 0.0004; p97 + CB5083 ↔ p97 + NMS853, *p* = 0.282). **e** In presence of Ufd1:Npl4, a much lower inhibitory activity of UBXD1 on p97 ATPase activity is observed (*n* = 3 independent experiments. Data are represented as mean ± SD. p97 ↔ p97 + UBXD1, *p* = 0.0002; p97 ↔ p97 + Ufd1:Npl4, *p* = 0.331; p97 ↔ p97 + UBXD1 + Ufd1:Npl4, *p* = 0.011; p97 + UBXD1 ↔ p97 + Ufd1:Npl4, *p* = 0.010; p97 + UBXD1 ↔ p97 + UBXD1 + Ufd1:Npl4, *p* = 0.0004; p97 + Ufd1-Npl4 ↔ p97 + UBXD1 + Ufd1:Npl4, *p* = 0.256). Data c-e was analyzed by a Shapiro–Wilk-Test followed by a two-sided Welch-Test. n.s. indicates not significant, *p < 0.05, **p < 0.01, ***p < 0.001. **f** Substrate Adapter (Ufd1:Npl4) and UBXD1-TwinStrep can bind p97-fl simultaneously but compete for p97-ΔD2-C. Purified proteins were pulled down with StrepTactin beads (*n* = 1 independent experiments). Ufd1:Npl4 co-precipitate with UBXD1-TwinStrep dependent on p97-fl and only compete at increasing concentrations for p97-ΔD2-C. **g** UBXD1 does not inhibit p97:Ufd1:Npl4 mediated protein unfolding. p97 (500 nM hexamer), UBXD1 (2 µM), Ufd1:Npl4 (2 µM) were incubated with a polyubiquitylated Eos-based model substrate protein in the presence of ATP (3 mM). Loss of Eos fluorescence reflects substrate unfolding (*n* = 1 independent experiments). Unfolding was dependent on Ufd1:Npl4 and was not affected by the presence of UBXD1. **h** ^1^H-^15^N-HSQC titration of ^15^N-labeled UBXD1-PUB (400 µM) and HR23b-UBL (0 µM black, 1300 µM red). Shifting of signals indicate binding. Signals with the most pronounced chemical shift perturbation are labeled. **i** Structural model of the p97-C:UBXD1-PUB:HR23b-UBL complex docked with HADDOCK based on NMR chemical shift perturbations. (UBXD1-PUB in blue, p97-C in orange, HR23b-UBL in gray, residues used as docking restraints in magenta). Source data are provided as a source data file.

In the absence of substrate delivery cofactors, UBXD1 acted as a non-competitive inhibitor of the p97 ATPase activity and decreased the maximal velocity to 60% (Fig. 5a–d). The inhibition constant of K_i_ = 1.84 µM corresponds well to the dissociation constant of UBXD1:p97 with K_D_ = 1.64 µM (Supplementary Table 2). In contrast, the UBXD1-VIMx mutant, in which the N-terminus cannot bind p97, displayed a reduced effect (relative activity: 0.83 ± 0.06; Fig. 5c), indicating that the N-terminus of UBXD1 confers the main inhibitory function on the p97 ATPase activity. To test whether UBXD1 maintains its inhibitory function in the presence of a substrate adapter, we first measured the ATPase activity of p97 in different combinations with or without UBXD1 and the model adapter Ufd1:Npl4. UBXD1 alone conferred a decreased relative p97 activity of 0.62 ± 0.02. In contrast, in simultaneous presence with Ufd1:Npl4, UBXD1 displayed much lower p97 ATPase inhibition (relative activity: 0.88 ± 0.02, Fig. 5e, Supplementary Table 2). This effect is comparable to the slight inhibition imparted by the UBXD1-VIMx mutant, which implies that at least the N-domain of UBXD1 is released in the presence of Ufd1:Npl4 to relieve the inhibition of ATPase activity.

To distinguish whether only UBXD1-N or the entire UBXD1 dissociates from p97 upon substrate adapter binding, we performed a co-immunoprecipitation of purified Strep-tagged UBXD1 with p97 (fl or ΔD2-C) and Ufd1:Npl4. Ufd1:Npl4 co-isolated with UBXD1 dependent on the presence of p97-fl (Fig. 5f). With increasing Ufd1:Npl4 concentration, constant amounts of p97-fl bound, showing that UBXD1 does not compete with Ufd1:Npl4 but can bind p97 simultaneously, as long as it can remain attached to the p97 C-terminus via the PUB domain. In contrast, in p97-ΔD2-C, where UBXD1 can only bind via its N-terminus to p97-N, Ufd1:Npl4 competes with UBXD1 with increasing concentrations, resulting in reduced amounts of p97-ΔD2-C co-eluting with UBXD1. Npl4 shows some non-specific binding to the beads, but its presence could also be explained if only one or two UBXD1 N-termini are replaced on a p97 hexamer with three UBXD1 molecules bound^37^. Less p97-ΔD2-C is pulled down compared to p97-fl even in the absence of Ufd1:Npl4 because the interaction of N-domains^33^ is weaker than UBXD1-PUB binding to p97-C^17^.

To confirm that UBXD1 can cooperate with an active p97 complex and does not interfere with the p97-mediated protein unfolding process, we performed a previously established unfolding assay based on loss of Eos fluorescence in a poly-ubiquitinated model substrate^6^. Of note, the Ufd1:Npl4:p97 complex showed the same unfolding velocity regardless of the presence or absence of UBXD1 (Fig. 5g) even at concentrations that inhibited ATPase activity in the absence of substrate and adapter, demonstrating that UBXD1 does not negatively affect protein unfolding and consistent with the notion that it may regulate events downstream of unfolding.

### UBXD1 connects p97 to the proteasome

Our data suggest a role of UBXD1 in the post-unfolding processing of ubiquitylated p97 substrates. A homologous PUB domain in Peptidyl N-Glycanase (PNGase 1) was found to bind to the UBL domain of the proteasome shuttle factor HR23/RAD23, linking p97 via PNGase to the proteasomal machinery^38^. We, therefore, investigated whether UBXD1-PUB interacts with the proteasome shuttle factor HR23b-UBL domain. The ^1^H-^15^N-HSQC NMR titration of ^15^N-UBXD1-PUB with HR23b-UBL shows distinct chemical shift perturbations for residues T163, K193, V226, L228, E237, L242, and S243 (Fig. 5h and Supplementary Fig. 5d, e), mapping to the β-sheet face of UBXD1-PUB, with an average K_D_ = 1.5 ± 0.9 mM. The reverse titration of ^15^N-HR23b-UBL with UBXD1-PUB shows smaller, but still distinct chemical shift perturbations for HR23b-UBL residues L8, S32, V41, G43, Y48, A49, K51, L59, K60, I64, N68, T75, K76, and K78, located on the UBL β-sheet (Supplementary Fig. 5f, g). A structural model of the complex obtained by docking with HADDOCK (Fig. 5i) using the interacting residues from both domains confirms the β-sheet interface. In the UBXD1-PUB:HR23b-UBL complex the p97 binding pocket of UBXD1-PUB remains accessible (Fig. 5i). In addition, the interaction was confirmed by chemical cross-linking with DSSO and photo-cross-linking with a HR23b-UBL derivative where residue F69 was replaced by the photo-reactive p-benzoyl-L-phenylalanine (BpA) moiety (Supplementary Fig. 5h–I and Supplementary Data 9, 10). The interfaces of both UBXD1-PUB and HR23b-UBL and the low mM K_D_ are fully consistent with the PNGase-PUB:HR23-UBL complex^37^ and the requirement of HR23b to only transiently bind p97:UBXD1 for its shuttling function. Our data link the p97:UBXD1 complex to the proteasome via direct binding of UBXD1 to the proteasome shuttle factor HR23.

## Discussion

In this work, we have revealed new functional and structural features of the p97 cofactor UBXD1 that shed light onto the role of UBXD1 in assisting p97-mediated cellular processes. We identified a new module within UBXD1 stabilized by intramolecular interactions and involving the linker between the PUB and UBX domains as well as the core UBX domain itself. This extended UBX (eUBX) domain acts as a ubiquitin binding module and is placed in vicinity to the p97 exit pore suggesting that UBXD1 receives p97 substrate proteins after unfolding. Consistent with that, we demonstrate that, during p97-mediated substrate unfolding, UBXD1 can cooperate with a substrate-delivering adapter, which releases the inhibition of p97 ATPase activity by UBXD1 observed in the absence of a substrate protein.

The function of the UBXD1-UBX domain has been a mystery because it lacks the canonical F/P-P-R/K motif that enables other UBX domains to bind the p97 N-domain^13,28^. Instead, the UBXD1-N binds p97-N and the N-D1 interface^15,33^, which can inhibit p97 ATPase activity.

**Fig. 6:**
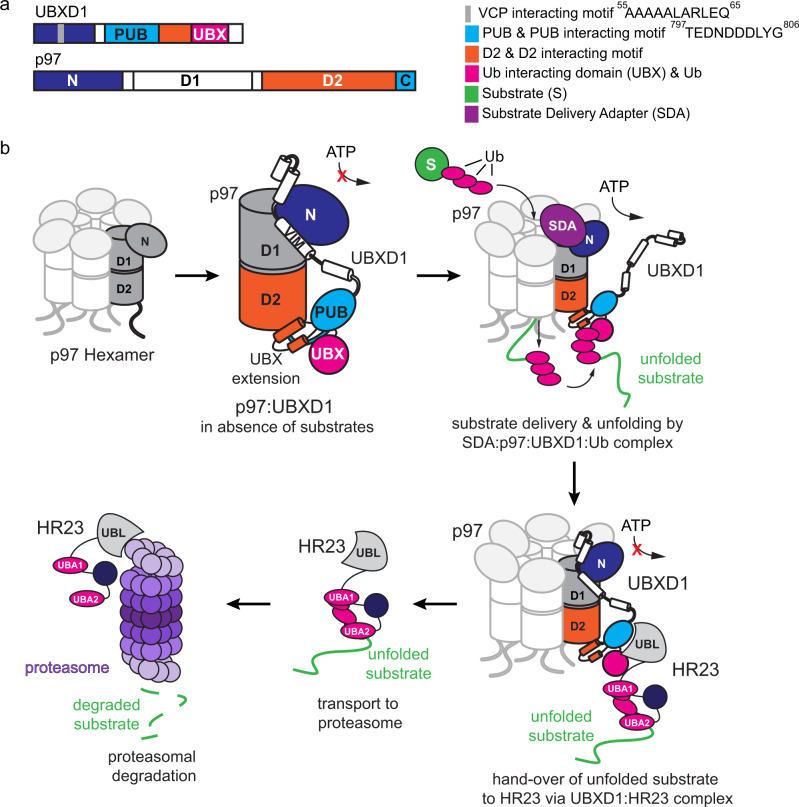
Model of the p97:UBXD1 interaction with ubiquitin and hypothetical functional implications. **a** Domain architecture of UBXD1 and p97 with interacting domains. While the UBXD1 N-terminus binds to the N domain of p97 and UBXD1-PUB binds to the C-terminal PIM of p97 (residues 797–806), the identified UBX extension interacts with the D2 ATPase domain. **b** UBXD1 wraps around p97 top-to-bottom. UBXD1-N locks the p97-N domain in down conformation and reduces ATP hydrolysis in the absence of substrate. UBXD1-PUB binds to the C-terminus of p97, supported by additional interactions between the UBXD1-UBX extension and p97-D2. This arrangement places the ubiquitin binding UBX domain close to the exit of the p97 hexamer pore. The inhibition conferred by UBXD1-N is released in the presence of substrate delivery cofactors, allowing unfolding to proceed. Substrates enter and exit the pore with their remaining ubiquitin units first, which quickly refold and are received by the UBXD1-UBX upon exiting the pore. Proteasome shuttle factor HR23 is recruited to the pore exit by binding to the PUB domain of UBXD1 to hand over the unfolded substrate for transport to and degradation by the proteasome.

We show that the UBXD1-UBX domain interacts with the linker between the PUB and UBX domains, thereby forming a structural eUBX unit. A similar UBX extension is also present in another p97 cofactor, ASPL. In the eUBX module, the β-sheet of the canonical UBX fold is extended by the beginning of the linker region, forming one additional β-strand. This results in a looping out of the remaining linker, which contains two α-helices in ASPL. We calculated a structural model also including the PUB domain based on the high sequence homology of the eUBX domains of UBXD1 and ASPL, the ASPL:p97 complex structure^22^ and our cross-linking data. Intriguingly, ASPL binds p97-N via its canonical UBX domain and loops the linker part of its UBX extension around p97-N, ultimately inducing dissociation of the p97 hexamer which renders p97 accessible to methylation by METTL21D^39^. We cannot exclude that the eUBX domain of UBXD1 may also act in a similar way under certain conditions. Moreover, we show here that UBXD1 can even cooperate with an active p97-adapter during substrate unfolding which makes it seem unlikely that UBXD1 breaks the hexameric p97 during the process. In contrast, we find that UBXD1-eUBX is located in close proximity to the PUB domain^28^, which binds the C-terminus of p97^13,17^ (Fig. 6). While the PUB domain is the primary binder anchoring the PUB-eUBX module to the p97 C-terminus, we could find that this arrangement allows additional interactions between the UBX extension and p97-D2 close to the substrate exit pore. Thus, the PUB-eUBX unit is located at the opposite end of p97 compared to the position of the ASPL eUBX domain. Our cross-linking data thereby suggest that UBXD1 closely wraps along p97 from its N- to C-terminus. Whether this implies a back-coupling mechanism communicating the state at the C-terminal p97 pore exit to the N-domain remains to be addressed in the future.

Importantly, however, we reveal that the eUBX domain possesses a previously unknown ubiquitin binding function that is located near the D2 pore which has interesting functional implications. Substrates enter the central channel with a ubiquitin moiety inserted first into the D1 pore of p97^10,40^. The ubiquitin and its attached substrate are then unfolded by processive threading through the channel from *cis* (D1) to *trans* (D2). Importantly, the ubiquitin chain, which may be trimmed by associated deubiquitinating enzymes^6,9^, stays attached to the substrate during threading and is expected to refold quickly upon exiting the p97 channel at the *trans* side. Based on our data, UBXD1 may receive the substrate by binding the short ubiquitin chain and trigger further downstream events such as substrate targeting to the proteasome. This model is consistent with our finding that UBXD1 can cooperate with a functional p97 complex during substrate unfolding and the UBXD1 PUB domain binding the UBL domain of the proteasomal shuttling factor HR23b. The interplay of full-length UBXD1 and HR23b and how they function together in the context of an active p97:UBXD1 unfolding complex remains to be studied in future work. Whereas UBXD1 alone can inhibit p97 ATPase activity^15,33^, we show that this inhibition is lifted by binding of substrate and substrate delivery adapter which competes for interaction with the p97 N-domain leaving UBXD1 bound solely to the p97 C-terminus. Of note, another p97 partner, PLAA also binds at the *trans* side of p97 and has ubiquitin binding activity^30,41,42^. It is therefore possible that PLAA and UBXD1 cooperate in regulating substrate processing downstream of p97-mediated unfolding. Consistent with that, both factors can bind simultaneously to p97 and together assist p97 in processing ubiquitylated proteins in the endo-lysosomal damage response^19,37^. Further functional and structural work will be required to validate these models, with focus on which substrate adapters collaborate with the p97:UBXD1 complex and the down-stream processing of unfolded substrates including delivery to the proteasome.

## Methods

### Site-directed mutagenesis (SDM)

UBXD1 (1–332; VIMx mutant: 1–441 DI11/12/AA, RL62/63AA; 269PreSc and 298PreSc protease site), ubiquitin (M1C, F4BpA) and p97 (1–766, 1–480) mutants were produced by site-directed mutagenesis with the primers listed in Supplementary Table 3 and Pfu-Plus! DNA polymerase (Roboklon, Berlin, Germany). SDM was performed with 20 ng DNA template, 2 mM dNTPs, 2 µM of each primer with 5 U Pfu-Plus! DNA polymerase. The template DNA was digested with 1 µL of Fast-Digest DpnI restriction enzyme (Thermo Scientific). The SDM for the UBXD1 alanine mutants was performed by BioCat GmbH, Heidelberg, Germany.

### Protein expression and purification

A list of all p97, UBXD1, ubiquitin, and HR23 expression plasmids can be found in Supplementary Table 4. Coding DNA sequences are provided in Supplementary Data 14.

### UBXD1-His and UBXD1-TwinStrep

For protein expression, plasmids were transformed into Rosetta (DE3) Escherichia coli cells and grown at 37 C in Terrific broth medium until they reached an OD600 0.8. Protein expression was induced by addition of 0.5 mM IPTG overnight at 18 °C. Cells were harvested and resuspended in wash buffer (50 mM Hepes, 150 mM KCl, 2 mM MgCl_2_, 5% glycerol, pH 7.4) which was supplemented with 25 mM imidazole (Sigma-Aldrich, 56750) for His-tagged UBXD1 (NiNTA wash buffer). Resuspended cells were incubated for 30 min with lysozyme (PanReac AppliChem, A3711), lysed by sonication (5 × 30 s at 60% intensity) and centrifuged at 20,000 × *g* for 45 min. Supernatant was filtered, loaded onto a HisTrap FF 5 ml column (Cytiva) or two StrepTrap HP 5 ml column (Cytiva), and washed with 40 column volumes of NiNTA wash buffer or 20 column volumes of wash buffer. Proteins were eluted with NiNTA elution buffer (wash buffer supplemented with 300 mM imidazole) or Strep elution buffer (wash buffer supplemented with 2.5 mM desthiobiotin) directly onto a GSTrap High Performance 5 ml (Cytiva) and washed with 15 column volumes of wash buffer (supplemented with 1 mM DTT). UBXD1 was eluted by cleaving off the GST-tag using PreScission protease. UBXD1 was further purified by size-exclusion chromatography using a HiLoad 16/600 Superdex 200 pg column equilibrated in wash buffer (supplemented with 1 mM DTT). All purification steps were carried out at 4 °C and proteins were concentrated using 10 kDa centrifugal concentrator (Vivaspin Turbo 15) before snap freezing in liquid nitrogen and stored at −80 °C.

### His-UBXD1-PUB

The N-terminal hexahistidine-tagged UBXD1-PUB domain (150–264)^17^ in a pET28a vector was expressed in *Escherichia coli* Rosetta2 in M9 minimal media with 0.6 g/L unlabeled or ^15^N-labeled ammonium sulfate as nitrogen source. After growing cells up to OD_600_ = 1.0 at 37 °C, protein expression was induced with 0.1 mM IPTG for 4 h at 37 °C. Pelleted cells were lysed in PBS buffer (pH 7.4) with 1 mM PMSF and lysozyme (1 mg/mL) in combination with sonication. His-UBXD1-PUB was purified by Ni-NTA affinity chromatography in PBS buffer (pH 7.4) and eluted with an imidazole gradient. The His_6_ tag was cleaved with thrombin protease prior to size exclusion chromatography in 50 mM NaKPi (pH 6.5), 150 mM NaCl. UBXD1-PUB was concentrated and the buffer exchanged to 50 mM NaKP_i_ (pH 6.5) in a centrifugal concentrator (5 kDa MWCO, Vivaspin).

### GST-UBXD1 constructs

All remaining UBXD1 constructs were expressed with an N-terminal GST tag. The DNA sequences of human UBXD1 (1–441) were cloned into a pGEX6P1 expression vector with N-terminal GST tag and a PreScission protease cleavage site using BamHI/EcoRI. The DNA sequence of the UBX extension (263–330) was cloned into a pET41b expression vector with a N-terminal GST tag and a PreScission protease cleavage site using ApaI/HindIII. All other GST-UBXD1 constructs were synthesized and cloned in pGEX6P1 with a Prescission protease site by Biocat (Heidelberg, Germany). Before transformation into *E. coli* Rosetta2 (DE3) (Sigma-Aldrich, Germany) the constructs were verified by Sanger sequencing (Seqlab, Göttingen, Germany). For production of unlabeled protein 10 ml TB-overnight culture (50 µg/mL Kanamycin or 100 µg/mL Ampicillin with 30 µg/mL chloramphenicol) was harvested, resuspended in 1 L TB-media and grown to OD_600_ = 1.2 at 37 °C, 160 rpm. After induction of protein expression by addition of 1 mM IPTG, cells were incubated for 20 h at 18 °C followed by centrifugation (4000 rpm, 20 min, 4 °C). For the expression of ^15^N isotope-labeled protein the bacteria were grown in 1 L M9 minimal media supplemented with 0.7 g/L [^15^N] ammonium chloride up to OD_600_ = 0.8. The UBX extension was expressed for 3 h at 37 °C, 160 rpm with 0.25 mM IPTG in M9 minimal media. Pelleted cells were dissolved in PBS-buffer, pH 7.4 supplemented with 1 mM PMSF, lysed with lysozyme (1 mg/mL) and disrupted by sonication. Upon ultracentrifugation (30,000 rpm, 60 min, 4 °C) the protein was purified by glutathione affinity chromatography with PBS-buffer, pH 7.4. PreScission (PreSc) protease digestion was used to cleave the GST tag prior to a size exclusion chromatography in PBS-buffer, pH 7.4. For the ATPase activity assay, all proteins were purified in buffers based on Tris (pH 7.5) instead of phosphate.

### p97

The N-domain of p97 (1–199)^33^ in a pET28a expression vector and all other p97 constructs in a pET15b expression vector yield an N-terminally His_6_-tagged product. For protein expression the p97 vectors were transformed into *E. coli* BL21 DE3 (T1r). 10 ml TB-overnight culture (100 µg/ml Ampicillin) were harvested, resuspended in 1 L TB-media and grown up to OD_600_ = 1.0 at 37 °C, 160 rpm. After induction of protein expression by addition of 1 mM IPTG, cells were incubated for 20 h at 18 °C followed by centrifugation (4000 rpm, 20 min, 4 °C). For the ATPase activity assay, all proteins were purified in buffers based on Tris (pH 7.5) instead of phosphate. For production of photo-leucine and photo-methionine (Thermo Scientific, Waltham, Massachusetts, USA) labeled p97, cells were grown in M9 minimal media in presence of 20 mg photo-Leu/Met per 1 L media. Cells were incubated as described for TB media. Pelleted cells were dissolved in 50 mM Tris/HCl (pH 7.4), 5 mM MgCl_2_ supplemented with 1 mM PMSF, lysed with lysozyme (1 mg/ml) and disrupted by sonication. Upon ultracentrifugation (35,000 rpm, 60 min, 4 °C) the protein was purified by Ni-NTA affinity chromatography in 50 mM Tris/HCl (pH 7.4), 5 mM MgCl_2_, eluting with an imidazole gradient, followed by anion exchange chromatography in 50 mM Tris/HCl (pH 7.4), 5 mM MgCl_2_, eluting with a KCl gradient.

### Ubiquitin

Ubiquitin-wt, ubiquitin-M1C, ubiquitin-I44A, StrepTagII-ubiquitin (pET23a, NdeI/HindIII, gene synthesis by BioCat GmbH, Heidelberg, Germany) and ubiquitin-GST were transformed into *E. coli* BL21 DE3 (T1r) and expressed as unlabeled proteins in TB-media. After induction of protein expression by addition of 0.4 mM IPTG, cells were incubated for 20 h at 18 °C. For the expression of ^15^N isotope-labeled ubiquitin, cells were grown in 1 L M9 minimal media up to OD_600_ = 0.8. After induction of protein expression by addition of 0.4 mM IPTG, cells were incubated for 20 h at 30 °C, followed by centrifugation (4000 rpm, 20 min, 4 °C). Pelleted cells were resuspended in PBS-buffer, pH 7.4 supplemented with 1 mM PMSF, lysed with lysozyme (1 mg/ml) and disrupted by sonication. Upon ultracentrifugation (30,000 rpm, 60 min, 4 °C) the protein was purified by perchloric acid precipitation followed by a strong cation exchange chromatography with 25 mM ammonium acetate (pH 4.6) and elution with 25 mM ammonium acetate, 200 mM NaCl (pH 7.5). For expression of ubiquitin-F4(p-Benzoyl-L-phenylalanine) (pET23a) for photo cross-linking the plasmid was transformed into *E. coli* BL21 DE3 (T1r) which contains a pEVOL-pBpF plasmid (addgene, 31190) and incubated in TB-media. The protein expression was started with 0.4 mM IPTG and 0.2% of L-Arabinose, in addition to p-Benzoyl-L-phenylalanine (1 mM, BpA in 1 M NaOH, for 20 h at 18 °C. Purification was performed as described above for the unlabeled protein.

### Enzymes for K48-linked poly-ubiquitin synthesis

Human mUbe1 (residues 1–924, mouse E1 enzyme, pET28a, His_6_-tagged, Thrombin protease site) and Ubc7 (residues 1–165, K48 E2 enzyme, pGEX6P1, GST-tagged, PreScission protease site) enzymes for the K48-linked poly-ubiquitin synthesis were transformed into *E. coli* Rosetta2 (DE3) (Sigma-Aldrich, Germany) and expressed in TB-media with 1 mM IPTG for 20 h at 18 °C. His6-mUbe1 was purified by Ni-NTA in tandem with an anion exchange chromatography (Q HP column) in 50 mM HEPES, 150 mM KCl, 2 mM MgCl_2_, pH 7.5. The protein was eluted from the Ni-NTA column with Imidazole followed by washing with the base buffer. The protein was subsequently eluted from the anion exchange column with a KCl gradient. GST-Ubc7 was purified with GSH affinity chromatography in 50 mM HEPES, 150 mM KCl, 10 mM MgCl_2_, pH 8.0 followed by a gel filtration (Superdex 75 pg 26/60).

### Enzymes for unfolding assay

For Ufd1 and Npl4 expression^36^, each plasmid was transformed into Rosetta (DE3) *Escherichia coli* cells and grown at 37 °C in TB medium until they reached an OD_600_ = 0.8. Protein expression was induced by addition of 0.5 mM IPTG at 18 °C overnight (for Npl4) or at 37 °C for 4 h (for Ufd1). Cells were harvested and resuspended in wash buffer (50 mM Hepes, 150 mM KCl, 3 mM MgCl_2_, 5% glycerol, 25 mM imidazole (Sigma-Aldrich, 56750), pH 7.4). Resuspended cells were mixed (Ufd1 + Npl4), incubated for 30 min with lysozyme (PanReac AppliChem, A3711), lysed by sonication (5 × 30 s at 60% intensity) and centrifuged at 20,000 × *g* for 45 min. The supernatant was filtered, loaded onto a HisTrap FF 5 ml column (Cytiva), and washed with 40 column volumes of wash buffer. Proteins were eluted with NiNTA elution buffer (50 mM Hepes, 150 mM KCl, 3 mM MgCl_2_, 5% glycerol, 300 mM imidazole, pH 7.4) and further purified by size-exclusion chromatography using a HiLoad 16/600 Superdex 200 pg column equilibrated in gel filtration buffer (50 mM Hepes, 150 mM KCl, 3 mM MgCl_2_, 5% glycerol, 1 mM DTT, pH 7.4). All purification steps were carried out at 4 °C and proteins were concentrated using 10 kDa centrifugal concentrator (Vivaspin Turbo 15) before snap freezing in liquid nitrogen and stored at −80 °C.

### HR23

HR23b-UBL wt (pGEX6P1) with N-terminal GST tag and a PreScission protease cleavage site (gene synthesis by BioCat GmbH, Heidelberg, Germany) was expressed in *E. coli* Rosetta 2 cells in TB medium (unlabeled) or M9 medium with ^15^N-ammonium chloride (^15^N- labeled for NMR) for 20 h at 30 °C with 0.5 mM IPTG. Cells were sonicated in PBS buffer pH 7.4 and the lysate was cleared by ultracentrifugation (30,000 rpm, 60 min, 4 °C). HR23b-UBL was purified by GSH affinity chromatography followed by PreScission cleavage of the GST tag and size exclusion chromatography. For expression of HR23b-UBL-F69BpA (p-Benzoyl-L-phenylalanine) (pGEX6P1, gene synthesis by BioCat GmbH, Heidelberg, Germany) for photo cross-linking the plasmid was transformed into *E. coli* BL21 DE3 (T1r) which contains a pEVOL-pBpF plasmid (addgene, 31190) and incubated in TB-media. The protein expression was started with 0.5 mM IPTG and 0.2% of L-Arabinose, in addition to p-Benzoyl-L-phenylalanine (1 mM, BpA in 1 M NaOH, for 20 h at 30 °C. Purification was performed as described above for the unlabeled protein.

### Poly-ubiquitin chain synthesis

K48-linked poly-ubiquitin chains were synthesized following a modified protocol published by ref. ^43^. A reaction mixture containing 12 mg Atto594-M1C-ubiquitin (final concentration 10 µM), with 5 µM E1 enzyme mUbe1 in reaction buffer (50 mM HEPES pH 8.0, 10 mM MgCl_2_, 10 mM ATP, 1 mM DTT) was preincubated for 15 min at 37 °C prior to the addition of 120 mg unlabeled ubiquitin (final concentration 1 mM) and 50 µM E2 enzyme Ubc7. The reaction mixture was incubated overnight at the same temperature. Reaction products were diluted 1:20 in 25 mM ammonium acetate (pH 4.6) and purified by strong cation exchange (SP HP 5 mL, GE Healthcare, elution with 200 mM NaCl, pH 7.5), followed by a size exclusion chromatography (HiLoad 26/60 Superdex 75 pg, GE Healthcare) in PBS buffer for final purification. The reaction products were analyzed by SDS-PAGE (4–20%, Mini-PROTEAN® TGX™ Precast Protein Gel, BioRad, California, USA) and scanned by using a Typhoon imager prior to Coomassie staining.

### Proteolytic interaction assay

For the interaction studies between UBXD1 fragments, a PreScission protease site was inserted into the UBXD1 sequence between positions E298 and E299. The resulting UBXD1-298-PreSc-His_8_ protein was purified and incubated with PreScission protease over night at 4 °C. Cleavage was confirmed by SDS PAGE. The cleaved protein was subjected to an analytical SEC experiment in 50 mM HEPES (pH 7.4), 150 mM KCl, 2 mM MgCl_2_, 5% Glycerol and 1 mM DTT on a Superdex 75 pg 10/300 column (GE Healthcare, Illinois, USA). Gel filtration standards (BioRad, Hercules, California, USA) were used to calibrate the SEC experiment. For the cross-linking with disuccinimidyl sulfoxide (DSSO) the DTT was removed by buffer exchange.

### Cell culture

HEK293 cells (Cellosaurus, RRID:CVCL_0045 [CLS], confirmed by Microsynth Seqlab, Switzerland in 2019) were grown first in Dulbecco’s modified Eagle’s media containing 10% fetal bovine serum (Cat. P04-04510, Pan Biotech) and 1% penicillin/streptomycin in a humidified atmosphere with 5% CO_2_ at 37 °C. At a density of 50–60% cells are transfected with the UBXD1 plasmids (pcDNA3.1(+), StrepTagII, wt with aa 1–441, 1–411, 1–332, or 1–264, Gene synthesis by BioCat GmbH, Heidelberg, Germany) or empty vector with a StrepTagII with 293Free reagent and incubated for 24 h for overexpression of UBXD1 and disrupted with lysis buffer (PBS buffer, 0.05% Igepal, pH 7.4, Halt protease and phosphatase inhibitor cocktail (Cat. 78440, Thermo Scientific, Germany). The cell lysate was cleared by centrifugation at 13.200 rpm for 1 h at 4 °C.

### Pulldown of UBXD1 with ubiquitin-GST / GST

Glutathione High Capacity Magnetic Agarose Beads (Cat. G0924, Sigma-Aldrich) were washed and blocked for 1 h at 4 °C with PD blocking buffer (PBS, 0.1% Triton X-100, 0.25% Lysozyme, pH 7.4). Then 3 mg of ubiquitin-GST or GST protein were added and incubated for 20 h at 4 °C. After several washing steps, 3 mg of HEK293 cell lysate with overexpressed UBXD1 proteins (UBXD1-fl, 1–441; UBXD1-ΔC, 1–411; UBXD1-ΔUBX-C, 1–332; UBXD1-ΔeUBX-C, 1–264) or 5 µM of recombinant UBXD1 or UBXD1-ΔUBX-C protein were incubated for 4 h at 4 °C. The beads were washed with PD buffer (PBS, 0.1% Triton X-100, pH 7.4) and bound proteins were eluted with SDS-PAGE sample buffer. For Western blot analysis, the proteins were eluted by boiling in SDS-PAGE sample buffer at 95 °C for 10 min.

### Pulldown of UBXD1 and p97 with ubiquitin-GST/GST

Reactions of 50 μM ubiquitin-GST or GST, 2.5 μM p97 (hexamer) and 5 μM UBXD1 variant were incubated with GSH beads (Pierce Glutathione Magnetic Agarose Beads, Thermo Fischer Scientific, Cat. 78601) for 30 min at 4 °C in IP buffer (50 mM Tris pH 7.4, 150 mM KCl, MgCl_2_, 2 mM β-Mercaptoethanol, 5% Glycerol, 1% Triton-X-100). Beads were washed with IP buffer and eluted by boiling in Laemmli sample buffer at 95 °C.

### Initial pulldown screening of UBXD1 mutants and p97 with ubiquitin-GST/GST

For protein expression, plasmids were transformed into Rosetta (DE3) Escherichia coli cells and grown at 37 °C in LB medium until they reached an OD600 0.8. Protein expression was induced by addition of 0.5 mM IPTG overnight at 18 °C. Cells were harvested and resuspended in wash buffer. Resuspended cells were lysed by sonication (1 × 60 s at 90% intensity) and centrifuged at 21,500 × *g* for 30 min. Supernatants were incubated with GSH beads for 30 min at 4 °C. UBXD1 variants were eluted in supernatant by cleaving off the GST-tag using PreScission protease. Reactions of 25 μM Ubiquitin-GST, 1 μM p97 (hexamer) and equal amounts of UBXD1 variant were incubated with GSH beads for 30 min at 4 °C in IP buffer. Beads were washed with IP buffer and eluted by boiling in Laemmli sample buffer at 95 °C.

### Pulldown of UBXD1-TwinStrep with p97 wt and ND1L

Reactions containing p97 variant (200 nM hexamer), UBXD1-TwinStrep (400 nM), BSA (2%), and Ufd1:Npl4 (0, 400, 2000, and 4000 nM as indicated) were incubated with StrepTactin Sepharose beads (Cat. 2-1201-010, iba) for 1 h at 4 °C in PD buffer (25 mM Hepes pH 7.4, 150 mM KCl, 2 mM MgCl_2_, 1 mM DTT, 5% Glycerol, 1% Triton-X-100) supplemented with 2% BSA. Beads were washed with PD buffer and eluted by boiling in Laemmli sample buffer at 95 °C.

### Western blot

Proteins were separated on a 4–20% Mini-PROTEAN TGX Gel (BioRad) and transferred to a nitrocellulose membrane (GE Healthcare, US) via tank blot. The membrane was blocked for 1 h with 5% milk powder in PBST, followed by three washing steps and incubation with Anti-UBXD1 antibody (1:1000, mouse, clone 2F8-24, Abcam ab81555, for all UBXD1 pulldowns except the pulldown with Ufd1:Npl4), Anti-UBXD1 (=anti-UBXN6, 1:500, mouse, clone 5C3-1, Acris Antibodies 5C3-1, for UBXD1 pulldown with Ufd1:Npl4), Anti-ubiquitinylated proteins antibody (1:2000, mouse, clone FK2, Merck 04-263), Anti-VCP/p97 antibody (1:1000, mouse, clone VCP 5, Santa Cruz sc-57492,), Anti-Npl4 (=anti-NPLOC4, 1:1000, rabbit polyclonal, Atlas HPA021560), Anti-penta-His antibody (1:1000, mouse, Qiagen 34660) or Anti-Ufd1 antibody (1:1000, mouse, clone 19/Ufd1L, Santa Cruz sc-81630) in 3% BSA in PBST or Anti-GST antibody (1:1000, mouse, clone 3D4, Santa Cruz sc-57753) in 5% milk powder in PBST for 20 h at 4 °C. Before incubation with the second antibody, membranes were washed three times with PBST, followed by incubation with the secondary antibody Anti-mouse IgG-HRP antibody (1:10,000, sheep polyclonal, Cytiva NXA931, for all pulldowns with mouse antibodies except the pulldown with Ufd1:Npl4), Anti-mouse IgG-(H + L)-HRP antibody (1:10,000, goat polyclonal, Biorad 1706516, for pulldown with Ufd1:Npl4), or anti-rabbit IgG (H + L)-HRP Conjugate (1:10,000, goat polyclonal, Biorad 1706515 for pulldowns with rabbit antibodies) in 3% BSA in PBST for 1 h at room temperature. After three additional washing steps, proteins were detected using SuperSignal West Pico PLUS or Femto Chemiluminescent Western Blot Detection (Thermo Fisher Scientific Inc, US).

### NMR-titration experiments

NMR experiments were recorded on a 700 MHz Ultrashield NMR spectrometer (Bruker) with a TCI inverse cryoprobe at 25 °C. Spectra were processed in Topspin 3.7 and analyzed with CARA^44^.

To ^15^N labeled protein in 50 mM sodium potassium phosphate buffer, pH 7.5, 90%/10% (v/v) H_2_O / D_2_O, the binding partner (UBXD1, PUB, UBX extension, ubiquitin) dissolved in the same buffer was added stepwise to their respective final concentration (Supplementary Tables 5–11). After each step a ^1^H^15^N-BEST-TROSY-HSQC spectrum was recorded. Relative signal intensities were calculated with the following equation:

1 $$\left(\frac{I}{{I}_{0}}\right)=\frac{I}{{I}_{0}}\cdot \left(\frac{V}{{V}_{0}}\right)$$

With: (I/I_0_) = relative residual intensity, I = signal intensity in the presence of the binding partner, I_0_ = intensity of spectrum without binding partner, (V/V_0_) = dilution factor with: V = volume of sample after addition of the binding partner, V_0_ = volume of sample without binding partner.

### Fluorescence anisotropy interaction studies

Fluorescence anisotropy measurements were carried out on a FP-8300 JASCO fluorescence spectrometer (JASCO GmbH, Pfungstadt, Germany). The UBXD1 proteins, SUMO1 and ubiquitin were labeled with Atto594-NHS ester (ATTO Tec.) in a ratio of 1:1.2. The M1C-ubiquitin was labeled with Atto594-maleimide (ATTO Tec.) with a 1:8 ratio of Atto594 in PBS buffer (pH 7.4) and 8× molar excess Tris(2-carboxyethyl)phosphine (TCEP). The N-terminally labeled Rhodamine-ubiquitin was ordered from BostonBiochem, Cambridge, MA, USA. Measurements of UBXD1:p97 were performed in 50 mM NaKP_i_ buffer, pH 7.5 and UBXD1:ubiquitin, SUMO1 in PBS buffer, pH 7.5 at 25 °C. All fitting curves have R^2^ values between 0.90 and 0.99. For the displacement titration UBXD1 was incubated with 0.1 µM 5,6-FAM-p97-C10 peptide (Sequence: 5,6-FAM-^797^TEDNDDDLYG^806^) to yield 50% saturation (as judged from a direct titration) and the concentration of p97 was successively increased.

Because the binding stoichiometries between the different p97 and UBXD1 constructs featuring deletions and/or inactivating mutations were not determined due to lack of material and might differ from the 6:3 stoichiometry of the full length wt proteins, we fitted all binding experiments with the stoichiometry *n* = 1 for comparability. Binding curves of the averaged data were fitted with GraphPad Prism 5.0 (GraphPad) using the quadratic binding equation for a one-site specific binding model:

2 $$r={r}_{0}+{r}_{\max }\cdot \frac{\left(\left(F+x+{K}_{D}\right)-\root{2}\of{\left({\left(F+x+{K}_{D}\right)}^{2}-\left(4\cdot x\cdot F\right)\right)}\right)}{2\cdot F}$$

with r = anisotropy, r_0_ = anisotropy without protein, r_max_ = maximum anisotropy, F = fluorescent probe (labeled peptide or protein) concentration, x = titrant protein concentration, K_D_ = dissociation constant.

For the displacement titration the averaged data were fitted with the following competitive binding model:

3 $$r={r}_{0}+\frac{\left({r}_{{{\max }}}-{r}_{0}\right)}{\big(1+{10}^{\left(x-{{\log }}\left({{{{{\rm{EC}}}}}}50\right)\right)}\big)}$$

4 $${{\log }}\left({{{{{\rm{EC}}}}}}50\right)={{\log }}\left({10}^{{{\log }}{{{{{{\rm{K}}}}}}}_{{{{{{\rm{D}}}}}}2}}\cdot \left(\frac{1+c({F}^{*})}{{K}_{D1}}\right)\right)$$

with r: anisotropy, r_0_: anisotropy without protein, r_max_: maximum anisotropy, x: titrant protein concentration of p97-wt in [LOG10[M]], c(F*): concentration of fluorescent labeled component p97-C10 peptide, K_D1_: dissociation constant of complex UBXD1:p97-C10 peptide determined from a direct titration, K_D2_: dissociation constant of complex UBXD1:p97-wt.

### Isothermal titration calorimetry (ITC)

ITC measurements were performed with a MicroCal iTC2000 (Malvern Instruments, Malvern, UK). UBXD1 at a concentration of 40 µM was titrated with a 1 mM stock solution of ubiquitin-wt or 0.98 mM of ubiquitin-I44A mutant at 25 °C in 1x PBS (pH 7.5). The binding parameters were calculated using MicroCal Origin 7.

### Immunoprecipitation

For Ufd1 immunoprecipitation 2 µg of Anti-Ufd1 antibody (clone: 5E2 Cat. sc-81630, Santa Cruz) were incubated with the recombinant proteins (5 µM Ufd1, 5 µM Npl4, 5 µM p97 and 2.5, 5 or 10 µM UBXD1) and Protein L Agarose Beads for 20 h at 4 °C in IP binding buffer (50 mM HEPES, 150 mM KCl, 0.1% Triton X-100, 0.25% Lysozyme, 1 mM DTT, pH 8.0). The agarose beads were washed several times and proteins were eluted with SDS-PAGE sample buffer.

### p97 substrate unfolding assay

The His_6_-Ub-Ub-mEos3.2 model substrate was generated by cloning mEos3.2 cDNA (gift from M. Davidson, Addgene #54550) C-terminally to two linear linked ubiquitin moieties. The substrate was further polyubiquitinated as described^45,46^ (Reagents kindly provided by R. Deshaies). Briefly, 10 µM substrate was mixed with 1 µM Ube1, 20 µM gp78-Ubc7 fusion enzyme and 100 µM ubiquitin in reaction buffer (50 mM HEPES pH 7.4, 150 mM KCl, 10 mM MgCl_2_, 5% Glycerol, 2 mM ATP) and incubated over night at 37 °C. Polyubiquitinated substrate was then purified by Ni-NTA affinity chromatography followed by size-exclusion chromatography (Superdex200 16/600, GE Healthcare). A break in the peptide backbone was subsequently introduced by photoirradiation with a longwave UV-lamp (Blak-Ray, B-100AP) for 2 h, while every 30 min a 15 min lasting non-irradiating interval was set. Unfolding was measured by mixing 40 nM of photoconverted UbUb2-mEos3.2 with 500 nM p97 (hexamer) and 2 µM Ufd1:Npl4 and/or 2 µM UBXD1 in reaction buffer (25 mM Hepes pH 7.4, 100 mM KCl, 5 mM MgCl_2_, 1 mM DTT). After incubation at 37 °C for 5 min, the reaction was started by addition of ATP (3 mM). and the change in fluorescence indicating substrate unfolding was measured every 15 s over 45 min (Ex.: 540 nm, Em.: 580 nm) in a Varian Cary Eclipse Fluorescence Spectrophotometer.

### ATPase activity assay

p97 was diluted in the assay buffer (50 mM Tris pH 8.0, 20 mM MgCl_2_, 1 mM EDTA) to a final volume of 0.2 mL with a concentration of 0.5 µM and incubated for 15 min at 37 °C with 3 mM ATP. The reaction was stopped by adding 0.8 mL of stopping solution (0.5% [w/v] SDS, 0.5% [w/v] ammonium molybdate, 2% [v/v] H_2_SO_4_) as described by ref. ^47^. The inorganic phosphate released by ATP hydrolysis was measured as phosphate-molybdate complex after 5 min incubation and subsequent addition of 10 µL of a 10% [w/v] solution of ascorbic acid at room temperature. The amount of inorganic phosphate released was calculated using a NaH_2_PO_4_ standard curve. The ATPase activity was measured with p97, p97:UBXD1-wt complex (0.5 µM p97 ± 2 µM UBXD1 + 0.25, 0.5, 0.75, 1, 2, 3 mM ATP; and 0.5 µM p97 + 0.5, 1, 1.5, 2, 2.5, 5 µM UBXD1), p97:UBXD1-VIMx complex (0.5 µM p97 + 2 µM UBXD1-VIMx mutant) and the unfolding complex combination with Ufd1/Npl4 (0.5 µM p97 ± 2 µM UBXD1 ± 0.1 µM Ufd1/Npl4). All assays were repeated at least three times and the average activities with standard errors of measurement are presented. The data was analyzed by a Shapiro–Wilk-Test followed by a two-sided Welch-Test. Values for K_M_ and v_max_ as well as the Lineweaver–Burk-representation were fitted with the software GraphPad Prism 5.0. As controls the chemical inhibitors of p97 CB5083 (Cat. M15705, Sigma-Aldrich) and NMS853 (Cat. SML1128, Sigma Aldrich) were tested with 1 µM each in five replicates^48^. The Michaelis–Menten kinetic parameters were fitted to the Michaelis–Menten equation using GraphPad Prism 5.0:

5 $$y=\frac{{v}_{{{\max }}}*x}{\left({K}_{m}+x\right)}$$

With: y = velocity [nmol P_i_ min^−1^ µg^−1^], x = ATP concentration [mM], v_max_ = maximal velocity [nmol P_i_ min^−1^ µg^−1^], K_m_ = Michaelis–Menten constant [mM].

The Lineweaver–Burk plot was created from the linearized data.

Michaelis–Menten parameters are used to calculate the inhibition constant of UBXD1 as follows:

6 $${K}_{i}=\,\frac{{v}_{{{\max }}}^{{{{{{\rm{app}}}}}}}}{{v}_{{{\max }}}}*\left(1+c\,\right)$$

with K_i_ = inhibition constant [µM], v_max_^app^ = v_max_ of p97 with cofactor/inhibitor UBXD1 [nmol Pi min^−1^ µg^−1^], v_max_ = v_max_ of p97 without cofactor/inhibitor [nmol Pi min^−1^ µg^−1^], c = molecular concentration of cofactor/inhibitor [µM].

### Model of the UBXD1-eUBX-C domain

A homology model for the C-terminal part of UBXD1 (251–441) was calculated using the software iTASSER^23–25^ using the UBXD1 (251–441) sequence as input. Molecular dynamics simulations of the homology model generated by iTASSER were performed using the YASARA Structure Suite (v11.12.31) with AMBER03 force field using the experimental intramolecular cross-links as restraints. Simulations were carried out in a cubic simulation cell of 85 Å edge length with an explicit water model, drift correction and periodic boundaries. Simulations were run at 298 K in water with 0.9% (m/m) NaCl, pH 7.4, with 2.4 fs step width and 0.8 nm Coulomb and van der Waals interactions cutoff. Snapshots were taken every 25 ps.

### Model of UBXD1-PUB-eUBX-C

The domain arrangement of the PUB-UBX fragment was modeled in HADDOCK using our NMR structure of the PUB domain (pdb: 6sap^17^) and the iTASSER-generated homology model of the UBX domain as separate structures. Inter-domain crosslinks obtained by XL-MS were used as unambiguous restraints for docking (Supplementary Table 12). The additional restraints listed in Supplementary Table 13 were added to keep the C-terminal E264 of the PUB domain in proximity of the N-terminal P265 of the UBX domain. Rigid body energy minimization generated 1000 initial complex structures, and the best 200 lowest energy structures were selected for torsion angle dynamics and subsequent Cartesian dynamics in an explicit water solvent. Default HADDOCK scaling for energy terms was applied. Applying the default scoring function, cluster analysis of the 200 water-refined structures yielded a single clear ensemble with a HADDOCK score of −105.6 + /− 1.3. PUB and UBX domains were subsequently connected in YASARA via loop modeling. All crosslinks involving residues in the loop were included in the simulation. Thus, three crosslinks (K320-KK325, K301-K325, K269-K325) were used as distance restraints in YASARA with minimum and maximum distance of 5 and 20 Å, respectively. The distances after MD are also listed in Supplementary Table 12. The coordinates of the resulting model are provided in pdb format (Supplementary Data 1).

### AlphaFold Model of ubiquitin bound to UBXD1-eUBX-C

The structural model of ubiquitin bound to UBXD1-eUBX-C was calculated using AlphaFold on the ColabFold v1.5.2 server (https://colab.research.google.com/github/sokrypton/ColabFold/blob/main/AlphaFold2.ipynb) with default parameters, using the sequences of ubiquitin and UBXD1-eUBX-C (269–441) as input.

### Model of the UBXD1-PUB:HR23b-UBL complex

The UBXD1-PUB:HR23b-UBL complex was modeled in HADDOCK using our NMR structure of the PUB domain^17^ (pdb: 6SAP) and the NMR solution structure of the HR23b-UBL domain^49^ (pdb: 1P1A). The following interacting residues were used as restraints based on their NMR chemical shift perturbation: UBXD1-PUB: K193, V226, L228, E237, L242, S243; HR23b-UBL: L8, S32, V41, G43, Y48, A49, K51, L59, K60, I64, N68, T75, K76, K78. The p97-C peptide was placed in the UBXD1-PUB binding pocket by alignment with the mouse PNGase-PUB:p97-C crystal structure^50^ (pdb: 2HPL) as described in ref. ^17^.

### Visualization of structural models

Molecular structural models were visualized using PyMol 1.3.

### Chemical cross-linking

Crosslinking reactions were optimized by varying disuccinimidyl sulfoxide (DSSO, Thermo Fischer Scientific, Cat. A33545, Waltham, MA USA 02451) concentration, protein concentration and crosslinking time and analyzed by SDS PAGE. The UBXD1 protein, the UBXD1:p97 and UBXD1:Ub protein complex of 10 µM UBXD1 and/or 50 µM ubiquitin and/or 25 µM p97 was cross-linked with a 25x molar excess of DSSO in PBS buffer (pH 7.5) for 15 min at 25 °C. The reaction was stopped with 20 mM Tris (pH 7.5) final concentration. Three independent technical experiments were analyzed for the binary DSSO complexes. For UBXD1 alone, the intramolecular DSSO cross-links found in four biological replicates were analyzed. For the ternary complex p97:UBXD1:Ub, 10 µM UBXD1, 25 µM p97 and 50 µM ubiquitin (500 µL) were cross-linked with 25x molar excess of DSSO for 15 min at 25 °C. After stopping the reaction with Tris (20 mM, pH 7.5), the cross-linked complex was isolated by size exclusion chromatography with a Superose 6 Increase 10/30 column (Cytiva) in PBS buffer (pH 7.5). High molecular weight fractions (>p97 hexamer) were combined and submitted to MX analysis. 50 µM UBXD1-PUB and 500 µM HR23b-UBL (330 µL) were cross-linked with 2x molar excess of DSSO for 25 min at 25 °C. After stopping the reaction with Tris (20 mM, pH 7.5), the cross-linked complex was isolated by size exclusion chromatography with a Superdex 75 column (Cytiva) in PBS buffer (pH 7.4) and cross-linked complex fractions were analyzed by MX.

### Photo-reactive cross-linking with p-Benzoyl-L-phenylalanine (BpA), photo-leucine/-methionine

A solution of 10 µM p97-pLeu/pMet and 25 µM UBXD1 was irradiated by UV light for 30 min on ice using a 3UV-8W UV lamp (UVP, Upland) at a wavelength of 365 nm. For BpA PX, 10 µM of UBXD1 was incubated with 50 µM, 125 µM, 250 µM or 2.5 mM Ub-F4BpA. 50 µM UBXD1-PUB was incubated with 0,25 mM, 0,5 mM, 0,75 mM, or 1 mM HR23b-UBL-F69BpA or 200 µM UBXD1-PUB was incubated with 1 mM HR23b-UBL-F69BpA. Samples were irradiated with UV light for 30 min on ice using a CL-1000 Ultraviolet Crosslinker from UVP with a 365 nm lamp (5×8 Watt).

### Chemical cross-linking analysis

Sample preparation for LC/MS/MS: Samples for LC-MS from cross-linked proteins were digested in-solution (ISD). For ISD cross-linked proteins were reduced with DTT (5 mM) in 6 M urea and 50 mM ammonium bicarbonate (ABC) for 30 min at room temperature. Protein reduction was followed by alkylation with iodoacetamide (IAM, 10 mM also in 50 mM ABC, 30 min, room temperature) and quenching of excess IAM with DTT (final concentration DTT 10 mM). Samples were then first digested with LysC for 3 h at 37 °C. After adjusting the urea concentration to 0.8 M urea the samples were digested for 16 h with trypsin at 37 °C. The digestion was stopped by adding formic acid (FA) to a final concentration of 0.5%. The supernatant containing the digestion products was passed through home-made glass microfiber StageTips (GE Healthcare; poresize: 1.2 µM; thickness: 0.26 mm). Cleared tryptic digests were then desalted on home-made C18 StageTips as described^51^. Briefly, peptides were passed over a 2 disc StageTip. After elution from the StageTips, samples were dried using a vacuum concentrator (Eppendorf) and the peptides were taken up in 0.1% FA solution (10 μL). The thus prepared samples were directly used for single play MS/MS experiments (see below for details). For double play MS/MS experiments samples were fractionated on homemade SCX StageTips as described^51^. Briefly, the analytes were immobilized on 0.1% FA pre-equilibrated SCX StageTips. Peptides were then eluted using 20 µL salt plugs with increasing ABC concentration (0, 10, 50, 100, 150, 200, 250, 300, 400, 500, 1000 mM ABC). The collected fractions were then dried in a vacuum concentrator, taken up in 10 µL 0.1% FA and directly used for LC-MS analysis.

### LC/MS/MS

Experiments were performed on an Orbitrap Elite or Orbitrap Fusion Lumos mass spectrometer (Thermo Fischer Scientific, Waltham, Massachusetts, USA) that were coupled to an EASY-nLC 1000 or 1200 liquid chromatography (LC) system (Thermo Fischer Scientific, Waltham, Massachusetts, USA). The LCs were operated in the one-column mode. The analytical column was a fused silica capillary (inner diameter 75 μm × 35–46 cm) with an integrated PicoFrit emitter (New Objective, Woburn, USA) packed in-house with Reprosil-Pur 120 C18-AQ 1.9 μm (Dr. Maisch) or Kinetex C18-XB 1.7 µm (Phenomenex). The analytical column was encased by a column oven (Sonation, Biberach an der Riß, Germany) and attached to a nanospray flex ion source (Thermo Fischer scientific, Waltham, Massachusetts, USA). The column oven temperature was adjusted to 45 °C or 50 °C during data acquisition. The LC was equipped with two mobile phases: solvent A (0.1% or 0.2% formic acid, FA, in water) and solvent B (0.1% or 0.2% FA, 20% H_2_O, in acetonitrile, ACN). All solvents were of UHPLC (ultra-high-performance liquid chromatography) grade (Honeywell, Seelze, Germany). Peptides were directly loaded onto the analytical column with a maximum flow rate that would not exceed the set pressure limit of 980 bar (usually around 0.5–0.8 µL/min). Peptide solutions (SCX fractionated or non-SCX fractionated) were subsequently separated on the analytical column using different gradients (for details see Supplementary Data 11).

The mass spectrometers were operated using Xcalibur software (Elite: v2.2 SP1.48; Lumos: v4.3.7.3.11). The mass spectrometers were set in the positive ion mode. Precursor ion scanning (MS1) was performed in the Orbitrap analyzer (FTMS; Fourier Transform Mass Spectrometry with the internal lock mass option turned on (lock mass was 445.120025 *m/z*, polysiloxane)^52^. MS2 Product ion spectra were recorded only from ions with a charge bigger than +2 and in a data dependent fashion in the FTMS. MS3 product ion spectra were only triggered when the targeted mass difference for DSSO (31.9721 Da) was detected. MS3 spectra were recorded in the IT (ion trap). All relevant MS settings (Resolution, scan range, AGC, ion acquisition time, charge states isolation window, fragmentation type and details, cycle time, number of scans performed, and various other settings) for the individual experiments can be found in Supplementary Data 12.

### Data analysis

The raw data files for DSSO cross linking experiments were directly processed in Thermo Scientific Proteome Discoverer (PD, version 2.2. or 2.4.) with the add-on node XlinkX^53^. The MS1 and MS2 spectra were extracted using the “Spectrum Selector” node with the default settings. In the “Scan Event Filter” the activation type (HCD or CID/ETD) and the MS order were adjusted depending on the experiment. In the “XlinkX Detect” node the DSSO cross linker was selected as “Crosslink Modification” (158.004 Da, Specificity STYK). From here the analysis branched off to the “XlinkX search” or a “SEQUEST” search. The following settings were used for the XlinkX search: precursor ion mass tolerance, 10 ppm; product ion mass tolerance, 20 ppm; fixed modification, Cys carbamidomethylation; variable modification, Met oxidation; allowed number of miss-cleavages, 2. All MS/MS spectra were searched against the sequences of interest concatenated to the Uniprot *E. coli* database (one protein per gene; retrieved in December 2018, containing 4308 target protein entries). FDR calculation (“XlinkX validation” node) is based on a target-Decoy approach. All peptides not considered to be cross-linked were analyzed with SEQUEST. The same database as in the XlinkX search was used. The settings are as follows: precursor ion mass tolerance, 10 ppm; fragment ion mass tolerance, 0.1 Da; fixed modification, Cys carbamidomethylation; variable modification, Met oxidation, DSSO (non cross-linked), DSSO amidated and DSSO hydrolyzed; allowed number of miss-cleavages was set to 2. The output files from XlinkX were submitted to the xiNET website for visualization^54^.

Experiments with the ubiquitin-F4BpA (Phenylalanin at position 4 replaced by the photoreactive amino acid BpA) or HR23b-UBL-F69BpA (Phenylalanin 69 replaced by BpA) were analysed using MetaMorpheus^55^. To this end, BPA was added as a custom amino acid (Name: BPA; OneLetterAbbr.: x; MonoisotopicMass: 251.09462; ChemicalFormula: C16H13NO2) and BPA setup as a crosslinker (Name: BPA; CrosslinkAminoAcid: x; CrosslinkerAminoAcid2: ACDEFGHIKLMNPQRSTVWYx; CrosslinkerTotalMass: 0). The Raw files were directly loaded into MetaMorpheus and recalibrated using the CalibrateTask. The calibrated files were then automatically taken over by the XLSearchTask. The settings for the calibration and XLSearch Task can be found the config files (see Pride submission). Experiments with photo-Met incorporation were analysed using StavroX^56^. To this end, photo-Met was setup as custom amino acid (Name: photo-Met; code: x; Composition: C6H9N3O; Mass: 139.07456). The detailed search settings and a legend for the RAW files submitted to the PRIDE repository can be found in Supplementary Data 13. The results from all searches were manually evaluated.

### Reporting summary

Further information on research design is available in the Nature Portfolio Reporting Summary linked to this article.

## Supplementary information

Supplementary Information

Description of Additional Supplementary Files (pdf)

Supplementary Data 1-14

Reporting Summary

## Data Availability

The mass spectrometry proteomics data for the cross-linking experiments of UBXD1/p97/Ub and UBXD1/HR23b generated in this study have been deposited to the ProteomeXchange Consortium via the PRIDE^57^ partner repository (https://www.ebi.ac.uk/pride/archive/) under the accession codes PXD039606 (UBXD1/p97/Ub) and PXD040984 (UBXD1/HR23b). The atomic coordinates of the UBXD1-PUB-eUBX-C model generated in this study are provided in Supplementary Data 1. The published structures used in this study are available in the Protein Data Base under the following accession codes: - ASPL:p97^22^ :pdb 5ifw [10.2210/pdb5IFW/pdb]. - UBXD1-PUB^17^ : pdb 6sap [10.2210/pdb6SAP/pdb]. - HR23b-UBL^49^ : pdb 1p1a [10.2210/pdb1P1A/pdb]. - PNGase-PUB:p97^50^ : pdb 2hpl [10.2210/pdb2HPL/pdb]. - p97 hexamer^45^ : pdb 3cf3 [10.2210/pdb3CF3/pdb]. - Ubiquitin^58^ : pdb 1d3z [10.2210/pdb1D3Z/pdb]. The AlphaFold model of UBXD1-fl used in this study is available in the AlphaFold Protein Structure Database under the accession code Q9BZV1. Source data are provided as a Source Data file. Source data are provided with this paper.

## Source data

Source Data
