# Supplementary material for: The UBX domain in UBXD1 organizes ubiquitin binding at the C-terminus of the VCP/p97 AAA-ATPase
Source: Nat Commun. 2023 Jun 5;14:3258. doi: 10.1038/s41467-023-38604-4 (PMC10241913; doi:10.1038/s41467-023-38604-4)
Supplement: Supplementary file 1 — Supplementary Information [file 41467_2023_38604_MOESM1_ESM.pdf]

The UBX domain in UBXD1 organizes ubiquitin binding at the C-terminus of the VCP/p97 AAA-ATPase

Mike Blueggel[1,†], Alexander Kroening[2,†], Matthias Kracht[2], Johannes van den Boom[2], Matthias Dabisch[1], Anna Goehring[1], Farnusch Kaschani[3], Markus Kaiser[3], Peter Bayer[1], Hemmo Meyer[2], and Christine Beuck[1*]

Supplementary Material

Supplementary Figure 1: Ubiquitin-GST / GST Western blots of UBXD1 pulldown.

Supplementary Figure 2: Chemical cross-linking of UBXD1, size exclusion chromatography of UBXD1 protease assay and AlphaFold model.

Supplementary Figure 3: Binding assays of UBXD1/p97/Ubiquitin/SUMO1 controls.

Supplementary Figure 4: ^{1}H-^{15}N-HSQC Titration of ^{15}N-Ubiquitin with UBXD1.

Supplementary Figure 5: ^{1}H-^{15}N-HSQC Titrations of UBXD1-eUBX and UBXD1-PUB with Ubiquitin, ^{1}H-^{15}N-HSQC Titration and cross-linking of UBXD1-PUB with HR23b-UBL.

Supplementary Figure 6: Synthesis of Poly-Ubiquitin chains and fluorescence anisotropy measurements of K48-linked Ubiquitin chains with UBXD1-wt protein.

Supplementary Table 1: Dissociation constants describing the affinities between various UBXD1 and p97 constructs.

Supplementary Table 2: ATPase activity assay and Michaelis Menten kinetics.

Supplementary Table 3: Oligonucleotides used as primers for PCR-amplification and for site-directed mutagenesis.

Supplementary Table 4: List of p97, UBXD1, ubiquitin and HR23 expression plasmids.

Supplementary Table 5: NMR Titration of ^{15}N-ubiquitin (Ub) with UBXD1.

Supplementary Table 6: NMR Titration of ^{15}N-ubiquitin (Ub) with UBXD1-eUBX-C.

Supplementary Table 7: NMR Titration of ^{15}N-ubiquitin with the UBX extension.

Supplementary Table 8: NMR Titration of ^{15}N-UBX extension with ubiquitin.

Supplementary Table 9: NMR Titration of ^{15}N-UBXD1-PUB with ubiquitin.

Supplementary Table 10: NMR Titration of ^{15}N-UBXD1-PUB with HR23b-UBL.

Supplementary Table 11: NMR Titration of ^{15}N- HR23b-UBL with UBXD1-PUB.

Supplementary Table 12: Distance restraints derived from crosslinks used in HADDOCK and distances after MD simulation with Yasara.

Supplementary Table 13: Distance restraints implemented to ensure proximity of the C-terminus of the PUB domain and the N-terminus of the UBX domain.

Supplementary References

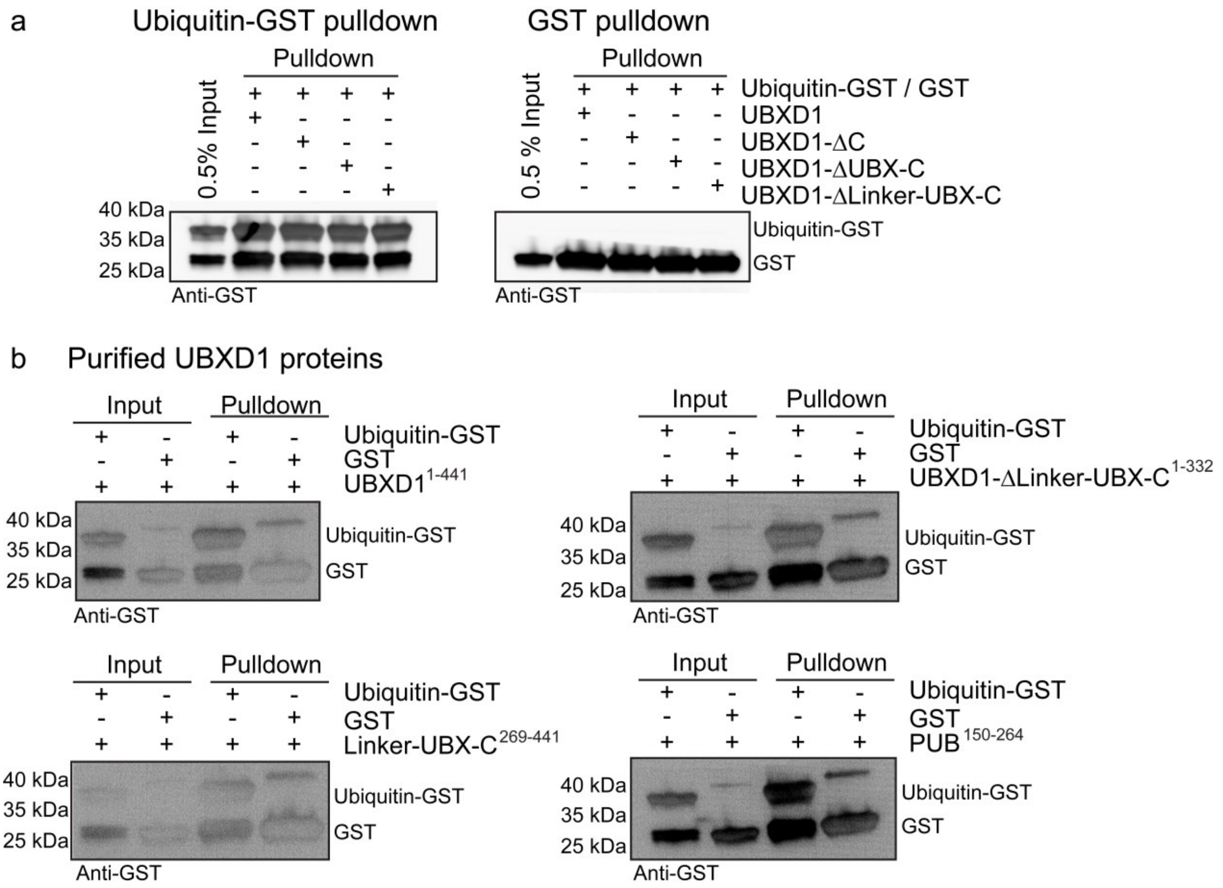

Supplementary Figure 1: *Ubiquitin-GST / GST Western blots of UBXD1 pulldown. a: Deletion of the UBX domain (333-411) abolishes Ubiquitin binding by UBXD1. Eluted Ubiquitin-GST or GST was detected with GST-specific antibody. b: Ub-GST pulldowns with purified UBXD1, UBXD1-ΔUBX-C, Linker-UBX-C or PUB domain alone. All blots (a, b) were performed as n = 1 independent experiments. Source data are provided as a source data file.*

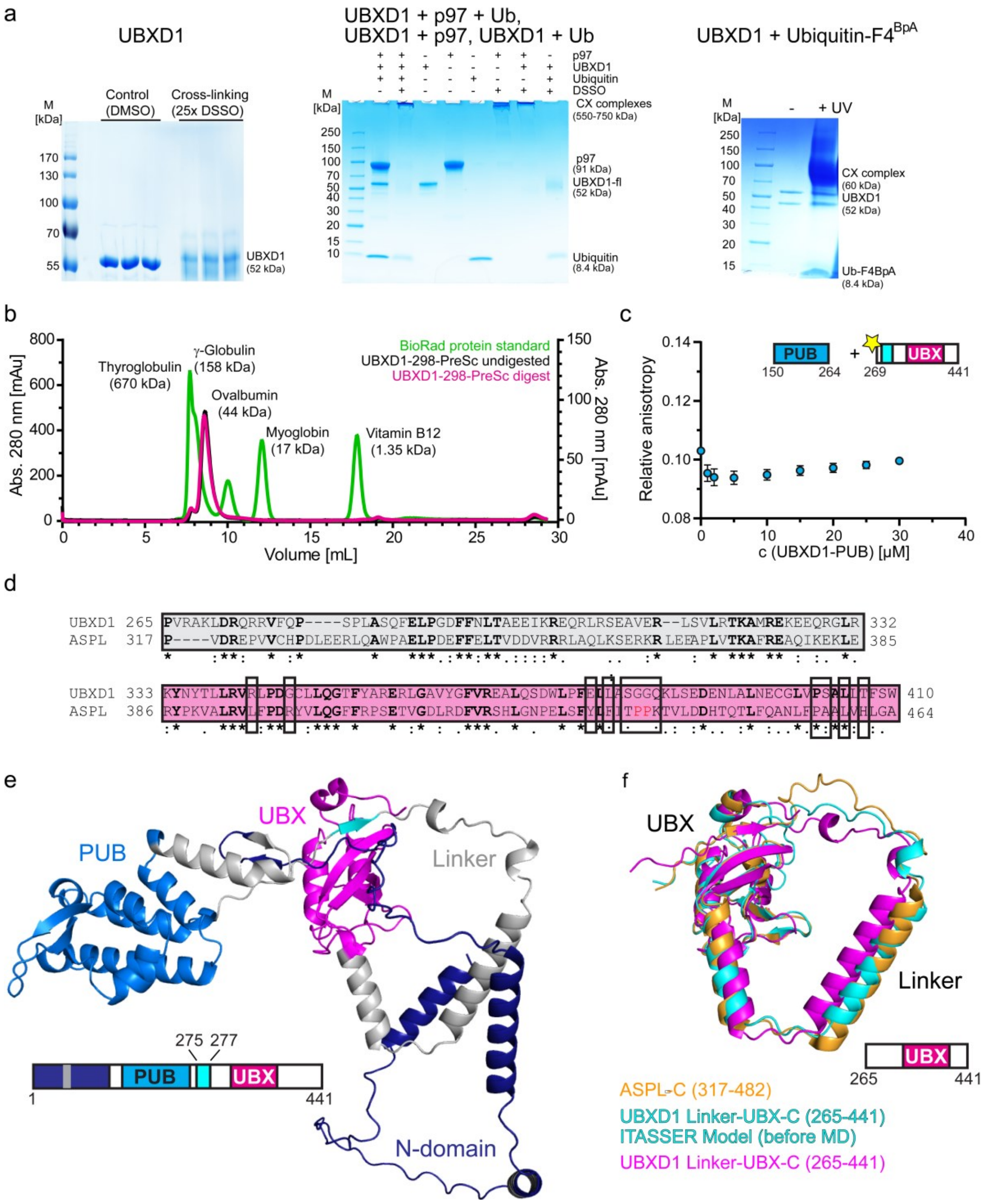

***Supplementary Figure 2:** Chemical cross-linking of UBXD1, size exclusion chromatography of UBXD1 protease assay and AlphaFold model. a: SDS gels of chemical cross-linking reactions of UBXD1 (left), UBXD1 + ubiquitin, UBXD1 + p97, UBXD1 + p97 + ubiquitin (middle) with 25x molar excess of DSSO and DMSO controls. Right: SDS gel of photo-crosslinking reaction of UBXD1 with ubiquitin-F4^{BpA}. All gels were performed as n = 1 independent experiments. b: Size exclusion chromatography of UBXD1-298-PreSc (undigest and digest + PreSc) compared to BioRad gel filtration standard (Thyroglobulin 670 kDa, γ-Globulin 158 kDa, Ovalbumin 44 kDa, Myoglobin 17 kDa and Vitamin B12 1.35 kDa), n = 1 independent experiments. c: Fluorescence anisotropy titration of Atto594-UBXD1-eUBX (containing the linker) with UBXD1 PUB shows no interaction (n = 3 independent experiments, data are represented as mean ± SD). d: Alignment of the UBXD1 and ASPL eUBX/UBX domains. * indicates conserved residues. Framed amino acids are necessary for binding of the APSL-UBX domain to the p97 N-domain. e: AlphaFold model (ID # Q9BZV1) of UBXD1-fl shows the extension of the UBX (magenta) β-sheet by the beginning region of the linker (highlighted in cyan) f: Overlay of our initial ITASSER homology model (cyan), the AlphaFold model (magenta) and the template ASPL-C Xray structure (orange, pdb: 5ifw[1]). Source data are provided as a source data file.*

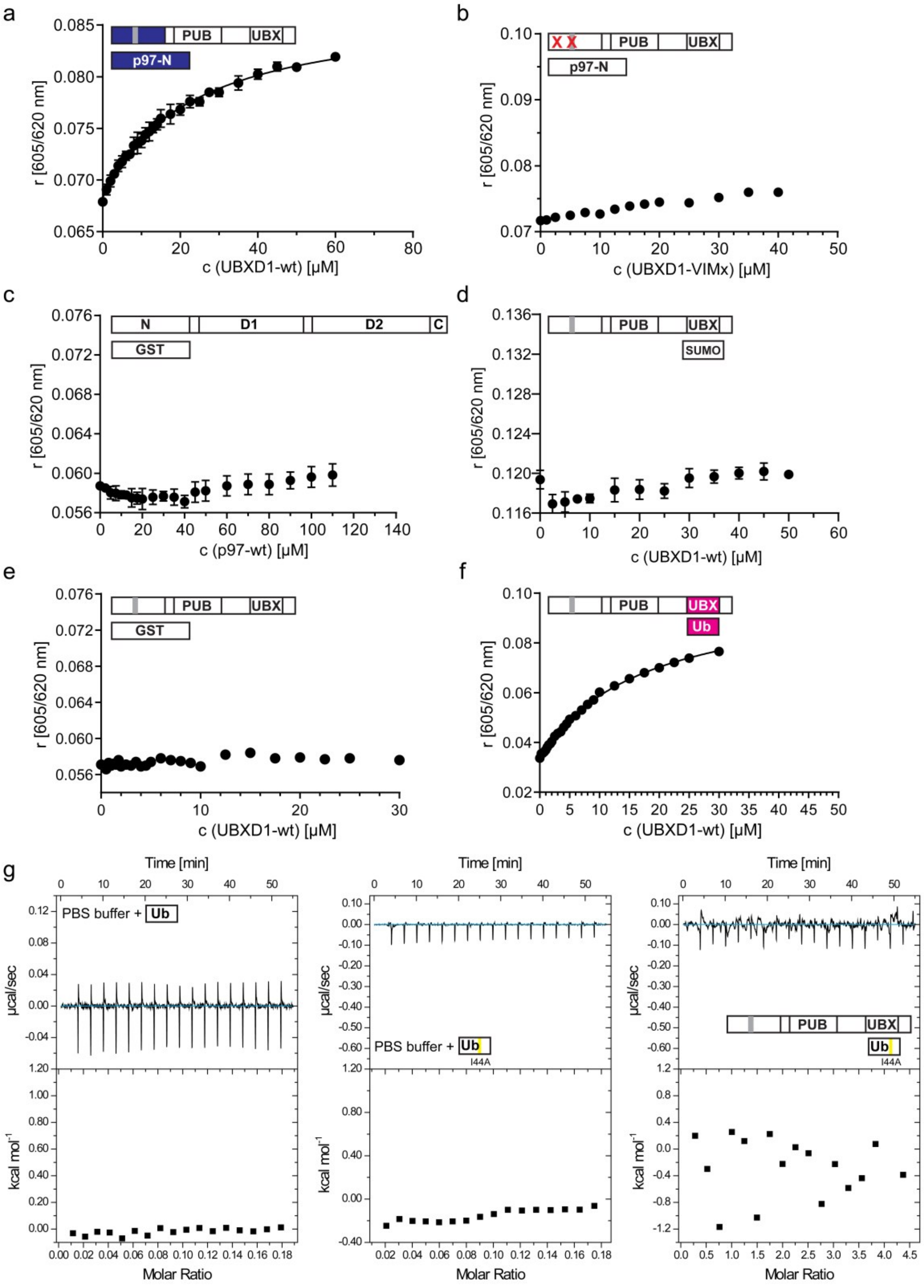

Supplementary Figure 3: *Binding assays of UBXD1/p97/Ubiquitin/SUMO1 controls. a: Fluorescence anisotropy titration of UBXD1-wt + p97-N domain (residues aa 1-199, n = 3 independent experiments. Data are represented as mean ± SD). The UBXD1-wt protein binds to the N-domain of p97 with a dissociation constant of 22 ± 1 µM. b: Fluorescence anisotropy titration of UBXD1-VIMx mutant (DI11/12AA, RL62/63AA) with the N-terminus of p97 (aa 1-199), (n = 1 independent experiments). c: Control titration of Atto594-labeled GST protein with p97-wt (n = 3 independent experiments. Data are represented as mean ± SD). d: Fluorescence anisotropy titration of Atto594-SUMO1 protein with UBXD1-wt (n = 3 independent experiments. Data are represented as mean ± SD). e: Control titration of Atto594-labeled GST protein with UBXD1-wt (n = 1 independent experiments). f: Fluorescence anisotropy titration of Atto594-Ubiquitin with UBXD1-wt (n = 1 independent experiments) shows a* K_D *of 16.9 ± 0.7 µM (*R^2 *= 0.9988). g: ITC measurements of PBS buffer with Ubiquitin-wt (left) or -I44A mutant (middle), and titration*

of Ubiquitin-I44A mutant with UBXD1 (right), (n = 1 independent experiments for all ITC measurements). Source data are provided as a source data file.

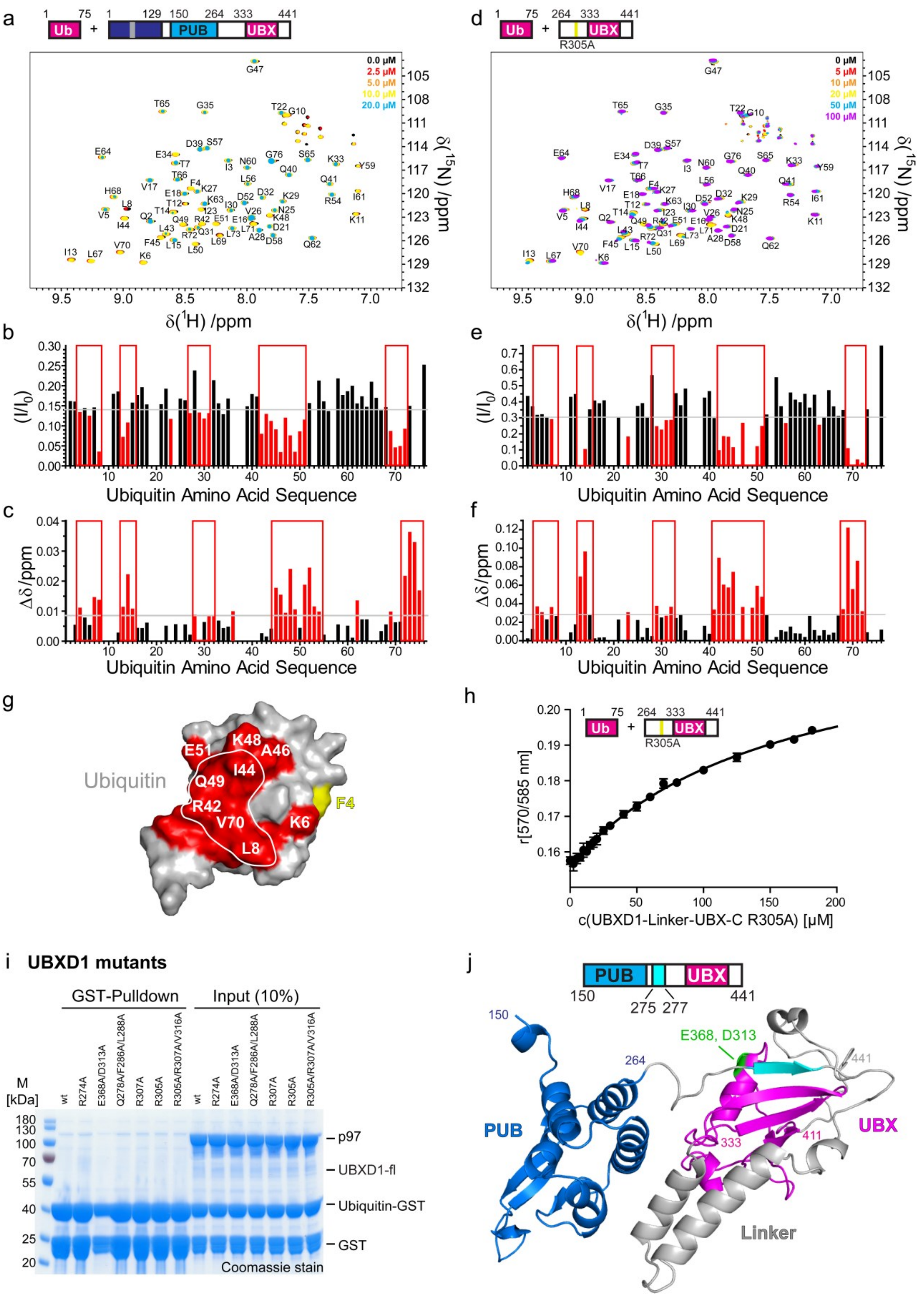

Supplementary Figure 4: *^{1}H-^{15}N-HSQC Titration of ^{15}N-Ubiquitin with UBXD1. a: 100 µM ^{15}N-Ubiquitin was titrated stepwise with UBXD1 (0, 2.5, 5.0, 10.0 and 20.0 µM). b: Histogram of relative residual signal intensity plotted against the Ubiquitin sequence (20 µM compared to 0 µM UBXD1). Grey line: mean value. Signals with lower-than-average intensity indicate specific binding and are highlighted in red. Red*

boxes mark the consensus residues from intensity and chemical shift changes. c: Histogram of chemical shift perturbation $\Delta\delta$ plotted against the Ubiquitin sequence (20 µM compared to 0 µM UBXD1). Grey line: mean value. Signals with higher-than-average shift perturbation indicate specific binding and are highlighted in red. d: 50 µM ^{15}N-Ubiquitin was titrated stepwise with UBXD1-Linker-UBX-C R305A (0, 5, 10, 25, 50 and 100 µM). e: Histogram of relative residual signal intensity plotted against the Ubiquitin sequence (100 µM compared to 0 µM UBXD1-Linker-UBX-C R305A). Grey line: mean value. Signals with lower-than-average intensity indicate specific binding and are highlighted in red. f: Histogram of chemical shift perturbation $\Delta\delta$ plotted against the Ubiquitin sequence (20 µM compared to 0 µM UBXD1). Grey line: mean value. Signals with higher-than-average shift perturbation indicate specific binding and are highlighted in red. All NMR data: n=1 independent experiments. g: Binding residues identified in B, c, e & f mapped onto the ubiquitin structure (pdb: 1d3z[2]). The classical binding area is circled by a white line. Residue F4 (colored yellow), which was exchanged for BpA in the photo-crosslinking experiment, is located at the edge of the binding interface. h: Fluorescence anisotropy titration of Rhodamine-ubiquitin with UBXD1-Linker-UBX-C R355A (residues aa 264-441) shows a K_D of 155 ± 14 µM (n = 3 independent experiments, data are represented as mean ± SD). i: Screening of UBXD1 mutants in Ub-GST pulldown with p97 (n=1 independent experiments). UBXD1 E268A/D373A shows diminished binding. j: AlphaFold model of ubiquitin (teal) bound to UBXD1-Linker-UBX-C (core UBX domain in magenta, E368 and D373 highlighted in green). Source data are provided as a source data file.

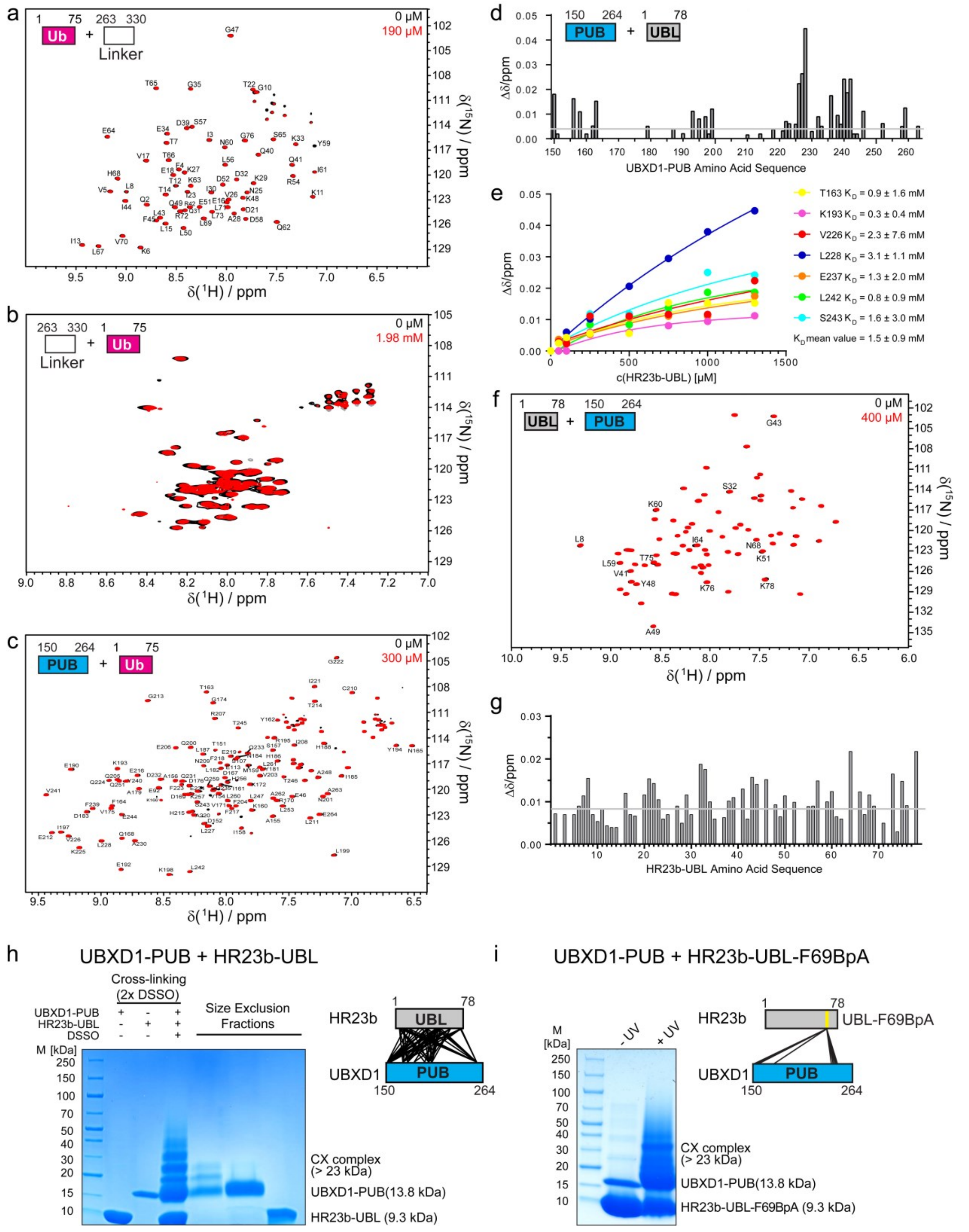

Supplementary Figure 5: *^{1}H-^{15}N-HSQC Titration of the UBXD1 extension (linker) with Ubiquitin, ^{1}H-^{15}N-HSQC Titration and cross-linking of UBXD1-PUB with HR23b-UBL. a: ^{1}H-^{15}N-HSQC Titration of ^{15}N-labeled Ubiquitin (50 µM) does not show any difference in the absence (0 µM, black) and presence (190 µM, red) of linker. b: ^{1}H-^{15}N-HSQC Titration of ^{15}N-labeled UBX extension (linker residues 263-330, 495 µM, black) does not show any effect by adding Ubiquitin up to a ratio of 1:4 (red). c: ^{1}H-^{15}N-HSQC Titration of the UBXD1-PUB domain (381 µM, black) with Ubiquitin (300 µM, red) shows no binding. d: Histogram of chemical shift perturbation plotted against the UBXD1-PUB sequence (1.3 mM compared to 0 mM HR23b-UBL). Grey line: mean value. e: Binding curves for selected residues derived from chemical shift perturbations yield an average K_D = 1.5 ± 0.9 mM. f: ^{1}H,^{15}N-HSQC titration of ^{15}N-HR23b-UBL (200 µM) with UBXD1-PUB (0 µM black, 400 µM, red). Shifting of signals indicate binding. Signals with the most pronounced chemical shift perturbation are labeled. g: Histogram of chemical shift perturbation plotted*

against the HR23b-UBL sequence (400 µM compared to 0 µM UBXD1-PUB). Grey line: mean value. All NMR data (a-g): n = 1 independent experiments. h: Chemical cross-linking of UBXD1-PUB and HR23b-UBL with DSSO after purification by SEC (n = 1 technical replicates). SDS gel of crosslinking reaction (left) and inter-molecular crosslinks (right; cross-linked amino acids see Supplementary Data 9). i: Photo-reactive cross-linking of UBXD1-PUB with HR23b-UBL-F69BpA (n = 4 technical replicates). SDS gel of crosslinking reaction (left) and inter-molecular crosslinks (right). The reactive BpA is marked by a yellow line. (cross-linked amino acids see Supplementary Data 10, FDR = 0.01). Source data are provided as a source data file.

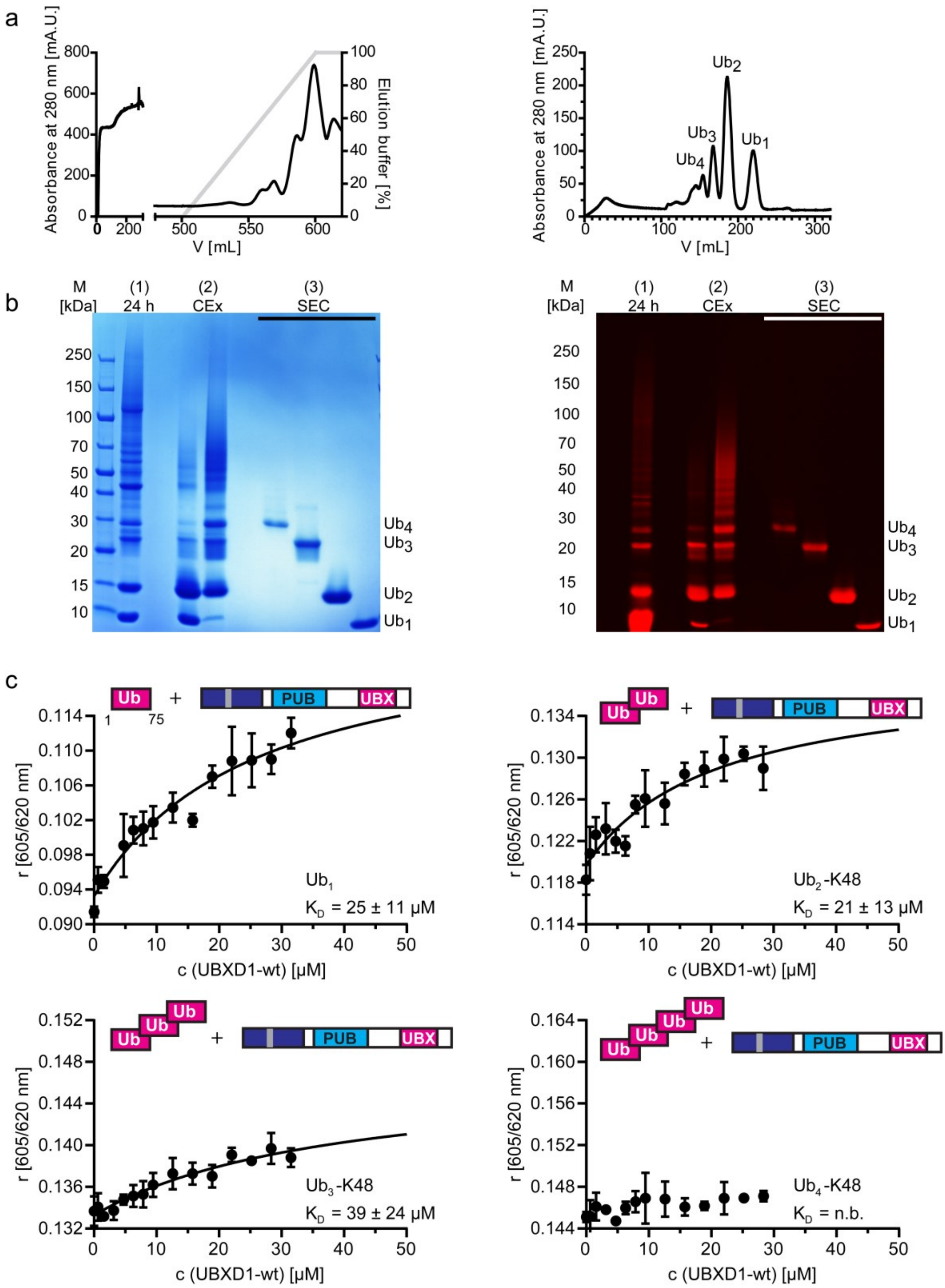

Supplementary Figure 6: *Synthesis of poly-ubiquitin chains. a: Purification of fluorescence labeled Atto594-poly-ubiquitin-M1C with strong cation exchange chromatography (left) and size exclusion chromatography (SEC, right). b: Purification was monitored by SDS-Page (n = 1 independent experiments) with Coomassie staining (left) and fluorescence imaging (right). Lane 1: molecular size standard, lane 2: reaction mixture after 24 h incubation, lane 3-4: Elution of strong cation exchange chromatography, lane 5-8: fractions of Ubiquitin chains from SEC (Ub_1: 8.4 kDa, Ub_2: 16.8 kDa, Ub_3: 25.2 kDa, Ub_4: 33.6 kDa), lane 9: molecular size standard. c: UBXD1 differentiates between Ubiquitin chains of different lengths. Fluorescence anisotropy titrations of Ubiquitin chains with UBXD1-wt protein (n = 3 independent experiments. Data are represented as mean ± SD). The dissociation constant increases with the length of Ubiquitin chains. Source data are provided as a source data file.*

Supplementary Table 1: *Dissociation constants describing the affinities between various UBXD1 and p97 constructs. All dissociation constants are calculated with the stoichiometry 1:1 for a better comparability (Replicates *n = 1, ▲n = 3, ▪displacement titration). Interacting regions in both proteins are colored (VIM helix: grey; N-terminus / N-domain: dark blue; PUB domain / C-terminus: blue; UBX extension / D2 domain: orange; UBX domain / ubiquitin: magenta). VIMx mutations (DI11/12AA, RL62/63AA) are marked with a red X. n.b. = no binding.*

UBXD1	p97	Dissociation constant K_D [μM]	Binding region (Top: UBXD1, Bottom: p97)
wild type (1-441)	wild type (1-806)	1.64 ± 0.03 μM▲ (R^2 = 0.9973)<br>1.1 ± 0.1 μM▲▪ (R^2 = 0.9870)	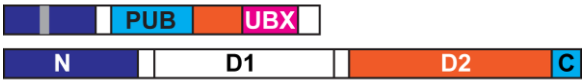
wild type (1-441)	ΔC (1-766)	11.9 ± 0.2 μM▲ (R^2 = 0.9940)	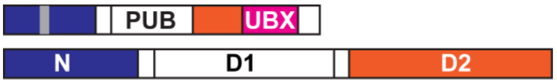
wild type (1-441)	N (1-199)	22 ± 1 μM▲ (R^2 = 0.9972)	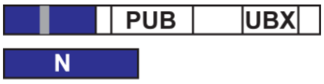
VIMx (1-441)	N (1-199)	n.b.*	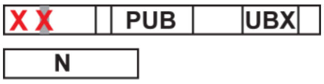
VIMx (1-441)	ΔC (1-766)	87 ± 1 μM* (R^2 = 0.9945)	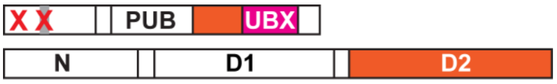
VIMx (1-441)	ΔD2-C (1-480)	466 ± 9 μM* (R^2 = 0.9697)	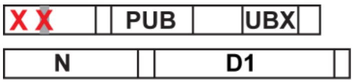
ΔUBX-C (1-332)	wild type (1-806)	5.5 ± 0.2 μM▲ (R^2 = 0.9887)	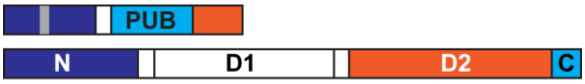
ΔUBX-C (1-332)	ΔC (1-766)	18.6 ± 0.7 μM▲ (R^2 = 0.9887)	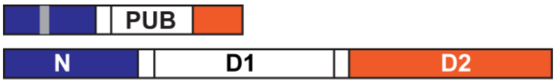
ΔUBX-C (1-332)	ΔD2-C (1-480)	26 ± 2 μM▲ (R^2 = 0.9597)	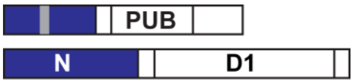
eUBX-C (269-441)	wild type (1-806)	131 ± 14 μM▲ (R^2 = 0.9976)	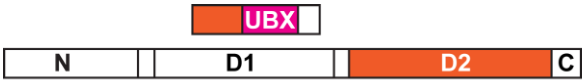
UBX extension (269-330)	wild type (1-806)	191 ± 2 μM* (R^2 = 0.9960)	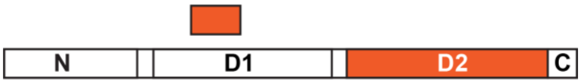
UBX extension (269-330)	ΔD2-C (1-480)	n.b.*	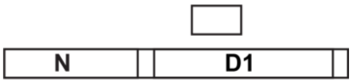

Supplementary Table 2: *ATPase activity assay and Michaelis Menten kinetics of p97 in the presence and absence of UBXD1.*

**Michaelis-Menten kinetics**		
c (ATP) [mM]	v p97 [nmol min^{-1} μg^{-1}]	v p97+UBXD1 [nmol min^{-1} μg^{-1}]
0	0 ± 0	0 ± 0
0.25	0.14 ± 0.02	0.07 ± 0.003
0.5	0.20 ± 0.01	0.10 ± 0.006
0.75	0.24 ± 0.01	0.11 ± 0.0007
1.0	0.25 ± 0.006	0.12 ± 0.014
2.0	0.26 ± 0.004	0.14 ± 0.01
3.0	0.29 ± 0.01	0.17 ± 0.005
	0 µM UBXD1	*2 µM UBXD1*
K_M [mM]	0.27 ± 0.05	0.47 ± 0.08
v_{max} [nmol Pi min^{-1} μg^{-1}]	0.31 ± 0.01	0.19 ± 0.01
R^2	0.9864	0.9855
**Lineweaver Burk fit**		
x axis intercept [$-1/K_M$]	-2.98	-2.84
y axis intercept [K_M/v_{max}]	1.0 ± 0.1	2.1 ± 0.2
R^2	0.9695	0.9750
**Calculation of the inhibition constant Ki**		
By assuming that:<br>$v_{max}^{app} = v_{max} / 1 + c (UBXD1) / K_i$<br>$K_i = v_{max}^{app} / v_{max} * (1 + c (UBXD1))$<br>$K_i$ = 0.19 nmol Pi min^{-1} μg^{-1} / 0.31 nmol Pi min^{-1} μg^{-1} * (1 + 2 µM) = 1.84 µM		
**Relative ATPase Activity of p97 with UBXD1**		
c UBXD1 [µM]	Relative ATPase Activity	
0	1 ± 0.1	
0.5	0.74 ± 0.01	
1.0	0.58 ± 0.03	
1.5	0.60 ± 0.05	
2.0	0.58 ± 0.01	
2.5	0.53 ± 0.01	
5	0.51 ± 0.02	
**Relative ATPase Activtiy**		
Protein complex	Relative ATPase Activity	
p97	1.0 ± 0.12	
p97 + UBXD1	0.60 ± 0.03	
p97 + UBXD1-VIMx	0.83 ± 0.06	
p97	1.0 ± 0.1	
p97 + CBS5083	0.51 ± 0.03	
p97 + NMS873	0.43 ± 0.12	
p97	0.99 ± 0.03	
p97 + UBXD1	0.62 ± 0.02	
p97 + Ufd1:Npl4	0.95 ± 0.05	
p97 + Ufd1:Npl4 + UBXD1	0.88 ± 0.02	

Supplementary Table 3: *Oligonucleotides used as primers for PCR-amplification of the UBXD1, ubiquitin and p97 constructs and for site-directed mutagenesis following the Quikchange protocol.*

Construct	Oligonucleotides
UBXD1 (1-332)	GGGGGCTGCGCTGATAATACAACTACACGCTG<br>CAGCGTGTAGTTGTATTATCAGCGCAGCCCCC
UBXD1 (DI11/12AA)	GGAGTTCAAGGCCGCAGCTAAGTTCAAGAGCGCGG<br>CCGCGCTCTTGAACTTAGCTGCGGCCTTGAACTCC
UBXD1 (RL62/63AA)	CTGCTGCCCTAGCCGCAGCTGAGCAGAAGCAGTCC<br>GGACTGCTTCTGCTCAGCTGCGGCTAGGGCAGCAG
UBXD1 (StrepTagII)	CTGGAAGTTCTGTTCCAGGGGCCCTGGAGCCACCCGCAGT<br>TCGAAAAGATGAAGAAATTCTTTCAGGAG<br>CTCCTGAAAGAATTTCTTCATCTTTTCGAACTGCGGGTGGC<br>TCCAGGGCCCCTGGAACAGAACTTCCAG
UBXD1-298PreSc	CTCACAGCAGAGCTGGAAGTTCTGTTCCAGGGGCCCCTGG<br>GATCCGAGATCAAGCGG<br>CCGCTTGATCTCGGATCCCAGGGGCCCCTGGAACAGAACT<br>TCCAGCTCTGCTGTGAG
p97 (1-766)	CAGCAGAGTCGGTGATAATTTGGCAGCTTCAGATTCC<br>GGAATCTGAAGCTGCCAAATTATCACCGACTCTGCTG
p97 (1-480)	GGAAGACATCGGGTGACTAGAGGATGTC<br>GACATCCTCTAGTCACCCGATGTCTTCC
Ubiquitin (M1C)	GGAGCTAGCCATATGTGCCAGATCTTCGTGAAG<br>CTTCACGAAGATCTGGCACATATGGCTAGCTCC
Ubiquitin (F4BpA)	GCCATATGCAGATCTAGGTGAAGACCCTGACC<br>GGTCAGGGTCTTCACCTAGATCTGCATATGGC

Supplementary Table 4: *List of p97- UBXD1-, ubiquitin and HR23 expression plasmids. For coding DNA sequences see Supplementary Data 14.*

**Construct**	**Plasmid**	**Tag**	**Protease site**	**Experiments**
**p97**				
His6-p97-fl	pET15b	N-term His6	Thrombin	Anisotropy, Pulldown, Crosslinking, ATPase activity assay, Unfolding Assay
His6-p97-ΔD2-C	pET15b	N-term His6	PreScission	Anisotropy, Pulldown
His6-p97-ΔC	pET15b	N-term His6	PreScission	Anisotropy
His6-p97-N	pET28a	N-term His6	TEV	Anisotropy
**UBXD1**				
GST-UBXD1-fl	pGEX6P1	N-term GST	PreScission	GST-Pulldown, Anisotropy, NMR, Crosslinking, ITC, ATPase activity assay, Unfolding Assay
GST-UBXD1-ΔLinker-UBX-C	pGEX6P1	N-term GST	PreScission	Pulldown
GST-UBXD1-fl-VIMx	pGEX6P1	N-term GST	PreScission	Anisotropy
GST-UBXD1-ΔUBX-C	pGEX6P1	N-term GST	PreScission	Anisotropy
GST-UBXD1-UBX extension / -Linker	pET41b	N-term GST	PreScission	Anisotropy
GST-UBXD1-UBX extension / -Linker R305A	pET41b	N-term GST	PreScission	Anisotropy, NMR
His6-UBXD1-PUB	pET28a	N-term His6	Thrombin	Pulldown, Anisotropy, NMR, Crosslinking
GST-UBXD1-Linker-UBX-C / -eUBX-C	pGEX6P1	N-term GST	PreScission	Pulldown, Anisotropy
UBXD1-298PreSc-His8	*pET41b*	C-term His8	Aa298 PreScission, C-term none	SEC, Crosslinking
StrepTagII-UBXD1-fl	pcDNA3.1(+)	N-term StrepTagII	none	Strep-Pulldown
StrepTagII-UBXD1-ΔC	pcDNA3.1(+)	N-term StrepTagII	none	Strep-Pulldown
StrepTagII-UBXD1-ΔUBX-C	pcDNA3.1(+)	N-term StrepTagII	none	Strep-Pulldown
StrepTagII-UBXD1-ΔLinker-UBX-C	pcDNA3.1(+)	N-term StrepTagII	none	Strep-Pulldown
StrepTagII control vector	pcDNA3.1(+)	N-term StrepTagII	none	Strep-Pulldown
GST-UBXD1-His wt	pGEX-6P-1	N-term GST, C-term His6	PreScission	GST-pulldown with Ubiquitin-GST, unfolding assay
GST-UBXD1-TwinStrep wt	pGEX-6P-1	N-term GST, C-term His6	PreScission	Strep-pulldown with p97 and Ufd1-Npl4
GST-UBXD1-His R305A	pGEX-6P-1	N-term GST, C-term His6	PreScission	screening-pulldown with Ubiquitin-GST
GST-UBXD1-His R307A	pGEX-6P-1	N-term GST, C-term His6	PreScission	screening -pulldown with Ubiquitin-GST
GST-UBXD1-His R305A R307A V316A	pGEX-6P-1	N-term GST, C-term His6	PreScission	screening-pulldown with Ubiquitin-GST
GST-UBXD1-His R274A	pGEX-6P-1	N-term GST, C-term His6	PreScission	screening-pulldown with Ubiquitin-GST
GST-UBXD1-His Q278A F286A L288A	pGEX-6P-1	N-term GST, C-term His6	PreScission	screening-pulldown with Ubiquitin-GST
GST-UBXD1-His E328A D373A	pGEX-6P-1	N-term GST, C-term His6	PreScission	GST-pulldown with Ubiquitin-GST
GST-UBXD1-His Δ 265-441	pGEX-6P-1	N-term GST, C-term His6	PreScission	GST-pulldown with Ubiquitin-GST
**Ubiquitin & SUMO**				
Ubiquitin-GST	pET23a	C-term GST	none	GST-Pulldown
Ubiquitin	pET23a	none	none	Crosslinking, Anisotropy, ITC
Ubiquitin-M1C	pET23a	none	none	Anisotropy
Ubiquitin-F4BpA	pET23a	none	none	Crosslinking
Ubiquitin-I44A	pET23a	none	none	ITC
GST-SUMO1	pGEX2T	N-term GST	Thrombin	Anisotropy
**HR23b**				
GST-HR23b-UBL	pGEX6P1	N-term GST	PreScission	NMR, Crosslinking
GST-HR23b-UBL-F69BpA	pGEX6P1	N-term GST	PreScission	Crosslinking

Supplementary Table 5: *NMR Titration of ^{15}N-ubiquitin (Ub) with UBXD1.*

Spectrum	c (^{15}N-Ub) [µM]	c (UBXD1) [µM]	Ratio (UBXD1:Ub)
1	100.0	0.0	0.00
2	97.8	2.5	0.03
3	95.7	5.0	0.05
4	91.4	10.0	0.11
5	82.7	20.0	0.24

Supplementary Table 6: *NMR Titration of ^{15}N-ubiquitin (Ub) with UBXD1-eUBX-C.*

Spectrum	c (^{15}N-Ub) [µM]	c (UBXD1-eUBX-C) [µM]	Ratio (UBXD1-eUBX-C:Ub)
1	50.0	0	0
2	49.4	5	0.1
3	48.7	10	0.2
4	47.5	20	0.4
5	43.7	50	1.0
6	37.3	100	2.0

Supplementary Table 7: *NMR Titration of ^{15}N-ubiquitin with the UBX extension.*

Spectrum	c (^{15}N-Ub) [µM]	c (UBXD1-UBX extension) [µM]	Ratio (UBXD1-UBX extension:Ub)
1	50.0	0	0
2	49.4	5	0.1
3	48.7	10	0.2
4	47.5	20	0.4
5	45.6	35	0.7
6	43.7	50	1.0
7	40.5	75	1.5
8	37.3	100	2.0
9	31.0	150	3.0
10	25.8	190	3.8

Supplementary Table 8: *NMR Titration of ^{15}N-UBX extension with ubiquitin.*

Spectrum	c (^{15}N-UBXD1-UBX extension) [µM]	c (Ub) [µM]	Ratio (Ub:UBXD1-UBX extension)
1	495.0	0	0
2	489.3	50	0.1
3	483.6	100	0.2
4	478.0	150	0.3
5	472.3	200	0.4
6	460.9	300	0.6
7	449.6	400	0.8
8	438.2	500	1.0
9	381.5	1000	2.0

10	270.1	1981	4.0

Supplementary Table 9: *NMR Titration of ^{15}N-UBXD1-PUB with ubiquitin.*

Spectrum	c (^{15}N-UBXD1-UBX extension) [µM]	c (Ub) [µM]	Ratio (Ub:UBXD1-UBX extension)
1	381.0	0	0
2	380.1	10	0.03
3	378.8	25	0.07
4	376.6	50	0.13
5	372.3	100	0.26
6	367.9	150	0.39
7	363.5	200	0.52
8	354.8	300	10.79

Supplementary Table 10: *NMR Titration of ^{15}N-UBXD1-PUB with HR23b-UBL.*

Spectrum	c(^{15}N-UBXD1-PUB) [µM]	c(HR23b-UBL) [µM]	Ratio (HR23b-UBL:UBXD1-PUB)
1	400.0	0	0.0
2	395.1	50	0.1
3	390.1	100	0.3
4	375.3	250	0.7
5	350.6	500	1.4
6	325.9	750	2.3
7	301.2	1000	3.3
8	271.6	1300	4.8

Supplementary Table 11: *NMR Titration of ^{15}N- HR23b-UBL with UBXD1-PUB.*

Spectrum	c(^{15}N-HR23b-UBL) [µM]	c(UBXD1-PUB) [µM]	Ratio (UBXD1-PUB:HR23b-UBL)
1	200.0	0	0.0
2	185.6	50	0.3
3	171.2	100	0.6
4	142.4	200	1.4
5	128.1	250	2.0
6	113.7	300	2.6
7	84.9	400	4.7

Supplementary Table 12: *Distance restraints derived from crosslinks used in HADDOCK and distances after MD simulation with Yasara.*

Crosslinked Residues	Distance Restraint	Distance after MD
166-333	0-30 Å CA-CA	17.686 Å
166-269	0-30 Å CA-CA	19.426 Å
166-325	0-30 Å CA-CA	17.479 Å
172-333	0-30 Å CA-CA	11.167 Å
257-269	0-30 Å CA-CA	12.874 Å
163-320	0-30 Å CA-CA	27.117 Å
163-325	0-30 Å CA-CA	20.601 Å
166-320	0-30 Å CA-CA	25.049 Å
166-334	0-30 Å CA-CA	20.437 Å
172-325	0-30 Å CA-CA	11.637 Å
177-325	0-30 Å CA-CA	8.248 Å
180-325	0-30 Å CA-CA	11.814 Å
193-325	0-30 Å CA-CA	24.101 Å
194-325	0-30 Å CA-CA	22.518 Å
194-333	0-30 Å CA-CA	33.075 Å
198-269	0-30 Å CA-CA	31.649 Å
202-325	0-30 Å CA-CA	15.484 Å
202-333	0-30 Å CA-CA	29.615 Å
198-269	0-30 Å CA-CA	31.649 Å
202-325	0-30 Å CA-CA	15.484 Å
202-333	0-30 Å CA-CA	29.615 Å
256-333	0-30 Å CA-CA	26.446 Å
257-320	0-30 Å CA-CA	23.478 Å
257-325	0-30 Å CA-CA	19.719 Å
257-333	0-30 Å CA-CA	23.118 Å

Supplementary Table 13: *Distance restraints implemented to ensure proximity of the C-terminus of the PUB domain and the N-terminus of the UBX domain.*

Restrained Residues	Distance Restraint	Distance after MD
263-265	0-10 Å CA-CA	7.166 Å
264-266	0-10 Å CA-CA	6.076 Å
264-265	0-5 Å CA-C	4.724 Å
264-265	0-5 Å C-C	3.190 Å
264-265	0-5 Å for C-CA	2.485 Å
264-265	0-5 Å N-C	5.500 Å
264-265	0-5 Å C-N	1.338 Å

Supplementary References

1. Arumughan, A. *et al.* Quantitative interaction mapping reveals an extended UBX domain in ASPL that disrupts functional p97 hexamers. *Nature communications* **7,** 13047; 10.1038/ncomms13047 (2016).

2. Cornilescu, G., Marquardt, J.L., Ottiger, M. & Bax, A. Validation of Protein Structure from Anisotropic Carbonyl Chemical Shifts in a Dilute Liquid Crystalline Phase. *J. Am. Chem. Soc.* **120**, 6836-6837; 10.1021/ja9812610 (1998).
