# Supplementary material for: The UBX domain in UBXD1 organizes ubiquitin binding at the C-terminus of the VCP/p97 AAA-ATPase
Source: Nat Commun. 2023 Jun 5;14:3258. doi: 10.1038/s41467-023-38604-4 (PMC10241913; doi:10.1038/s41467-023-38604-4)
Supplement: Supplementary file 2 — Description of Additional Supplementary Files (pdf) [file 41467_2023_38604_MOESM2_ESM.pdf]

The UBX domain in UBXD1 organizes ubiquitin binding at the C-terminus of the VCP/p97 AAA-ATPase

Mike Blueggel[1,†], Alexander Kroening[2,†], Matthias Kracht[2], Johannes van den Boom[2], Matthias Dabisch[1], Anna Goehring[1], Farnusch Kaschani[3], Markus Kaiser[3], Peter Bayer[1], Hemmo Meyer[2], and Christine Beuck[1*]

Description of Additional Supplementary Files

File Name: Supplementary Data 1
Description: Atomic coordinates of the UBXD1-PUB-eUBX-C structural model (pdb format)

File Name: Supplementary Data 2
Description: Identified intramolecular chemical cross-link results from UBXD1 with DSSO in four biological replicates.

File Name: Supplementary Data 3
Description: Identified intermolecular chemical cross-link results from UBXD1-298PreSc with DSSO in three technical replicates.

File Name: Supplementary Data 4
Description: Identified intermolecular chemical cross-links between UBXD1 and Ubiquitin with DSSO in three technical replicates.

File Name: Supplementary Data 5
Description: Identified intermolecular photo-reactive cross-links between UBXD1 and Ubiquitin-F4BpA(p-Benzoyl-L-phenylalanine)

File Name: Supplementary Data 6
Description: Identified intermolecular chemical cross-link results from UBXD1 and p97 with DSSO in three technical replicates.

File Name: Supplementary Data 7
Description:: Identified intermolecular chemical cross-link results from UBXD1 and p97-photoLeu/photoMet in three technical replicates.

File Name: Supplementary Data 8
Description: Identified intermolecular chemical cross-link results between UBXD1, p97 and Ubiquitin present in one sample after SEC purification.

File Name: Supplementary Data 9
Description: Identified intermolecular and UBXD1 intramolecular chemical cross-link results from UBXD1-PUB and HR23b-UBL with DSSO present in one sample after SEC purification.

File Name: Supplementary Data 10
Description: Identified intermolecular photo-reactive cross-links between UBXD1-PUB and HR23b-UBL-F69BpA present in four technical replicates.

File Name: Supplementary Data 11
Description: LC Settings for LC-MS.

File Name: Supplementary Data 12
Description: MS Settings.

File Name: Supplementary Data 13
Description: Legends and XLinkX settings.

File Name: Supplementary Data 14
Description: DNA sequences of coding DNA sequences for all protein constructs.
