# Supplementary material for: The UBX domain in UBXD1 organizes ubiquitin binding at the C-terminus of the VCP/p97 AAA-ATPase
Source: Nat Commun. 2023 Jun 5;14:3258. doi: 10.1038/s41467-023-38604-4 (PMC10241913; doi:10.1038/s41467-023-38604-4)
Supplement: Supplementary file 3 — Supplementary Data 1-14 [file 41467_2023_38604_MOESM3_ESM.zip › Supplementary_Data_11_LC-Settings-for-LC-MS.pdf]

Supplementary Data 11: *LC Settings for LC-MS.*

ACE_0353_MB04

MS device	Thermo Orbitrap Elite
LC device	Thermo Easy-nLC 1000
ion source	Thermo Nanospray Flex
Analytical column	Self-packed fused silica capillary with integrated pico frit emitter; New Objectives PF360-75-15-N-5
column diameter	Length (L_C) = 22 cm; ID = 75µm; OD = 360 µm; emitter 15 µm
stationary phase	Reprosil-Pur 120 C18-AQ, Dr. Maisch GmbH
particle diameter (d_p)	1.9 µm
Pore size	120 Å
Column ID	AC59
Column oven	Sonation column oven PRSO-V1
Column oven temp.	45°C
solvents	A: 0.1% FA in UPLC water<br>B: 0.1% FA in UPLC ACN
gradient	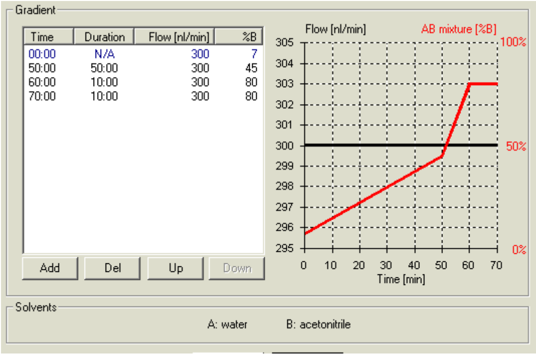

MS device	Thermo Orbitrap Elite
LC device	Thermo Easy-nLC 1000
ion source	Thermo Nanospray Flex
Analytical column	Self-packed fused silica capillary with integrated pico frit emitter; New Objectives PF360-75-15-N-5
column diameter	Length (L_C) = 35 cm; ID = 75µm; OD = 360 µm; emitter 15 µm
stationary phase	Reprosil-Pur 120 C18-AQ, Dr. Maisch GmbH
particle diameter (d_p)	1.9 µm
Pore size	120 Å
Column ID	AC60
Column oven	Sonation column oven PRSO-V1
Column oven temp.	45°C
solvents	A: 0.1% FA in UPLC water<br>B: 0.1% FA in UPLC ACN
gradient	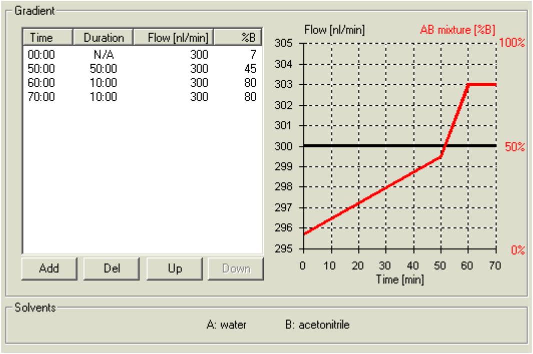

ACE_0372_MB16/18

MS device	Thermo Orbitrap Elite
LC device	Thermo Easy-nLC 1000
ion source	Thermo Nanospray Flex
Analytical column	Self-packed fused silica capillary with integrated pico frit emitter; New Objectives PF360-75-15-N-5
column diameter	Length (L_C) = 40 cm; ID = 75µm; OD = 360 µm; emitter 15 µm
stationary phase	Reprosil-Pur 120 C18-AQ, Dr. Maisch GmbH
particle diameter (d_p)	1.9 µm
Pore size	120 Å
Column ID	AC64
Column oven	Sonation column oven PRSO-V1
Column oven temp.	50°C
solvents	A: 0.1% FA in UPLC water<br>B: 0.1% FA in UPLC ACN
gradient	

ACE_0393_MB01/02

MS device	Thermo Orbitrap Elite
LC device	Thermo Easy-nLC 1000
ion source	Thermo Nanospray Flex
Analytical column	Self-packed fused silica capillary with integrated pico frit emitter; New Objectives PF360-75-15-N-5
column diameter	Length (L_C) = 40 cm; ID = 75µm; OD = 360 µm; emitter 15 µm
stationary phase	Reprosil-Pur 120 C18-AQ, Dr. Maisch GmbH
particle diameter (d_p)	1.9 µm
Pore size	120 Å
Column ID	AC64
Column oven	Sonation column oven PRSO-V1
Column oven temp.	45°C
solvents	A: 0.1% FA in UPLC water<br>B: 0.1% FA in UPLC ACN
gradient	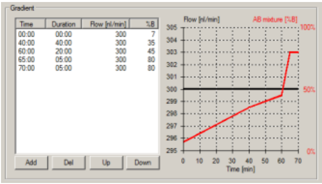

MS device	Thermo Orbitrap Fusion LUMOS
LC device	Thermo Easy-nLC 1000
ion source	Thermo Nanospray Flex
Analytical column	Self-packed fused silica capillary with integrated pico frit emitter; New Objectives PF360-75-15-N-5
column diameter	Length (L_C) = 50 cm; ID = 75µm; OD = 360 µm; emitter 15 µm
stationary phase	Reprosil-Pur 120 C18-AQ, Dr. Maisch GmbH
particle diameter (d_p)	1.9 µm
Pore size	120 Å
Column ID	AC73
Column oven	Sonation column oven PRSO-V2
Column oven temp.	50°C
solvents	A: 0.1% FA in UPLC water<br>B: 0.1% FA, 20% H_2O in UPLC ACN
gradient	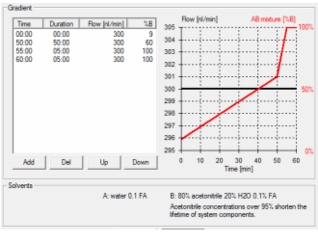

MS device	Therno Orbitrap Fusion LUMOS
LC device	Thermo Easy-nLC 1200
ion source	Thermo Nanospray Flex
Analytical column	Self-packed fused silica capillary with integrated pico frit emitter; New Objectives PF360-75-15-N-5
column diameter	Length (L_C) = 50 cm; ID = 75µm; OD = 360 µm; emitter 15 µm
stationary phase	Reprosil-Pur 120 C18-AQ, Dr. Maisch GmbH
particle diameter (d_p)	1.9 µm
Pore size	120 Å
Column ID	AC73
Column oven	Sonation column oven PRSO-V2
Column oven temp.	50°C
solvents	A: 0.1% FA in UPLC water<br>B: 0.1% FA, 20% H_2O in UPLC ACN
gradient	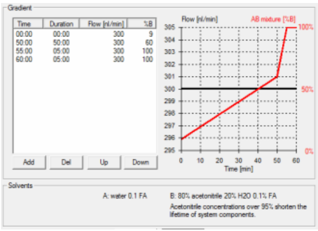

MS device	Orbitrap Fusion Lumos
LC device	Thermo Easy-nLC 1200
ion source	Thermo Nanospray Flex
Analytical column	Self-packed fused silica capillary with integrated emitter (no frit); ESi Source Solutions PTC3-75-50-SP
column diameter	Length (L_C) = 44 cm; ID = 75µm; OD = 360 µm; emitter 5 µm
stationary phase	Reprosil-Pur 120 C18-AQ, Dr. Maisch GmbH
particle diameter (d_p)	1.9 µm
Pore size	120 Å
Column ID	AC98
Column oven	Sonation column oven PRSO-V2
Column oven temp.	50°C
solvents	A: 0.1% FA in UPLC water<br>B: 0.1% FA, 20% H_2O in UPLC ACN
gradient	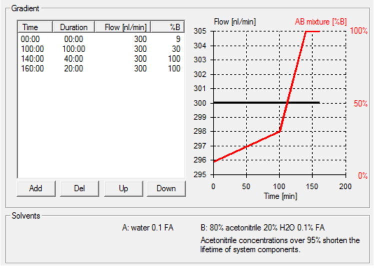

<table>
<tr><td>MS device</td><td>Orbitrap Fusion Lumos</td></tr>
<tr><td>LC device</td><td>Thermo Easy-nLC 1200</td></tr>
<tr><td>ion source</td><td>Thermo Nanospray Flex</td></tr>
<tr><td>Analytical column</td><td>Self-packed fused silica capillary with an integrated sintered frit; CoAnn Technologies ICT36007515F-50-5</td></tr>
<tr><td>column diameter</td><td>Length (LC) = 41 cm; ID = 75µm; OD = 360 µm; emitter 15 µm</td></tr>
<tr><td>stationary phase</td><td>Phenomenex Kinetex C18-XB core shell</td></tr>
<tr><td>particle diameter (dp)</td><td>1.7 µm (core shell)</td></tr>
<tr><td>Pore size</td><td>100 Å</td></tr>
<tr><td>Column ID</td><td>AC127</td></tr>
<tr><td>Column oven</td><td>Sonation column oven PRSO-V2</td></tr>
<tr><td>Column oven temp.</td><td>50°C</td></tr>
<tr><td>solvents</td><td>A: 0.2% FA in UPLC water
B: 0.2% FA, 20% H2O, 80% ACN</td></tr>
<tr><td>gradient</td><td>
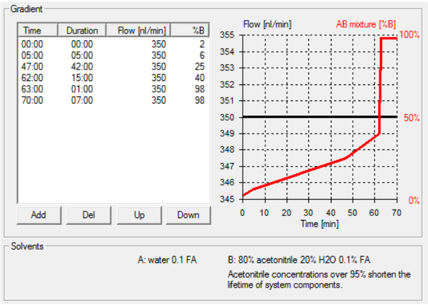

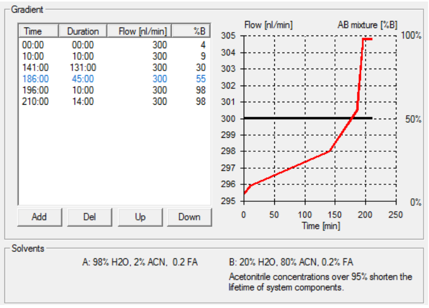

</td></tr>
</table>

MS device	Thermo Orbitrap Fusion Lumos
LC device	Thermo Easy-nLC 1200
ion source	Thermo Nanospray Flex
Analytical column	Self-packed fused silica capillary with integrated pico frit emitter; CoAnn Tech. ICT36007515F-50-5
column diameter	Length (L_C) = 42 cm (AC107) and 46 cm (AC106); ID = 75µm; OD = 360 µm; emitter 15 µm
stationary phase	AC107: Reprosil-Pur 120 C18-AQ, Dr. Maisch GmbH<br><br>AC106 : Reprosil Fluosil 120 PFP, Dr. Maisch GmbH
particle diameter (d_p)	1.9 µm (AC107); 2.2 (AC106)
Pore size	120 Å
Column ID	AC107; AC106
Column oven	Sonation column oven PRSO-V2
Column oven temp.	50°C
**solvents**	A: 0.1% FA in UPLC water<br><br>B: 0.1% FA in UPLC ACN
gradient	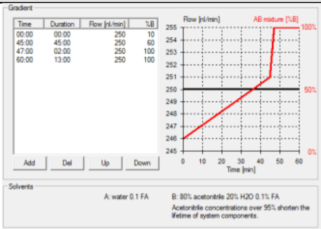<br><br>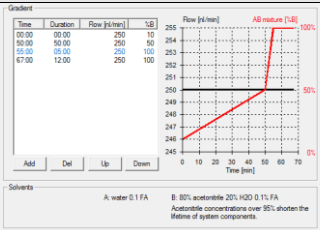

<table>
<tr><td>MS device</td><td>Thermo Orbitrap Fusion Lumos</td></tr>
<tr><td>LC device</td><td>Thermo Easy-nLC 1200</td></tr>
<tr><td>ion source</td><td>Thermo Nanospray Flex</td></tr>
<tr><td>Analytical column</td><td>Self-packed fused silica capillary with integrated pico frit emitter; CoAnn Tech. ICT36007515F-50-5</td></tr>
<tr><td>column diameter</td><td>Length (L_C) = 42 cm; ID = 75µm; OD = 360 µm; emitter 15 µm</td></tr>
<tr><td>stationary phase</td><td>Reprosil-Pur 120 C18-AQ, Dr. Maisch GmbH</td></tr>
<tr><td>particle diameter (d_p)</td><td>1.9 µm</td></tr>
<tr><td>Pore size</td><td>120 Å</td></tr>
<tr><td>Column ID</td><td>AC107</td></tr>
<tr><td>Column oven</td><td>Sonation column oven PRSO-V2</td></tr>
<tr><td>Column oven temp.</td><td>50°C</td></tr>
<tr><td>solvents</td><td>A: 0.1% FA in UPLC water

B: 0.1% FA in UPLC ACN</td></tr>
<tr><td>gradient</td><td>
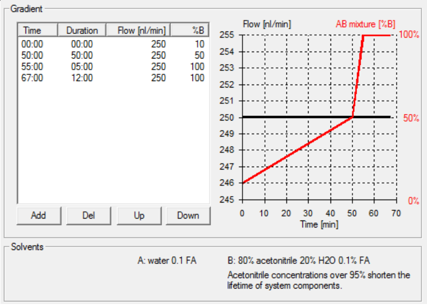

</td></tr>
</table>
