# Supplementary material for: The UBX domain in UBXD1 organizes ubiquitin binding at the C-terminus of the VCP/p97 AAA-ATPase
Source: Nat Commun. 2023 Jun 5;14:3258. doi: 10.1038/s41467-023-38604-4 (PMC10241913; doi:10.1038/s41467-023-38604-4)
Supplement: Supplementary file 3 — Supplementary Data 1-14 [file 41467_2023_38604_MOESM3_ESM.zip › Supplementary_Data_12_MS-Settings-for-LC-MS.pdf]

***Supplementary Data 12**: MS Settings.*

Project	MS	general	MS1	MS2	MS2	MS3	Comments; special settings
ACE_0353<br>ACE_0371	Elite	TunePlus v2.7.0 SP1<br>Gradient: 70 min	Analyzer: FT<br>Res.: 60000<br>SR: 350 - 1500<br>AGC: 1×10^6<br>AcT: 50<br>DDM: NS	Analyzer: FT<br>Res.: 15000<br>SR: variable<br>AGC: 1×10^5<br>AcT: 120 ms<br>CS: >+2<br>Frag.: CID<br>NCE: 35<br>NS: 5×	Analyzer: FT<br>Res.: 15000<br>SR: variable<br>AGC: 1×10^5<br>AcT: 120 ms<br>CS: >+2<br>Frag.: ETD<br>NCE: n/a<br>NS: 5×		5th order double play. MS2 was done in OT. Each mass selected for fragmentation was first fragmented by HCD and then by ETD. MS2's were only performed on charge states >+2.
ACE_0372-01	Elite	TunePlus v2.7.0 SP1<br>Gradient: 140 min	Analyzer: FT<br>Res.: 120000<br>SR: 300 - 2000<br>AGC: 1×10^6<br>AcT: 70<br>DDM: NS	Analyzer: FT<br>Res.: 15000<br>SR: variable<br>AGC: 5×10^5<br>AcT: 250 ms<br>CS: >+2<br>Frag.: HCD<br>NCE: 31<br>NS: 10×			MS2 was done in OT; MS2 was performed only on charge states >+2.
ACE_0372-02	Elite	TunePlus v2.7.0 SP1<br>Gradient: 140 min	Analyzer: FT<br>Res.: 60000<br>SR: 350 - 2000<br>AGC: 1×10^6<br>AcT: 70<br>DDM: NS	Analyzer: FT<br>Res.: 15000<br>SR: variable<br>AGC: 5×10^5<br>AcT: 250 ms<br>CS: >+2<br>Frag.: CID<br>NCE: 35<br>NS: 5×	Analyzer: FT<br>Res.: 15000<br>SR: variable<br>AGC: 5×10^5<br>AcT: 250 ms<br>CS: >+2<br>Frag.: ETD<br>NCE: n/a<br>NS: 5×		5th order double play. MS2 was done in OT. Each mass selected for fragmentation was first fragmented by HCD and then by ETD. MS2's were only performed on charge states >+2.
ACE_0393	Elite	TunePlus v2.7.0 SP1<br>Gradient: 70 min	Analyzer: FT<br>Res.: 120000<br>SR: 300 - 2000<br>AGC: 1×10^6<br>AcT: 70<br>DDM: NS	Analyzer: FT<br>Res.: 15000<br>SR: variable<br>AGC: 5×10^5<br>AcT: 250 ms<br>CS: >+2<br>Frag.: HCD<br>NCE: 31<br>NS: 10×			MS2 was done in OT; MS2 was performed only on charge states >+2.

ACE_0422<br>MS2-MS2-MS3	Lumos	Tune v3.1.2412.25<br>Gradient: 60 min	Analyzer: FT<br>Res.: 120000<br>SR: 375 - 1600<br>AGC: standard<br>AcT: 50<br>RF: 30<br>SF: 10V<br>DDM: CT/5sec	Analyzer: FT<br>Res.: 30000<br>SR: Auto<br>AGC: Auto<br>AcT: 100 ms<br>CS: >+2<br>IsM: Q<br>IsW: 1.6 (MS)<br>Frag.: CID<br>NCE: 25	Analyzer: FT<br>Res.: 50000<br>SR: Auto<br>AGC: 400%<br>AcT: 150 ms<br>CS: >+2<br>IsM: Q<br>IsW: 1.6 (MS)<br>Frag.: EThcD<br>NCE: 20<br>NS: 1	Analyzer: IT<br>Res.: 15000<br>SR: Auto<br>AGC: 200%<br>AcT: 120 ms<br>CS: >+2<br>IsM: IT<br>IsW: 2.5 (MS) 2 (MS2)<br>Frag.: HCD<br>NCE: 35<br>NS: 4	First MS2 is triggered by targeted mass difference 31.9721 (DSSO). Second MS2 is performed in OT with EThcD. Fragments from first MS2 with triggered mass difference were collected for MS3 experiments (HCD in IT). The whole sequence MS1-MS2-MS2/MS3 was repeated for 5 sec (cycle time between master scans).
ACE_0422<br>ACE_0425<br>MS2-stepped HCD	Lumos	Tune v3.1.2412.25<br>Gradient: 60 min	Analyzer: FT<br>Res.: 120000<br>SR: 375 - 1600<br>AGC: Standard<br>AcT: 50<br>RF: 30<br>SF: 10V<br>DDM: CT/5sec	Analyzer: FT<br>Res.: 30000<br>SR: Auto<br>AGC: Standard<br>AcT: 150 ms<br>CS: >+2<br>IsM: Q<br>IsW: 1.6 (MS)<br>Frag.: sHCD<br>NCE: 21,27,33			MS2 is performed on all ions with charge >+2. Fragmentation type is stepped HCD (sHCD). Each 1/3 of AGC will be separately treated with predefined increasing NCE (here 21, 27, 33). The ions are collected in the IRM. Once all portions were fragmented the whole batch is send to the OT for analysis at the same time. Advantage of this approach … at low NCE the cross linker (DSSO) cleaves … and releases the signature peptides .. at higher fragmentation these arms will be fragmented. So we have diagnostic peaks in the spectrum (which can be nicely used to identify CL spectra) and fragments from the arms for identification.
ACE_0556	Lumos	Tune v v3.3.2782.28<br>Gradient: 160 min	Analyzer: FT<br>Res.: 120000<br>SR: 400 - 1600<br>AGC: 150%<br>AcT: 60<br>RF: 30<br>SF: --<br>DDM: CT/5sec	Analyzer: FT<br>Res.: 30000<br>SR: Auto<br>AGC: 150%<br>AcT: 200 ms<br>CS: >+2<br>IsM: Q<br>IsW: 1.2 (MS)<br>Frag.: sHCD<br>NCE: 27,30,33			MS2 is performed on all ions with charge >+2. Fragmentation type is stepped HCD (sHCD). Each 1/3 of AGC will be separately treated with predefined increasing NCE (here 27, 30, 33). The ions are collected in the IRM. Once all portions were fragmented the whole batch is send to the OT for analysis at the same time. Advantage of this approach … at low NCE the cross linker (DSSO) cleaves … and releases the signature peptides .. at higher fragmentation these arms will be fragmented. So we have diagnostic peaks in the spectrum (which can be nicely used to identify CL spectra) and fragments from the arms for identification.

ACE_0716	Lumos	Tune v3.3.2782.28<br>Gradient: 70 & 105 min	Analyzer: FT<br>Res.: 120000<br>SR: 375 - 1600<br>AGC: Standard<br>AcT: Auto<br>RF: 30<br>SF: --<br>DDM: CT/5sec	Analyzer: FT<br>Res./ScR: 15000/-<br>SR: Auto<br>AGC: Standard<br>AcT: 200ms<br>CS: +3 to +7<br>IsM: Q<br>IsW: 2<br>Frag.: sHCD<br>NCE: 27, 30, 33			MS2 is performed on all ions with charge >+2. Fragmentation type is stepped HCD (sHCD). Each 1/3 of AGC will be separately treated with predefined increasing NCE (here 27, 30, 33). The ions are collected in the IRM. Once all portions were fragmented the whole batch is send to the OT for analysis at the same time. Advantage of this approach … at low NCE the cross linker (DSSO) cleaves … and releases the signature peptides .. at higher fragmentation these arms will be fragmented. So we have diagnostic peaks in the spectrum (which can be nicely used to identify CL spectra) and fragments from the arms for identification.
ACE_0630<br>MS2-MS2-MS3	Lumos	Tune v3.1.2412.25<br>Gradient: 60 min	Analyzer: FT<br>Res.: 120000<br>SR: 375 - 1600<br>AGC: standard<br>AcT: 50<br>RF: 30<br>SF: 10V<br>DDM: CT/5sec	Analyzer: FT<br>Res.: 30000<br>SR: Auto<br>AGC: Auto<br>AcT: 100 ms<br>CS: >+2<br>IsM: Q<br>IsW: 1.6 (MS)<br>Frag.: CID<br>NCE: 25	Analyzer: FT<br>Res.: 50000<br>SR: Auto<br>AGC: 400%<br>AcT: 150 ms<br>CS: >+2<br>IsM: Q<br>IsW: 1.6 (MS)<br>Frag.: sHCD<br>NCE: 15, 30,50<br>NS: 1	Analyzer: IT<br>Res.: 15000<br>SR: Auto<br>AGC: 200%<br>AcT: 120 ms<br>CS: >+2<br>IsM: IT<br>IsW: 2.5 (MS) 2 (MS2)<br>Frag.: HCD<br>NCE: 35<br>NS: 4	First MS2 is triggered by targeted mass difference 31.9721 (DSSO). Second MS2 is performed in OT with sHCD. Fragments from first MS2 with triggered mass difference were collected for MS3 experiments (HCD in IT). The whole sequence MS1-MS2-MS2/MS3 was repeated for 5 sec (cycle time between master scans).
ACE_0631<br>MS2	Lumos	Tune v v3.3.2782.28<br>Gradient: 67 min	Analyzer: FT<br>Res.: 60000<br>SR: 400 - 1400<br>AGC: 150%<br>AcT: auto<br>RF: 30<br>SF: --<br>DDM: CT/3sec	Analyzer: FT<br>Res.: 30000<br>SR: Auto<br>AGC: 300%<br>AcT: 100 ms<br>CS: >+2<br>IsM: Q<br>IsW: 1.6 (MS)<br>Frag.: aHCD<br>NCE: 25, 30			MS2 is performed on all ions with charge >+2. Fragmentation type is assisted HCD (aHCD). The MS selects the optimal NCE from provided list (here 25, 30).<br>Intensity Threshold: 50000<br>Dynamic exclusion: 30 sec; ±10 ppm

Note: **FT**= Fourier Transform (Orbitrap); **IT**= Iontrap; **Q**= Quadrupol; **Res.**= max. Resolution at 200 m/z (Lumos) or 400 m/z (Elite) [FWHM (full width at half maximum)]; **SR**= scan range [m/z]; **AGC**= automatic gain control, max number of acquired ions per measurement; **AcT**= max. Ion acquisition time [ms]; **CS**= charge states used for fragmentation; **IsM**= Isolation mode (Q or IT), MS2 isolation and further is only done in IT; **IsW**= Isolation window [m/z], value followed by scan mode the isolation is based on (MS1, MS2 …) **Frag.**= Fragmentation method; **HCD**= Higher-energy collisional dissociation; **CID**= Collision-induced dissociation; **ETD**= Electron-transfer dissociation**; EThcD=** Electron-Transfer/Higher-Energy Collision Dissociation; **sHCD**= stepped HCD**; NCE**= normalized collision energy; **cycles**: number of MSn recorded or max cycle time; RF= RF Lens [%]; **SF**= Source Fragmentation [V]; **DDM**: Data dependent Mode (cycle time in seconds, CT/[s] or number of scans, NS); **NS**= Number of data dependent scans
