# Supplementary material for: The UBX domain in UBXD1 organizes ubiquitin binding at the C-terminus of the VCP/p97 AAA-ATPase
Source: Nat Commun. 2023 Jun 5;14:3258. doi: 10.1038/s41467-023-38604-4 (PMC10241913; doi:10.1038/s41467-023-38604-4)
Supplement: Supplementary file 3 — Supplementary Data 1-14 [file 41467_2023_38604_MOESM3_ESM.zip › Supplementary_Data_13_Legends-XLinkX-settings.pdf]

Supplementary Table S13: Legends and XLinkX settings.

01_UBXD1

ACE ID	Organism	Organ/ cell line	Treatment/ experimental setup	Search	XlinkX settings
ACE_0353_MB04_C1.raw<br>ACE_0353_MB04_C2.raw<br>ACE_0353_MB04_C3.raw<br>ACE_0353_MB04_C4.raw<br>ACE_0353_MB04_C5.raw<br>ACE_0353_MB04_C6.raw<br>ACE_0353_MB04_C7.raw<br>ACE_0353_MB04_C8.raw<br>ACE_0353_MB04_C9.raw<br>ACE_0353_MB04_C10.raw<br><br>10 SCX fractions	human	Recomb. protein	UBXD1<br>250 µg purified UBXD1 (overexpressed in E. coli) was proceed with DSSO, reaction mixture was separate by size-exclusion chromatography	XlinkX<br>Elite	Search strategy: MS2_MS2<br>Crosslink mod: DSSO_K<br>Precursor mass: 10 ppm<br>FTMS Fragment Mass: 20 ppm<br>ITMS Fragment Mass: 0.5 Da<br>FDR: 0.01<br>min score: 20
ACE_0371_MB10_C1.raw<br>ACE_0371_MB10_C2.raw<br>ACE_0371_MB10_C3.raw<br>ACE_0371_MB10_C4.raw<br>ACE_0371_MB10_C5.raw<br>ACE_0371_MB10_C6.raw<br>ACE_0371_MB10_C7.raw<br>ACE_0371_MB10_C8.raw<br>ACE_0371_MB10_C9.raw<br>ACE_0371_MB10_C10.raw<br>ACE_0371_MB10_C11.raw<br><br>11 SCX fractions	human	Recomb. protein	UBXD1-CX control<br>250 µg purified UBXD1 (overexpressed in E. coli) was proceed with DSSO alone as control	XlinkX<br>Elite	Search strategy: MS2_MS2<br>Crosslink mod: DSSO_KSTY<br>Precursor mass: 10 ppm<br>FTMS Fragment Mass: 20 ppm<br>ITMS Fragment Mass: 0.5 Da<br>FDR: 0.01<br>min score: 20
ACE_0372_MB18.raw	human	Recomb.<br>protein	UBXD1<br>250 µg purified UBXD1 (overexpressed in E. coli) was proceed with DSSO	XlinkX<br>Elite	Search strategy: MS2_MS2<br>Crosslink mod: DSSO_KSTY<br>Precursor mass: 10 ppm<br>FTMS Fragment Mass: 20 ppm<br>ITMS Fragment Mass: 0.5 Da<br>FDR: 0.01

					min score: 20
ACE_0425_MB13_MS2_MS3.raw	human	Recomb.<br>protein	UBXD1<br>52 µg purified UBXD1 was proceed with 2.5x DSSO	XlinkX<br>Lumos	Search strategy: MS2_MS3<br>Crosslink mod: DSSO_KSTY<br>Precursor mass: 10 ppm<br>FTMS Fragment Mass: 20 ppm<br>ITMS Fragment Mass: 0.6 Da<br>FDR: 0.01<br>min score: 20
ACE_0425_MB15_MS2_MS3.raw	human	Recomb.<br>protein	UBXD1<br>52 µg purified UBXD1 was proceed with 2.5x DSSO	XlinkX<br>Lumos	dito

02_UBXD1-298-PreSc

ACE ID	Organism	Organ/ cell line	Treatment/ experimental setup		XlinkX settings
ACE_0422_MB01_MS2_MS3.raw<br>ACE_0422_MB01_MS2_stepped.raw	human	Recomb. protein	UBXD1-298PreSc-Fragments<br>69 µg purified UBXD1 (overexpressed in E. coli) was proceed with DSSO	XlinkX<br>Lumos	Search strategy: MS2 or MS2_MS3<br>Crosslink mod: DSSO_KSTY<br>Precursor mass: 10 ppm<br>FTMS Fragment Mass: 20 ppm<br>ITMS Fragment Mass: 0.6 Da<br>FDR: 0.01<br>min score: 20
ACE_0422_MB02_MS2_MS3.raw<br>ACE_0422_MB02_MS2_MS3_20190926043716.raw<br>ACE_0422_MB02_MS2_stepped.raw	human	Recomb. protein	UBXD1-298PreSc-Fragments<br>69 µg purified UBXD1 (overexpressed in E. coli) was proceed with DSSO	XlinkX<br>Lumos	dito
ACE_0422_MB03_MS2_MS3.raw<br>ACE_0422_MB03_MS2_stepped.raw	human	Recomb. protein	UBXD1-298PreSc-Fragments<br>69 µg purified UBXD1 (overexpressed in E. coli) was proceed with DSSO	XlinkX<br>Lumos	dito

03_UBXD1 + Ubiquitin

ACE ID	Organism	Organ/ cell line	Treatment/ experimental setup	Search	XlinkX settings
ACE_0372_MB16.raw<br>ACE_0372_MB16_02.raw	human	Recomb.<br>protein	UBXD1/Ub<br>250 µg purified UBXD1 (overexpressed in E. coli) was proceed with 105 µg Ubiquitin and DSSO	XlinkX<br>Elite	Search strategy: MS2_MS2<br>Crosslink mod: DSSO_KSTY<br>Precursor mass: 10 ppm<br>FTMS Fragment Mass: 20 ppm<br>ITMS Fragment Mass: 0.5 Da<br>FDR: 0.01<br>min score: 20
ACE_0425_MB07_MS2_MS3.raw	human	Recomb.<br>protein	UBXD1/Ubiquitin<br>52 µg purified UBXD1 + 21 µg Ubiquitin was proceed with 2.5x DSSO	XlinkX<br>Lumos	Search strategy: MS2_MS3<br>Crosslink mod: DSSO_KSTY<br>Precursor mass: 10 ppm<br>FTMS Fragment Mass: 20 ppm<br>ITMS Fragment Mass: 0.6 Da<br>FDR: 0.01<br>min score: 20
ACE_0425_MB08_MS2_MS3.raw	human	Recomb.<br>protein	UBXD1/Ubiquitin<br>52 µg purified UBXD1 + 21 µg Ubiquitin was proceed with 2.5x DSSO	XlinkX<br>Lumos	dito
ACE_0425_MB09_MS2_MS3.raw	human	Recomb.<br>protein	UBXD1/Ubiquitin<br>52 µg purified UBXD1 + 21 µg Ubiquitin was proceed with 2.5x DSSO	XlinkX<br>Lumos	dito

04_UBXD1 + Ubiquitin-F4BpA

ACE ID	Organism	Organ/ cell line	Treatment/ experimental setup		MetaMorpheus 0.0.316. settings
ACE_0556-01_MB01.raw	Human	Recomb. protein	10 µM StrepTagII-UBXD1 + 50 µM Ubiquitin-F4BPA (5x Ub-F4BPA)	MM<br>Lumos	Search strategy: MS2<br>Crosslink mod: Bpa<br>Precursor mass: 10 ppm<br>Fragment Mass: 20 ppm<br>FDR: 0.01<br>min score: 2
ACE_0556-01_MB02.raw	Human	Recomb. protein	10 µM StrepTagII-UBXD1 + 125 µM Ubiquitin-F4BPA (12.5x Ub-F4BPA)	MM<br>Lumos	dito
ACE_0556-01_MB03.raw	Human	Recomb. protein	10 µM StrepTagII-UBXD1 + 250 µM Ubiquitin-F4BPA (25x Ub-F4BPA)	MM<br>Lumos	dito
ACE_0556-01_MB04.raw	Human	Recomb. protein	50 µM StrepTagII-UBXD1 + 2.5 mM Ubiquitin-F4BPA (50x Ub-F4BPA)	MM<br>Lumos	dito

05_UBXD1 + p97

ACE ID	Organism	Organ/ cell line	Treatment/ experimental setup	Search	XlinkX settings
ACE_0425_MB01_MS2_MS3.raw	human	Recomb. protein	UBXD1/p97<br>52 µg purified UBXD1 + 230 µg p97 was proceed with 2.5x DSSO	XlinkX<br>Lumos	Search strategy: MS2_MS3<br>Crosslink mod: DSSO_KSTY<br>Precursor mass: 10 ppm<br>FTMS Fragment Mass: 20 ppm<br>ITMS Fragment Mass: 0.6 Da<br>FDR: 0.01<br>min score: 20
ACE_0425_MB02_MS2_MS3.raw	human	Recomb.<br>protein	UBXD1/p97<br>52 µg purified UBXD1 + 230 µg p97 was proceed with 2.5x DSSO	XlinkX<br>Lumos	dito

06_UBXD1 + p97+pLeu/pMet

ACE ID	Organism	Organ/ cell line	Treatment/ experimental setup	Search	StavroX 3.6.6.6. settings
ACE_0393_MB01	human	Recomb. protein	p97 + UBXD1<br>184 µg purified p97 (overexpressed in E. coli and tagged with photo-Leu and photo-Met) + 260 µg UBXD1	StavroX<br>Elite	Search strategy: MS2<br>Crosslink mod: photo-Met<br>Precursor mass: 10 ppm<br>Fragment Mass: 10 ppm<br>FDR: 0.05<br>min score: data dependent

07_UBXD1 + p97 + Ub

ACE ID	Organism	Organ/ cell line	Treatment/ experimental setup	Search	XlinkX settings
ACE_0716_CB01	human	Recomb. protein	10 µM UBXD1 + 25 µM p97 + 50 µM Ubiquitin + 25× DSSO<br>Purified via SEC (Superose 6) in B#_A. Fraction #26.	XlinkX<br>Lumos	Search strategy: MS2<br>Crosslink mod: DSSO_KSTY<br>Precursor mass: 10 ppm<br>FTMS Fragment Mass: 20 ppm<br>ITMS Fragment Mass: 0.6 Da<br>FDR: 0.01<br>min score: 20
ACE_0716_CB02	human	Recomb. protein	10 µM UBXD1 + 25 µM p97 + 50 µM Ubiquitin + 25× DSSO<br>Purified via SEC (Superose 6) in B#_A. Fraction #23.	XlinkX<br>Lumos	*dito*
ACE_0716_CB03	human	Recomb. protein	10 µM UBXD1 + 25 µM p97 + 50 µM Ubiquitin + 25× DSSO<br>Purified via SEC (Superose 6) in B#_A. Fraction #24.	XlinkX<br>Lumos	*dito*

08_UBXD1 + HR23b

ACE ID	Organism	Organ/ cell line	Treatment/ experimental setup	Search	XlinkX settings
ACE_0630_MB01<br><br>ACE_0630_MB01_C18	human	Recomb. protein	50 µM UBXD1-PUB + 500 µM hHR23b-UBL + 1.1 mM DSSO<br><br>34.5 µg UBXD1-PUB, 269.5 µg hHR23b-UBL	XlinkX<br><br>Lumos	Search strategy: MS2<br><br>Crosslink mod: DSSO_KSTY<br><br>Precursor mass: 10 ppm<br><br>FTMS Fragment Mass: 20 ppm<br><br>ITMS Fragment Mass: 0.6 Da<br><br>FDR: 0.01<br><br>min score: 20
ACE_0630_MB02<br><br>ACE_0630_MB02_C18	human	Recomb. protein	50 µM UBXD1-PUB + 500 µM hHR23b-UBL + 1.1 mM DSSO<br><br>34.5 µg UBXD1-PUB, 269.5 µg hHR23b-UBL	XlinkX<br><br>Lumos	*dito*
ACE_0630_MB03<br><br>ACE_0630_MB03-C18	human	Recomb. protein	50 µM UBXD1-PUB + 500 µM hHR23b-UBL + 1.1 mM DSSO<br><br>34.5 µg UBXD1-PUB, 269.5 µg hHR23b-UBL	XlinkX<br><br>Lumos	*dito*
ACE_0630_MB07<br><br>ACE_0630_MB07_C18	human	Recomb. protein	UBXD1-PUB-hHR23-UBL cross-linked complex, SEC fraction 17	XlinkX<br><br>Lumos	*dito*
ACE_0630_MB08<br><br>ACE_0630_MB08_C18	human	Recomb. protein	UBXD1-PUB-hHR23-UBL cross-linked complex, SEC fraction 17	XlinkX<br><br>Lumos	*dito*
ACE_0630_MB09<br><br>ACE_0630_MB09_C18	human	Recomb. protein	UBXD1-PUB-hHR23-UBL cross-linked complex, SEC fraction 17	XlinkX<br><br>Lumos	*dito*

09_UBXD1 + HR23b-UBL-F69BpA

ACE ID	Organism	Organ/ cell line	Treatment/ experimental setup	Search	XlinkX settings
ACE_0631_MB01	human	Recomb. protein	50 µM UBXD1-PUB domain + 250 µM HR23b-UBL-F69BpA domain<br><br>13.8 µg UBXD1-PUB + 49 µg HR23b-UBL	MM<br><br>Lumos	Search strategy: MS2<br><br>Crosslink mod: Bpa<br><br>Precursor mass: 10 ppm<br><br>Fragment Mass: 20 ppm<br><br>FDR: 0.01<br><br>min score: 2
ACE_0631_MB02	human	Recomb. protein	50 µM UBXD1-PUB domain + 500 µM HR23b-UBL-F69BpA domain<br><br>13.8 µg UBXD1-PUB + 98 µg HR23b-UBL	MM<br><br>Lumos	*dito*
ACE_0631_MB03	human	Recomb. protein	50 µM UBXD1-PUB domain + 750 µM HR23b-UBL-F69BpA domain<br><br>13.8 µg UBXD1-PUB + 147 µg HR23b-UBL	MM<br><br>Lumos	*dito*
ACE_0631_MB04	human	Recomb. protein	50 µM UBXD1-PUB domain + 1 mM HR23b-UBL-F69BpA domain<br><br>13.8 µg UBXD1-PUB + 196 µg HR23b-UBL	MM<br><br>Lumos	*dito*
ACE_0631_MB05	human	Recomb. protein	200 µM UBXD1-PUB domain + 1 mM HR23b-UBL-F69BpA domain<br><br>55.2 µg UBXD1-PUB + 196 µg HR23b-UBL	MM<br><br>Lumos	*dito*
