# Supplementary material for: The UBX domain in UBXD1 organizes ubiquitin binding at the C-terminus of the VCP/p97 AAA-ATPase
Source: Nat Commun. 2023 Jun 5;14:3258. doi: 10.1038/s41467-023-38604-4 (PMC10241913; doi:10.1038/s41467-023-38604-4)
Supplement: Supplementary file 4 — Reporting Summary [file 41467_2023_38604_MOESM4_ESM.pdf]

nature portfolio

Corresponding author(s): Christine Beuck

Last updated by author(s): Apr 20, 2023

Reporting Summary

Nature Portfolio wishes to improve the reproducibility of the work that we publish. This form provides structure for consistency and transparency in reporting. For further information on Nature Portfolio policies, see our Editorial Policies and the Editorial Policy Checklist.

Statistics

For all statistical analyses, confirm that the following items are present in the figure legend, table legend, main text, or Methods section.

n/a	Confirmed	
☐	☒	The exact sample size (*n*) for each experimental group/condition, given as a discrete number and unit of measurement
☐	☒	A statement on whether measurements were taken from distinct samples or whether the same sample was measured repeatedly
☐	☒	The statistical test(s) used AND whether they are one- or two-sided<br>*Only common tests should be described solely by name; describe more complex techniques in the Methods section.*
☒	☐	A description of all covariates tested
☒	☐	A description of any assumptions or corrections, such as tests of normality and adjustment for multiple comparisons
☐	☒	A full description of the statistical parameters including central tendency (e.g. means) or other basic estimates (e.g. regression coefficient) AND variation (e.g. standard deviation) or associated estimates of uncertainty (e.g. confidence intervals)
☐	☒	For null hypothesis testing, the test statistic (e.g. *F*, *t*, *r*) with confidence intervals, effect sizes, degrees of freedom and *P* value noted<br>*Give P values as exact values whenever suitable.*
☒	☐	For Bayesian analysis, information on the choice of priors and Markov chain Monte Carlo settings
☒	☐	For hierarchical and complex designs, identification of the appropriate level for tests and full reporting of outcomes
☒	☐	Estimates of effect sizes (e.g. Cohen's *d*, Pearson's *r*), indicating how they were calculated

Our web collection on statistics for biologists contains articles on many of the points above.

Software and code

Policy information about availability of computer code

Data collection	-ITC experiments: MicroCal iTC200 software (version 1.26.1) from GE Healthcare/ Malvern Panalytical supplied with the instrument.<br>- Fluorescence anisotropy measurements: Software Spectra Manager™ II from Jasco supplied with the instrument.<br>- Fluorescence unfolding assay: Varian Cary Eclipse (Kinetics Application) v1.1 supplied with the instrument.<br>- Colorimetric ATPase assay: Varian Cary winUV v3.00 supplied with the instrument.<br>- NMR experiments: Topspin 3.7 (Bruker) with the NMRlib 2.0 pulse sequence tools library from IBS (Grenoble, France) available at http://www.ibs.fr/research/scientific-output/software/pulse-sequence-tools/ .<br>- Chromatography: BioRad ChromLab 6.0 supplied with the instrument.<br>- Homology Modelling: I-TASSER server (https://zhanggroup.org/I-TASSER/).<br>- Docking: Haddock 2.4 server (https://www.bonvinlab.org/software/haddock2.4/).<br>- MD simulations: Yasara 21.8.27 (available at http://www.yasara.org/).<br>- Western Blot Imaging: intras ECL ChemoStar TS 0.5.83.0 supplied with the intrument.<br>- Mass Spectrometry: Thermo Xcalibur (Orbitrap Elite v3.0.63 & Orbitrap Fusion Lumos v4.3.73.11), Thermo TunePlus (Elite v2.7.0 SP1) & Tune (Lunos v3.1.2412.25) (all provided with instruments).
Data analysis	- ITC experiments: ITC thermograms were fitted to a one set of sites model with the software MicroCal Analysis Launcher in Origin provided with the instrument (Origin 7; version 7.0552). Heat of dilution was subtracted as constant from each data point.<br>- Fluorescence anisotropy measurements: Data was plotted and fitted to a one-site binding model with GraphPad Prism 5.0.<br>- Fluorescence unfolding assay: Data was plotted with GraphPad Prism 5.0.<br>- Colorimetric ATPase assay data were plotted and fitted with GraphPad Prism 5.0.<br>- NMR experiments: Spectra were processed with Topspin 3.7 (Bruker) and analyzed in CARA (version 1.9.1.7; http://cara.nmr.ch). Chemical

shift perturbation and relative signal intensities were calculated from the raw chemical shift data and peak intensities using Excel 2016 (Microsoft) and plotted with GraphPad Prism 5.0.
- Chromatograms were evaluated with BioRad ChromLab 6.0 (provided with the instrument) and plotted in GraphPad Prism 5.0.
- Structural models after homology modelling, docking & MD simulations were visualized with PyMol 1.3.
- Mass Spectrometry: Thermo Proteome Discoverer versions 2.2 and 2.4. & XlinkX 2.0 (commercially available from Thermo Scientific, PD Reader for free); MetaMorpheus 0.0.320 (https://github.com/smith-chem-wisc/MetaMorpheus); StavroX 3.6.6 (http://stavrox.com/).

For manuscripts utilizing custom algorithms or software that are central to the research but not yet described in published literature, software must be made available to editors and reviewers. We strongly encourage code deposition in a community repository (e.g. GitHub). See the Nature Portfolio guidelines for submitting code & software for further information.

Data

Policy information about availability of data

All manuscripts must include a data availability statement. This statement should provide the following information, where applicable:

- Accession codes, unique identifiers, or web links for publicly available datasets
- A description of any restrictions on data availability
- For clinical datasets or third party data, please ensure that the statement adheres to our policy

The mass spectrometry proteomics data for the cross-linking experiments of UBXD1/p97/Ub and UBXD1/HR23b generated in this study have been deposited to the ProteomeXchange Consortium via the PRIDE58 partner repository (https://www.ebi.ac.uk/pride/archive/) under the accession codes PXD039606 (UBXD1/p97/Ub) [https://www.ebi.ac.uk/pride/archive/projects/PXD039606] and PXD040984 (UBXD1/HR23b) [https://www.ebi.ac.uk/pride/archive/projects/PXD040984].

The atomic coordinates of the UBXD1-PUB-eUBX-C model generated in this study are provided as Supplementary Data file.
The published structures used in this study are available in the Protein Data Base under the following accession codes:
- ASPL:p9722 :pdb 5ifw [http://doi.org/10.2210/pdb5IFW/pdb].
- UBXD1-PUB14 : pdb 6sap [http://doi.org/10.2210/pdb6SAP/pdb].
- HR23b-UBL50 : pdb 1p1a [http://doi.org/10.2210/pdb1P1A/pdb].
- PNGase-PUB:p9751 : pdb 2hpl [http://doi.org/10.2210/pdb2HPL/pdb].
- p97 hexamer34 : pdb 3cf3 [http://doi.org/10.2210/pdb3CF3/pdb].
- Ubiquitin59 : pdb 1d3z [http://doi.org/10.2210/pdb1D3Z/pdb].

The AlphaFold model of UBXD1-fl used in this study is available in the AlphaFold Protein Structure Database under the accession code Q9BZV1 [https://alphafold.ebi.ac.uk/entry/Q9BZV1].

Source data are provided as a Source Data file.

Human research participants

Policy information about studies involving human research participants and Sex and Gender in Research.

Reporting on sex and gender	N/A
Population characteristics	N/A
Recruitment	N/A
Ethics oversight	N/A

Note that full information on the approval of the study protocol must also be provided in the manuscript.

Field-specific reporting

Please select the one below that is the best fit for your research. If you are not sure, read the appropriate sections before making your selection.

☒ Life sciences ☐ Behavioural & social sciences ☐ Ecological, evolutionary & environmental sciences

For a reference copy of the document with all sections, see nature.com/documents/nr-reporting-summary-flat.pdf

Life sciences study design

All studies must disclose on these points even when the disclosure is negative.

Sample size	Measures taken to verify reproducibility included to perform experiments at least three times (for exeptions, see below). Additionally, the number of repetitions for all experiments is reported in the respective figure legend or methods section. Although no statistical analysis was performed to predetermine sample size, but three biological replicates is considered standard practice for most biochemical assays.

Data exclusions	As revealed in the source data file, no data was excluded from the analyses. However, experiments that have undoubtedly not been run correctly due to e.g. technical issues were not included in the study (e.g. when a protein precipitates prior to measurements, or there are no detectable bands at all in Western Blot analyses).
Replication	Measures taken to verify reproducibility included to perform every experiment at least three times. After initial establishment and technical optimization of each method, all attempts at replication were successful. However, as we had to handle large proteins including mutants that could not be easily expressed, replication of NMR and ITC experiments was not possible due to very low protein concentrations. Because such large amounts of isotope-labelled proteins are needed, it is common for protein NMR experiments to not be replicated. Chemical Crosslinking of the ternary protein complex was also only performed once due to the elaborate procedure.
Randomization	The samples were not randomized in our study (not applicable here). Here, the main task was to biochemically characterize protein-protein interactions, so it was not possible to rearrange sample orders randomly.
Blinding	Blinding was not used in our study (not applicable here). Here, the main task was to biochemically characterize protein-protein interactions in vitro. In contrast to medical studies with human subjects where placebo and nocebo effects are to be expected, these effects do not occur in biochemical studies with isolated molecules. In contrast, blinding is not used in biochemical experiements because the handling conditions and procedures for each protein construct are very specific, need to be optimized for each protein construct and need to be followed precisely once established. The experimentator needs to know and be very famliar with these conditions and procedures and thus needs to know the content of each sample to apply the correct protocol. Application of the wrong handling or conditions can cause false-negative results due to protein unfolding or degradation. Therefore blinding is not possible in this type of study.

Reporting for specific materials, systems and methods

We require information from authors about some types of materials, experimental systems and methods used in many studies. Here, indicate whether each material, system or method listed is relevant to your study. If you are not sure if a list item applies to your research, read the appropriate section before selecting a response.

Materials & experimental systems

n/a	Involved in the study	
☐	☒	Antibodies
☐	☒	Eukaryotic cell lines
☒	☐	Palaeontology and archaeology
☒	☐	Animals and other organisms
☒	☐	Clinical data
☒	☐	Dual use research of concern

Methods

n/a	Involved in the study	
☒	☐	ChIP-seq
☒	☐	Flow cytometry
☒	☐	MRI-based neuroimaging

Antibodies

Antibodies used	primary antibodies:<br>- anti-GST, mouse, Santa Cruz Biotechnology (sc-57753, clone 3D4, lot. F1509), 1:1,000 (WB).<br>- anti-UBXD1, mouse, Abcam, (ab81555, clone 2F8-24, lot GR3296146-1), 1:1000 (WB).<br>- anti-UBXD1 (= anti-UBXN6), mouse, Acris Antibodies (5C3-1, clone 5C3-1, no lot #), 1:500 (WB).<br>- anti-p97/VCP, mouse, Santa Cruz Biotechnology (sc-57492, clone VCP 5, lots L1416 and L3120), 1:1000 (WB).<br>- anti-poly-Ub proteins, mouse, Merck (04-263, clone FK2, lot. 3277133), 1:2000 (WB).<br>- anti-Ufd1, mouse, BD Transduction Laboratories (611642, clone 19/Ufd1L, lot 9182092), 1:1000 (WB).<br>- anti-Npl4 (=anti-NPLOC4), rabbit, Atlas (HPA021560, lot 000014025, polyclonal), 1:1000 (WB).<br>- anti-penta-His, mouse monoclonal, Qiagen (34660, lot 163038868, no cone #), 1:1000 (WB).<br><br>secondary antibodies:<br>- anti-mouse IgG-horseradish peroxidase (HRP), sheep, Cytiva Life Sciences (NXA931, lot 17205275, polyclonal), 1:10,000 (WB).<br>- anti-mouse IgG (H+L)-HRP Conjugate, goat, Biorad (1706516, batch 64487942, polyclonal), 1:10,000 (WB).<br>- anti-rabbit IgG (H+L)-HRP Conjugate, goat, Biorad (1706515, batch 64425247, polyclonal), 1:10,000 (WB).
Validation	Validation of antibodies (primary and secondary) according to the manufacturer's website:<br>- anti-GST (Santa Cruz Biotechnology Inc., sc-57753) has been validated for use in western blotting to detect recombinant GST protein, as stated on the suppliers product page.<br>- anti-UBXD1 (Abcam, ab81555) has been validated for use in WB, as stated on the suppliers product page.<br>- anti-UBXD1 (Acris Antibodies 5C3-1) has been validated for use in Western blotting, Immunoprecipitation and Immunofluorescence), as stated on the suppliers product information sheet.<br>- anti-p97/VCP (Santa Cruz, sc-57492) has been validated for detection of VCP of mouse, rat, human and porcine origin by WB, IP, IF and IHC(P), as stated on the suppliers product page.<br>- anti-poly-Ub proteins (Merck, 04-263) has been published and validated for use in ELISA, Immunofluorescence (IF), Immunoprecipitation (IP) and Western Blotting (WB) for the detection of Ubiquitinylated proteins, as stated on the suppliers product page.<br>- anti-Ufd1 (BD Transduction Laboratories, 611642) has been validated for Western blot (Routinely Tested), Immunofluorescence

(Tested During Development) , as stated on the suppliers product page.
- anti-Npl4 (Atlas, HPA021560) has been validated for Immunohistochemistry (IHC), Western Blot (WB) and Immunofluorescence in Cell Lines (ICC-IF), as stated on the suppliers product page.
- anti-penta-His (Qiagen 34660) has been validated for Colony, dot and western blotting procedures, Screening for positive expression clones, Monitoring expression levels and stability of His-tagged proteins, Immunoprecipitation and ELISA, Immunocytochemistry and immunohistochemistry, as stated on the suppliers product page.
- anti-mouse IgG-horseradish peroxidase (Cytiva, NXA931) has been validated for Western Blotting, as stated on the suppliers product page.
- anti-mouse IgG (H+L)-HRP Conjugate (Biorad, 1706515) has been validated as blotting-grade, as stated on the suppliers product page.
- anti-rabbit IgG (H+L)-HRP Conjugate (Biorad, 1706515) has been validated as blotting-grade, as stated on the suppliers product page.

All antibodies used in this study are used in our laboratory for many years. Also the lots used in the current study were successfully used in other projects in the lab before. In addition, we validated all antibodies in combination with already validated recombinant proteins or cell lysates in Western Blot (WB) analyses, which already serves as validation to exclude unspecific binding or cross-reactivity.

Eukaryotic cell lines

Policy information about cell lines and Sex and Gender in Research

Cell line source(s)	HEK293 (Cellosaurus, RRID:CVCL_0045 [CLS]). Cell Line Genotyping/Profiling was performed on Aug. 07, 2019 by the company Microsynth using high-polymorphic short tandem repeat loci (STRs).
Authentication	Morphology check by microscope.
Mycoplasma contamination	The cells are routinely tested for mycoplasma contamination and were tested negative all times.
Commonly misidentified lines (See ICLAC register)	Commonly misidentified lines were not used.
